# Lytic Capsule-Specific *Acinetobacter* Bacteriophages Encoding Polysaccharide-Degrading Enzymes

**DOI:** 10.3390/v16050771

**Published:** 2024-05-13

**Authors:** Peter V. Evseev, Anastasia S. Sukhova, Nikolay A. Tkachenko, Yuriy P. Skryabin, Anastasia V. Popova

**Affiliations:** 1 Shemyakin-Ovchinnikov Institute of Bioorganic Chemistry, Russian Academy of Sciences, 117997 Moscow, Russia; ntkachenko037@gmail.com; 2 State Research Center for Applied Microbiology and Biotechnology, City District Serpukhov, Moscow Region, 142279 Obolensk, Russia; schurova123@gmail.com (A.S.S.); sjurikp@gmail.com (Y.P.S.); 3 Pirogov Russian National Research Medical University, 117997 Moscow, Russia

**Keywords:** *Acinetobacter* spp., *A. baumannii*, bacteriophage, receptor-binding protein, tailspike protein, depolymerase, capsular polysaccharide, polysaccharide-degrading enzyme, polysaccharide-modifying enzyme, K locus, capsular type

## Abstract

The genus *Acinetobacter* comprises both environmental and clinically relevant species associated with hospital-acquired infections. Among them, *Acinetobacter baumannii* is a critical priority bacterial pathogen, for which the research and development of new strategies for antimicrobial treatment are urgently needed. *Acinetobacter* spp. produce a variety of structurally diverse capsular polysaccharides (CPSs), which surround the bacterial cells with a thick protective layer. These surface structures are primary receptors for capsule-specific bacteriophages, that is, phages carrying tailspikes with CPS-depolymerizing/modifying activities. Phage tailspike proteins (TSPs) exhibit hydrolase, lyase, or esterase activities toward the corresponding CPSs of a certain structure. In this study, the data on all lytic capsule-specific phages infecting *Acinetobacter* spp. with genomes deposited in the NCBI GenBank database by January 2024 were summarized. Among the 149 identified TSPs encoded in the genomes of 143 phages, the capsular specificity (K specificity) of 46 proteins has been experimentally determined or predicted previously. The specificity of 63 TSPs toward CPSs, produced by various *Acinetobacter* K types, was predicted in this study using a bioinformatic analysis. A comprehensive phylogenetic analysis confirmed the prediction and revealed the possibility of the genetic exchange of gene regions corresponding to the CPS-recognizing/degrading parts of different TSPs between morphologically and taxonomically distant groups of capsule-specific *Acinetobacter* phages.

## 1. Introduction

Hospital-acquired or nosocomial infections caused by antibiotic-resistant microorganisms have become a recognized concern in the modern healthcare system globally. The genus *Acinetobacter* comprises strictly aerobic, catalase-positive, oxidase-negative, non-fermentative, Gram-negative bacteria that belong to both environmental non-pathogenic species and clinically relevant species, causing various infectious complications, especially in intensive care units, severe ill patients, and patients with prolonged hospitalization [1,2,3]. *Acinetobacter baumannii* is a representative of the ESKAPE group (*Enterococcus faecium*, *Staphylococcus aureus*, *Klebsiella pneumoniae*, *Acinetobacter baumannii*, *Pseudomonas aeruginosa*, and *Enterobacter* spp.), the members of which are characterized by resistance to multiple classes of antibiotics and are the leading cause of healthcare-associated infections worldwide [4,5]. *Acinetobacter pittii* and *Acinetobacter nosocomialis*, included in *A. calcoaceticus–A. baumannii* complex, and some other *Acinetobacter* spp. can also cause different nosocomial complications [6]. Carbapenem-resistant *A. baumannii* strains have been classified as critical priority nosocomial pathogens for which the development of new antibacterials is urgently needed [7]. Therefore, the search for alternative approaches to control the spread of antibiotic-resistant *Acinetobacter* spp. is a subject of increasing interest. In this regard, the application of lytic bacteriophages (phages) and phage-encoded enzymes is a possible solution.

The strategies for the interaction of phages with bacterial cells are very diverse. Phage infection is initiated by the specific recognition and attachment of receptor-binding proteins (RBPs) to certain surface structures of their bacterial hosts, such as outer membrane proteins, lipopolysaccharide/lipooligosaccharide components, and capsular polysaccharides [8,9]. There are two types of RBPs typical for phages belonging to the class *Caudoviricetes*—that of tail fibers and tailspikes [9,10]. Both types are homotrimers, meaning they consist of three of the same polypeptide chains encoded in a phage genome [9,11,12]. The *N*-termini of phage RBPs are attached to the phage particles, whereas the remaining parts of these structures participate in recognition and binding to the host receptors [9,13]. In contrast to tail fibers, tailspike proteins (TSPs) are shorter and possess enzymatic activity toward bacterial carbohydrate surface structures [9,11]. TSPs have a modular structure including a domain displaying hydrolase, lyase, or esterase activity [9,13].

Most clinically relevant strains of *Acinetobacter* spp. produce a thick protective layer of capsular polysaccharides (CPSs), which are major virulence determinants and contribute to bacterial cell survival properties [14,15,16]. CPSs are polymers consisting of repeating oligosaccharide units (K units) that can differ from each other by the structures and numbers of monosaccharides and by the linkages between these monosaccharides and K units [17,18,19,20]. Such diversity of CPS structures is mediated by variability in the chromosomal loci responsible for capsule biosynthesis (K loci, KL) [21,22]. To date, more than 240 KL types have been bioinformatically predicted in *Acinetobacter* spp. genome assemblies deposited in the National Center for Biotechnology Information (NCBI) database [22,23]. The CPS structures of more than 64 *A. baumannii* capsular types (K types) have already been established [24].

Over the past years, several research groups have demonstrated that CPSs are the primary receptors for *Acinetobacter* phages carrying TSPs with polysaccharide-degrading activities [25,26,27,28,29,30,31,32,33,34]. Almost all these phages form plaques with halos expanding with time on the host bacterial lawns, as well as depolymerase-carrying phages that infect other bacterial species [35,36]. The exception is phages encoding polysaccharide-modifying enzymes, such as tailspike deacetylase, which does not cause the total cleavage of the corresponding CPS, but only the *O*-acetylation of one of the sugar residues; therefore, phages form plaques without visible halos [30].

In this work, we summarized data on all lytic capsule-specific phages within 233 *Acinetobacter* bacterial viruses, the genomes of which were deposited in the NCBI GenBank database by January 2024. First, all analyzed *Acinetobacter* phages were divided into several clusters using phylogenetic analysis and intergenomic comparisons. Then, groups containing lytic phages carrying TSPs with polysaccharide-depolymerizing/modifying activities, that is, capsule-specific phages, were identified. For each such group, we provided information on phages encoding TSPs with an established and described mechanism of the specific cleavage/modification of corresponding *Acinetobacter* CPSs or with predicted by the authors in the cited articles specificity toward a certain K type (K specificity). In addition, we predicted K specificity for some phages within each group based on the high percentage of phage-encoded TSP sequences or their structural similarity and phylogenetic relationship to a sequence of TSP with an established mechanism of enzymatic activity and/or by KL identification in genomes of *Acinetobacter* host strains if corresponding sequencing data or assembles were available.

Since the structures of TSPs determine the ability of phages to recognize the corresponding CPSs, the prediction of the K specificity of phages carrying these proteins using bioinformatics, phylogenetic analyses, and AlphaFold (AF) modelling is very important from the point of view of their further practical usage.

## 2. Materials and Methods

### 2.1. Data Collection and Database Construction

Phage genomic sequences were downloaded from the NCBI GenBank PHG and NCBI Genome databases (https://www.ncbi.nlm.nih.gov, accessed on 10 January 2024). Downloaded sequences were checked for the presence of doubles and small fragments that did not contain structural, replication, or lysis genes using Geneious Prime v2023.2.1 (Biomatters, Inc., Auckland, New Zealand) tools and BLAST search [37] against the NCBI nt database. Protein structures were downloaded from the Research Collaboratory for Structural Bioinformatics Protein Data Bank (RCSB PDB) (https://www.rcsb.org, accessed on 10 January 2024). BLAST databases were constructed using the “makeblastdb” BLAST command.

### 2.2. Protein Structure Modeling

All protein structures were modelled with AlphaFold v2.3.2 (AF2) [38,39] using full databases and the command line parameters “--monomer” (for monomeric protein) or “--multimer” (for protein complexes). The best-ranked structures were selected for further study. Protein structures were superimposed and visualized using Pymol 2.5.4 (Schrödinger Inc., New York, NY, USA).

### 2.3. Functional Annotation

Phage genomes were partially reannotated with the assistance of Glimmer 3.0.2 [40], which was used for the detection of protein-coding open-reading frames (ORFs). The functions of phage proteins were predicted using a BLAST homology search (E-value < 1 × 10^−5^), the HHpred search using PDB70_mmcif_2023-06-18, PfamA-v35, UniProt-SwissProt-viral70_3_Nov_2021, and NCBI_Conserved_Domains (CD)_v3.19 databases (HHpred probability > 95%) [41], and the DALI search [42].

Multiple alignments of primary amino acid sequences were obtained using MAFFT 7.48 [43] with default settings and using the L-INS-i algorithm. Phylogenetic analysis based on sequence alignments was performed using IQ-TREE v2.2.5 [44] and the “--alrt 10000 -B 5000” command line parameters. The resulting consensus trees with bootstrap support values (10,000 replicas) were visualized using iTOL v6 [45]. Protein structural similarity was assessed using the DALI Z-score [46]. Phylogenetic trees based on structural similarity were obtained using the built-in DALI tools. Intergenomic comparison was performed using VIRIDIC v1.1 [47] with default settings.

### 2.4. Identification of K Locus Sequences in Acinetobacter Genome Assemblies

KL identification in *A. baumannii* genome assemblies was performed using Kaptive (https://kaptive-web.erc.monash.edu/, accessed on 12 February 2024) [22,48]. For *A. baumannii* strains B115 (SRR24880467), B577 (SRR24880644), and B711 (SRR24880838), draft assemblies were performed. Sequencing reads were downloaded from the NCBI Sequence Read Archive (https://www.ncbi.nlm.nih.gov/sra, accessed on 22 January 2024) using the NCBI SRA Toolkit (https://github.com/ncbi/sra-tools, accessed on 22 January 2024) tool fasterq-dump v. 3.0.10 with -S option enabled [49]. The assembly was carried out using unicycler v. 0.5.0 with spades 3.15.5 without read filtering with default parameters [50,51]. The identified KL were also verified with BLASTn against known sequences of capsule biosynthesis loci deposited in the NCBI database using a cut-off of >95% combined coverage with >95% nucleotide sequence identity.

## 3. Results

### 3.1. General Characterization of Genomic Data on Acinetobacter Phages

In January 2024, the NCBI Genome and GenBank PHG (bacteriophage sequences) databases contained 233 deduplicated complete and partial double-stranded DNA genomes labeled as “*Acinetobacter* phage” and appearing to belong to the class *Caudoviricetes*. These 233 sequences were retained for further analysis.

The size of full-length genomic sequences varied from 11,885 base pairs (bp) in the case of phage Phanie to 234,900 bp (phage vB_AbaM_ME3). The GC content ranged from 30.8% (phage vB_AbaM_ME3) to 54.6% (phage NJ01). *Acinetobacter* phages with deposited genomes were isolated around the world, namely in China (70 phages), Russia (49), USA (24), South Korea (17), Poland (14), Portugal (11), France (6), Thailand (5), Taiwan (5), Turkey (4), Spain (4), Benin (3), United Kingdom (3), Belgium (2), Canada (2), Egypt (2), Israel (2), Libya (2), Finland (1), Germany (1), India (1), Iran (1), Ireland (1), Mexico (1), Pakistan (1), and Switzerland (1). Information about the isolation source (primarily sewage and wastewater) was included in the descriptions of 146 genomic sequences. Among 233 *Acinetobacter* spp. phages, *A. baumannii* as a bacterial host was indicated for two-hundred and three phages, six phages were isolated on *A. pittii*, three phages were specific to *Acinetobacter johnsonii* and to *Acinetobacter calcoaceticus*, and one phage was specific to *A. nosocomialis*, *Acinetobacter beijerinckii*, *Acinetobacter halotolerans*, and *Acinetobacter soli.* Thirty-nine phage sequences were labeled as members of the class *Caudoviricetes*, and one-hundred and ninety-four sequences contained more detailed information about phage taxonomy.

### 3.2. Cluster and Phylogenetic Analyses of Acinetobacter Phages

NCBI Taxonomy does not fully match the official ICTV (International Committee of Taxonomy of Viruses) Taxonomy. For example, the genus *Friunavirus* (*Viruses*; *Duplodnaviria*; *Heunggongvirae*; *Uroviricota*; *Caudoviricetes*; *Autographiviridae*; *Beijerinckvirinae*) recognized by the ICTV comprises only 23 officially approved species (https://ictv.global/taxonomy, accessed on 12 January 2024), whereas the NCBI Genome and GenBank PHG databases contain 80 genomic sequences as belonging to the genus *Friunavirus*. Classification of phages according to ICTV criteria (“taxonomic classification”) was not the goal of the present study, but grouping phages by similarity may facilitate the presentation of the results of analyses of individual proteins.

In this work, a cluster analysis of 233 *Acinetobacter* phages was performed using the intergenomic similarity calculator VIRIDIC, a tool recommended by the ICTV for the classification of viruses [47,52]. In general, the clustering performed (Figure S1) grouped genomic sequences in a manner similar to the official and NCBI taxonomy. An arbitrary 20% intergenomic similarity threshold was applied to all *Acinetobacter* phages except representatives of the family *Schitoviridae*, resulting in 27 clusters numbered conditionally according to phage morphology (first podovirus, then myovirus and siphovirus morphology), phage lifestyle (first lytic, then temperate phages), and alphabetical order of the names of taxonomic groups (Table 1).

**Figure 1:**
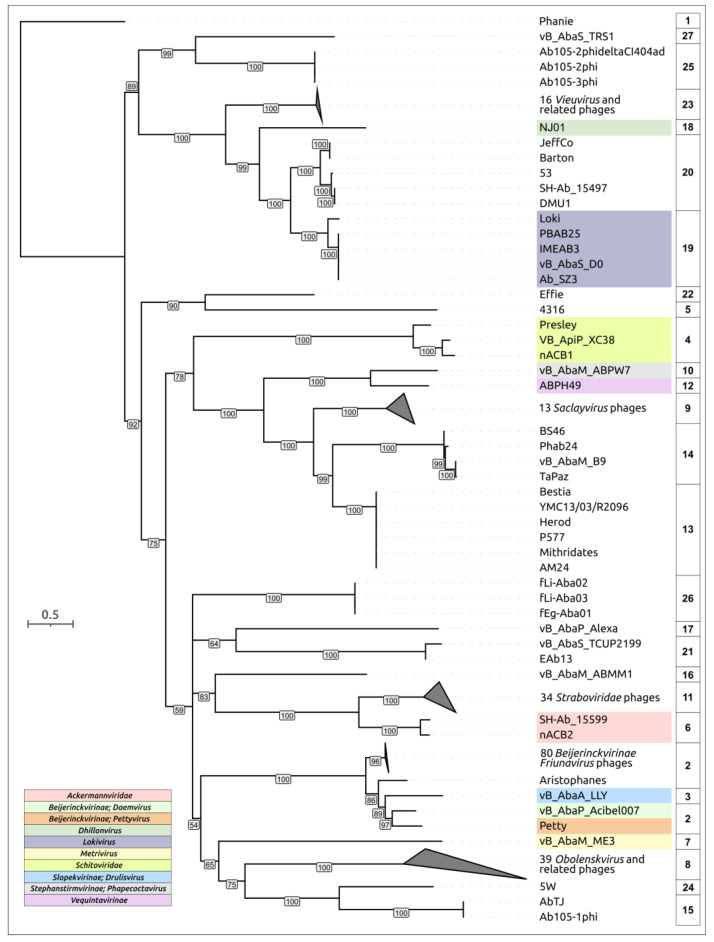
Phylogenetic tree based on 232 sequences of major capsid proteins found in the genomes of *Acinetobacter* phages. Bootstrap values are shown near their branches. Branches with bootstrap support lower than 50% were deleted. The scale bar shows 0.5 estimated substitutions per site, and the trees were rooted to *Acinetobacter* phage Phanie. Phage taxonomy is shown in the labels and legends. Phage clustering is indicated in the column to the right of the tree labels. Some branches collapsed. The same tree with expanded branches is shown in Figure S2.

To confirm the clustering of *Acinetobacter* phages based on intergenomic similarity, a phylogenetic analysis was performed using major capsid protein (MCP) sequences. A full-length MCP-coding sequence was not found in the genome of phage Ab1656-2; therefore, 232 sequences were used for analysis to construct the phylogenetic tree. As a result, all *Acinetobacter* phages were grouped almost identically to clustering by intergenomic similarity (Figure 1).

### 3.3. Determination of Groups of Lytic Acinetobacter Phages Carrying Tailspikes with Polysaccharide-Degrading Activities

Among the 27 groups of *Acinetobacter* phages obtained by bioinformatic and phylogenetic analyses, the lytic phages were attributed to 18 groups because of the absence of genes encoding integrases or lysogeny-related proteins in their genomes. The proteins encoded by lytic *Acinetobacter* phages were retained for further analysis. In most cases, the genes encoding RBPs are located in the structural or tail modules of phage genomes. To determine whether the tail proteins are tailspikes with polysaccharide-degrading activities, BLASTp analysis [37] and HHpred search [41] were performed.

In the case of TSPs with CPS-depolymerasing activities, the proteins share structural similarity with different phage carbohydrate-degrading enzymes (hydrolases or lyases) and other tailspikes with experimentally determined structures. According to HHpred analysis, the pectate_lyase_3 (PF12708) and glyco_hydro_28 (PF00295) conserved Pfam motifs are usually identified in the amino acid sequences of these proteins. In the case of TSPs with CPS-modifying activities or tailspike esterases, the proteins share structural similarity with SGNH-hydrolase domain-containing proteins, such as GDSL-like lipase/acylhydrolase family proteins or sialic acid-specific acetylesterases.

The analyses indicated that TSPs with polysaccharide-degrading activities are encoded in the genomes of *Acinetobacter* phages, which belong to cluster 2 (subfamily *Beijerinckvirinae*), cluster 3 (subfamily *Slopekvirinae*, genus *Drulisvirus*), cluster 6 (the family *Ackermannviridae*), cluster 8 (genus *Obolenskvirus* and related phages), cluster 10 (subfamily *Stephanstirmvirinae*, genus *Phapecoctavirus*), cluster 12 (subfamily *Vequintavirinae*), clusters 13 and 14 (unclassified *Caudoviricetes* with myovirus morphology), and clusters 20 and 22 (unclassified *Caudoviricetes* with siphovirus morphology).

### 3.4. Specificity of TSP-Carrying Acinetobacter Phages

In total, the groups of lytic capsule-specific *Acinetobacter* phages combined 143 bacterial viruses. The established or predicted K specificity of these phages and the features of their interaction with bacterial hosts are discussed in detail below.

#### 3.4.1. Specificity of Phages Belonging to the Subfamily *Beijerinckvirinae* (Cluster 2) for Different *Acinetobacter* K Types

The subfamily *Beijerinckvirinae* of the family *Autographiviridae* constitutes the largest group of known phages infecting *Acinetobacter* spp. and comprises 83 bacterial viruses with genomes that have been deposited in the NCBI GenBank by January 2024. Eighty out of these phage genomes were attributed to the genus *Friunavirus*, applying the 70% genus demarcation threshold of intergenomic similarity [52]. The phage Aristophanes, which is closely related to the representatives of the genus *Friunavirus*, should be assigned to a different genus of the subfamily *Beijerinckvirinae* [30]. The *Acinetobacter* phage Petty (or *Pettyvirus petty*) [25] was officially assigned to the genus *Pettyvirus*, and the *Acinetobacter* phage vB_AbaP_Acibel007 [68] was classified as *Daemvirus acibel007* (the genus *Daemvirus*).

Most of the Friunaviruses infect *A. baumannii*, but there are also four representatives of the genus that interact with *A. pittii* (phages vB_Api_3043-K38, vB_ApiP_P1, vB_ApiP_P2, and vB_AP_P1489). The phages Aristophanes and vB_AbaP_Acibel007 infected *A. baumannii* strains. The bacterial host for the phage Petty belongs to the species *A. nosocomialis.*

**Figure 2:**
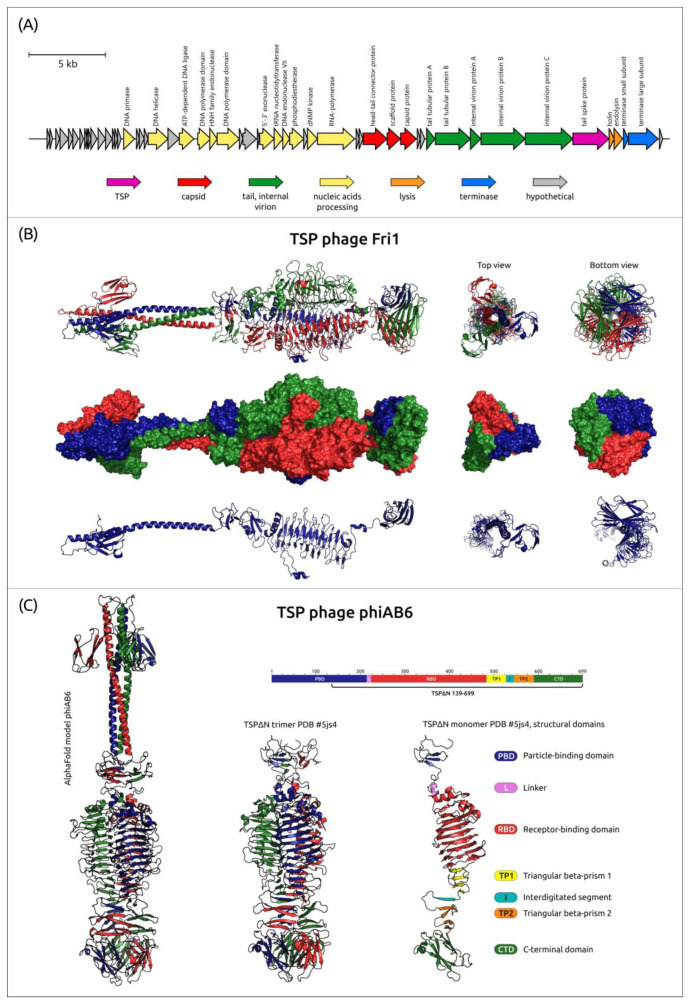
(**A**) Genetic map of *A. baumannii* phage Fri1 (Genbank accession number: KR149290). Arrows indicate the direction of transcription. The scale bar indicates the length of the nucleotide sequence. Gene functions are shown in the labels and legends. (**B**) AF model of phage Fri1 TSP trimer, where the three monomers are colored red, green, and blue. In the figure, from top to bottom: ribbon presentation of the trimer, surface-rendered presentation of the trimer, and ribbon presentation of the monomer. The top view corresponds to the view from the *N*-terminus and the bottom view corresponds to the view from the C-terminus of the TSP. (**C**) AF model and experimentally determined structure of phage phiAB6 TSPΔN (PDB ID: 5JS4) lacking most of the particle-binding domain. In the figure, from left to right: AF model of TSP trimer shown as three-colored ribbons, TSPΔN-trimer, where each monomer is marked with its own color, and monomer, where structural models are marked in different colors as shown in the sequence map above, according to Lee et al. [27].

The genomes of all *Beijerinckvirinae* phages contain only one gene encoding TSP. This gene is located at the end of the phage genome’s structural module upstream of the genes responsible for proteins associated with bacterial cell lysis and with the packaging of phage DNA [25,26,30,31,34] (Figure 2A).

The alignment of 83 amino acid sequences of *Beijerinckvirinae* phage TSPs showed a high level of similarity (79.5% pairwise identity, PI) between their *N*-terminal parts (corresponding to 1–187 aa residues of TSP encoded in the genome of phage Fri1), which are responsible for the binding of the proteins to the phage particles. The similarity between the remaining parts of TSPs (in phage Fri1 it is 188–783 aa residues) is much lower (PI 11.3%), indicating that their diversity determines the specific recognition and degradation/modification of various CPSs [13,36,95,123,124,125].

To date, the atomic structures of TSPs encoded in the genomes of Friunaviruses Fri1 (Protein Data Bank identifier or PDB ID: 6C72), vB_AbaP_AS12 (PDB ID: 6EU4), phiAB6 (PDB ID: 5JS4) [27], APK09 (PDB ID: 8OQ0) [34], APK14 (PDB ID: 8OQ1) [34], APK16 (PDB ID: 8OPZ) [34], and vB_ApiP_P1 (PDB ID: 6E1R) were established using X-ray crystallography. The experimentally determined structures available in PDB lack the full-sized *N*-terminal particle-binding parts due to the aggregation or poor solubility of full-length proteins. However, AF [38,39] modeling allows us to suggest that the *N*-terminal part of the proteins can include a β-sandwich domain followed by an α-helix (Figure 2B,C). In turn, AF predictions and HHpred searches indicate the presence of domains characteristic of carbohydrate-degrading enzymes and reveal different structural architectures of the remaining parts (further referred as to “CPS-recognizing/degrading parts”) of TSPs (the examples are presented in Supplementary File S1). The experimentally determined structure of the phage phiAB6 tailspike hydrolase lacking most of the particle-binding domain (TSPΔN, PDB ID: 5JS4) [27] is shown in Figure 2C as an example demonstrating the modular organization of TSPs.

Currently, the mechanisms of enzymatic activity of tailspike depolymerases encoded in the genomes of Friunaviruses phiAB6 [27], vB_AbaP_APK2 [31], APK09 [34], vB_AbaP_APK14 [34], APK16 [34], Fri1 [126], vB_AbaP_APK26 [70], vB_AbaP_AS12 [126], vB_AbaP_APK32 [31], vB_AbaP_APK37 [31], APK37.1 [34], vB_AbaP_APK44 [31], vB_AbaP_APK48 [31], APK86 [34], vB_AbaP_APK87 [31], vB_AbaP_APK89 [31], vB_AbaP_APK116 [31], APK127v [34], and vB_AbaP_APK128 [34] have been established. All these TSPs were shown to be specific glycosidases that cleaved the corresponding *A. baumannii* CPSs by a hydrolytic mechanism with the production of monomers and/or oligomers of the repeating K units. Notably, the protein encoded in the genome of the phage Aristophanes (gp41, QNO11465) with a characterized mechanism of action was the first described tailspike deacetylase, which did not cause the total cleavage of the CPS to monomers or oligomers of the K unit, but only *O*-acetylation of one of the K26 sugar residues [30].

Table 2 summarizes the data on all capsule-specific *Beijerinckvirinae* phages with genomes deposited in the NCBI GenBank database. This table provides information on the phages encoding TSPs with established and described mechanisms of the specific cleavage/modification of corresponding Acinetobacter CPSs (marked in red), on the phages the K specificity of which was predicted by the authors in the cited articles based on KL identification in genomes of Acinetobacter host strains, on the determination of the sensitivity of an Acinetobacter strain with a known K type to a phage or a high percentage of phage-encoded TSP sequence similarity to a sequence of a TSP with an established mechanism of enzymatic activity (marked in green), and finally, on phages the K specificity of which was predicted in this work based on a high percentage of phage-encoded TSP sequence similarity and its phylogenetic relationship to a sequence of a TSP with a determined substrate specificity or KL identification in genomes of Acinetobacter host strains (marked in blue). 

According to the BLASTp analysis, TSP phiAB6_gp40 (ALA12264), with a determined substrate specificity toward K2 CPS of *A. baumannii* 54149 [27], shares a high level of similarity with the proteins encoded in genomes of Friunaviruses vB_AbaP_WU2001 (QVQ34730), vB_AbaP_D2 (AVP40472), vB_AbaP_D2M (QFG15400), WCHABP5 (ARQ94869), SWH-Ab-3 (YP_009949108), ABp57 (WNV46778), vB_AbaP_100 (gene product corresponding to TSP was predicted in this work), vB_AbaP_B3 (ASN73401), and vB_AbaP_PMK34 (QGF20174) at the amino acid level (the coverage obtained to an E-value of 0 was 100% with identities more than 95%). The CPS-recognizing/degrading part of phiAB6_gp40 shares a high percentage of amino acid similarity with the proteins encoded in *Acinetobacter* phage SH-Ab 15599 (AXF41547), belonging to the family *Ackermannviridae*, and *Acinetobacter* phages Abp95 (QYC51728) and vB_AbaM_fThrA (WVH13570), which are assigned to the genus *Obolenskvirus*. The similarity of the amino acid sequences of these proteins indicates that they most likely interact specifically with the CPS of the same structure, namely K2 CPS. In the case of phages vB_AbaP_B3 and vB_AbaP_PMK34, the authors established that the *A. baumannii* host strains which are sensitive to these phages indeed belong to the K2 capsular type [26,128].

**Figure 3:**
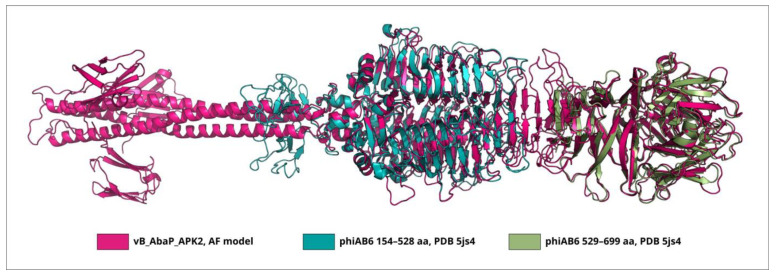
Superimposition of the CPS-recognizing/degrading part of the phiAB6 TSP trimer (PDB ID: 5JS4) onto the AF model of TSP vB_AbaP_APK2_gp43. The RMSD of subdomains of CPS-recognizing/degrading parts colored teal and olive was 0.70 Å and 0.81 Å.

Interestingly, TSP phiAB6_gp40 does not share a high level of similarity with TSP vB_AbaP_APK2_gp43 (AZU99242) [31], which also has an established mechanism of enzymatic activity toward K2 CPS (the coverage obtained to an E-value of 5 × 10^−97^ was 87% with an identity 38.01%). However, the superimposition of the experimentally determined CPS-recognizing/degrading part of TSP phiAB6_gp40 onto the AF-predicted structure of TSP vB_AbaP_APK2_gp43 yielded an RMSD (root-mean-square deviation) less than 1 Å (Figure 3), indicating that the structures of the CPS-recognizing/degrading parts of these proteins are very similar.

It is noteworthy that TSP vB_AbaP_APK2_gp43 interacts with both K2 and K93 CPSs due to the similarity of their K units, which have the same main chains and differ only in their side chain structures, and the identity of the linkages between the K2 and K93 K units [31]. K2 CPS-specific vB_AbaP_APK2_gp43 was identical to the TSPs encoded by phages vB_AbaP_APK2-2 (AZU99292), vB_AbaP_APK93 (AZU99342), and BM12 (UYE92398). vB_AbaP_APK2_gp43 was almost identical (more than 99% of identity at the amino acid level) to the proteins encoded in the genomes of Friunaviruses YZ2 (WPD4945299), vB_AbaP_B4 (WNO29457), IME-200 (YP_009216489), and was highly similar (more than 96% of identity at the amino acid level) to the proteins encoded in the genomes of phages AbpL (UVD42134), pB3074 (WID41884), SH-Ab 15519 (YP_009598268), Abgy2021-6-2 (WPF70339), Ab124 (QMP19165), vB_AbaP_ABWU2101 (UFJ83440), MRABP9 (WAK44760), and vB_AbaP_APK81 (QNO11418). vB_AbaP_APK2_gp43 was also homologous to the protein of Friunavirus vB_AbaP_AGC01 (QIW86364, the coverage obtained to an E-value of 0 was 100% with an identity of 91.92%). In addition, the CPS-recognizing/degrading part of vB_AbaP_APK2_gp43 was homologous to the protein encoded by *Acinetobacter* phage NJ02 (WJZ47808), which belongs to the genus *Obolenskvirus*. Based on the high percentage of similarity of the amino acid sequences of all the phage-encoded proteins listed above to the sequence of TSP vB_AbaP_APK2_gp43 with an established mechanism of enzymatic activity, it can be assumed that these proteins also interact, specifically, with K2 CPS.

The regions in the genome of the phage AbKT21phiIII [56] corresponding to the amino acid sequence of the TSP were predicted in this study (Table 2). The CPS-recognizing/degrading part of the predicted TSP shares a high percentage of similarity with the CPS-recognizing/degrading parts of the proteins encoded in the genomes of phages WCHABP1 (ARQ94726), Abp9 (QEA11050), P1068 (WHB31253), and vB_AbaM_IME512 (AYP69084), which belong to the genus *Obolenskvirus* (from 86.06% to 94.68% of identity at the amino acid level). The TSP of the phage AbKT21phiIII also shares an average level of similarity with the depolymerase encoded by Friunavirus APK127v (URQ05189, the coverage obtained to an E-value of 9 × 10^−163^ was 89% with an identity of 52.53%), with an established mechanism of enzymatic activity toward *A. baumannii* K127 CPS [34]. Using Kaptive [22,48], the available draft genome sequence of the phage AbKT21phiIII bacterial host *A. baumannii Ab*KT722 (NCBI accession number: RXIN00000000) [56] was found to include the KL3 locus and was, therefore, predicted to produce a CPS with a K3 structure. The BLAST comparison revealed a 99% identity of identified in the genome A. baumannii AbKT722 KL3 with the previously described KL3 capsule biosynthesis gene cluster (GenBank accession number KF793926). Thus, the K3 specificity of the phage AbKT21phiIII was determined in this study by predicting KL3 in the genome of the *A. baumannii* bacterial host.

The CPS-recognizing/degrading part of the protein encoded by Friunavirus vB_AbaP_IME546 (QFR59034) shares an 88% identity with the amino acid sequence of the TSP of *Acinetobacter* phage Mithridates (QVG63948), the bacterial host of which is *A. baumannii* LUH5533, belonging to the K7 capsular type [144]. The similarity of the CPS-recognizing/degrading parts of these proteins indicates that they are presumed to recognize and degrade the CPS of the same structure, namely K7 CPS. vB_AbaP_IME546 also shares an average level of similarity with the TSP encoded by *Friunavirus* vB_AbaP_APK89 (QGK90394, the coverage obtained to an E-value of 2 × 10^−165^ was 71% with an identity of 47.44%), which has a determined mechanism of action towards *A. baumannii* CPS with a K89 structure [31].

**Figure 4:**
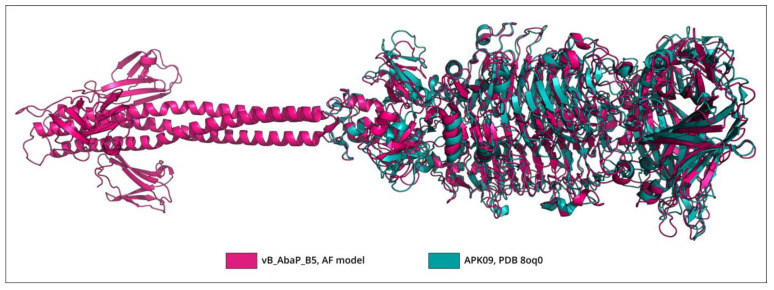
Superimposition of the CPS-recognizing/degrading part of the phage APK09 TSP trimer (PDB ID: 8OQ0) onto the AF model of the phage vB_AbaP_B5 TSP trimer. The RMSD calculated as an average across subdomains of the CPS-recognizing/degrading parts was 0.88 Å.

The BLASTp analysis revealed that TSP APK09_gp48 (UAW09804), which has a determined substrate specificity toward K9 CPS [34], was almost identical to the protein encoded by Friunavirus vB_AbaP_B1 (ASN73353, the coverage obtained to an E-value of 0 was 100% with an identity of 99.08%) and shares a fairly low level of similarity with the TSP encoded by Friunavirus vB_AbaP_B5 (ASN73455, the coverage obtained to an E-value of 3 × 10^−147^ was 95% with an identity of 44.49%). The bacterial hosts of phages vB_AbaP_B1 and vB_AbaP_B5 belong to the same K9 capsular type [26] as the bacterial host of phage APK09, *A. baumannii* B05 [34]. Despite the low level of amino acid similarity between TSPs encoded by phages APK09 and vB_AbaP_B5, the structures of these tailspikes are apparently very similar. The superimposition of the experimentally determined CPS-recognizing/degrading part of APK09_gp48 onto the AF-predicted structure of vB_AbaP_B5_gp47 yielded an RMSD of 1.3 Å and less, depending on the subdomain of the CPS-recognizing/degrading part used (Figure 4).

The closest homolog of TSP APK14_gp49 (AYR04394) with an established enzymatic activity toward K14 CPS [34] was the TSP of Friunavirus AB_SZ6 (URQ05102, the coverage obtained to an E-value of 0 was 100% with an identity of 90.36%). The CPS-recognizing/degrading part of APK14_gp49 also shares amino acid similarity with the proteins encoded in *Acinetobacter* phages YMC13/03/R2096 (AIW02768) and P577 (WNT46259).

The CPS-recognizing/degrading part of TSP APK16_gp47 (UAW09859), which has an established mechanism of enzymatic activity [34], has no homologs among the lytic phage depolymerases deposited in GenBank.

The bacterial hosts of phages APK15 and APK20 are *A. baumannii* MAR 15-4788 and MAR 14-595, the genomes of which include KL15 and KL20, respectively (unpublished data). The CPS-recognizing/degrading parts of the proteins APK15_gp48 (UAW10027) and APK20_gp52 (UAW10085) have no homologs among the lytic phage depolymerases deposited in GenBank.

The TSP Fri1_gp49 (AKQ06854), which degrades K19 CPS [126], was highly identical to TSPs encoded in the genomes of Friunaviruses vB_AbaP_AS11 (AQN32697) and vB_AbaP_PE21 (ULG00671), which were isolated on the same host strain, *A. baumannii* 28, as the phage Fri1 [25]. The purified recombinant protein corresponding to the deletion mutant lacking the *N*-terminal domain of vB_AbaP_AS11_gp45 forms an opaque halo (zone of CPS depolymerization) on the bacterial lawn of *A. baumannii* 28 [25].

The CPS-recognizing/degrading part of the TSP encoded in the genome of Friunavirus vB_AbaP_APK26 (QQO97001), which has an established mechanism of action [70], shares a high level of similarity with the TSP encoded in the genome of Obolenskvirus vB_AbaM_AB3P2 (WOZ14994).

The TSP encoded in the genome of the phage vB_AbaP_AS12 (APW79830), which has a determined mechanism of enzymatic activity toward K27 CPS [126], only shares an average level of similarity with TSPs of the phage vB_AbaP_APK128 (QVD48888), which infects *A. baumannii* with a K128 CPS structure [34], and phage phiAB1 (ADQ12745).

The TSP of Friunavirus vB_AbaP_APK32, with an established substrate specificity toward K32 CPS [31], was highly similar to the TSPs of phages Pipo (QQO92973) and Paty (QQM15083) (identity more than 96%) and to the proteins encoded in the genomes of phages vB_AbaP_ZHSHW (UPT53561) and vB_AbaP_EPab_B (WGV35678) (identity more than 94%). Based on the high percentage of the similarity of the amino acid sequences of all these proteins, it can be assumed that they interact specifically with the CPS of the same structure.

The K37-specific TSP APK37.1_gp49 (UAW07728) is encoded in the genome of phage APK37.1, which was found to infect *A. baumannii* strains that carry not only KL37 but also KL116 and a subset of *A. baumannii* isolates carrying KL3/KL22 with a single-base deletion in the *gtr6* gene, causing the loss of Gtr6 glycosyltransferase (K3-v1 capsular type) [136]. The mechanism of the specific cleavage of K37 and K3-v1 *A. baumannii* CPSs by APK37.1_gp49 was determined [34]. The possibility that APK37.1_gp49 specifically interacts with K3-v1, K37, and K116 CPSs can be explained by the fact that the arrangements of the main chain residues as well as the glycosidic linkages between the oligosaccharide units are very similar in CPSs of K3-v1, K37, and K116 structures [136]. Interestingly, the CPS-recognizing/degrading part of the TSP APK37.1_gp49 shares no similarity with the TSP vB_AbaP_APK116_gp43 (QHS01530), which has an established substrate specificity towards K116 CPS. The depolymerase was also not closely related to the K37-specific tailspike depolymerase vB_AbaP_APK37_gp44 (AZU99445, the coverage obtained to an E-value of 0 was 99% with an identity of 53.66%). In turn, the closest homolog for vB_AbaP_APK37_gp44 was the protein encoded in the genome of the phage AbTP3phi1 (UNI74976, the coverage obtained to an E-value of 0 was 100% with an identity of 96.23%). The phage AbTP3phi1 was shown to infect the A. baumannii TP3, which encodes the KL116 capsule locus [32], as determined by Kaptive [48]. However, given the high level of amino acid similarities between sequences of the TSPs encoded in the genomes of phages vB_AbaP_APK37 and AbTP3phi1, it can be assumed that AbTP3phi1_gp48 is also capable of specifically recognizing K37 CPS.

The CPS-recognizing/degrading part of the TSP encoded by the *A. pittii* phage vB_Api_3043-K38 (QYC50642) shares an average level of similarity with the TSP of *Acinetobacter* phage 3042-K38 (WDS50273) (only gene encoding TSP of this phage was deposited by the authors of annotation in Genbank).

The TSP encoded by the phage vB_AbaP_APK44 (QGK90444), which has an established mechanism of activity toward K44 CPS of *A. baumannii* NIPH70 [31], is highly similar (identity of 96.81%) to the TSP of the phage F70-K44 (WDS49595) isolated on the same bacterial host.

The TSP vB_AbaP_APK48_gp43 (QFG06960), which has a determined K48 CPS substrate specificity [31], is homologous to the TSP of the phage vB_AbaP_APK48-3 (QGH71569) isolated on a bacterial host belonging to the same K48 capsular type. The CPS-recognizing/degrading part of the protein also shares amino acid similarity with the protein encoded in the genome of Obolenskvirus YMC-13-01-C62 (AID17959), which is identical to the proteins encoded by phages P115 (WNT46052), YMC11/12/R2315 (AJT61314), YMC11/12/R1215 (AJT61417), and A832.1 (WNT46469).

The bacterial host of phage APK77 is *A. baumannii* APEX 104 carrying KL77 (unpublished data). The TSP encoded by the phage (UAW09916) is highly similar to the TSP of another Friunavirus fBenAci001 (QOV07748, the coverage obtained to an E-value of 0 was 100% with an identity of 97.62%). APK77_gp50 also shares similarity with the protein encoded by Friunavirus vB_AbaP_PD-AB9 (ALM01895, the coverage obtained to an E-value of 0 was 99% with an identity of 57.60%). The CPS-recognizing/degrading part of APK77_gp50 shares an average level of similarity with the corresponding parts of the proteins encoded by phages Ab31 (WMC00262) and AbP2 (ASJ78888), which are assigned to the genus *Obolenskvirus*, and the protein encoded by the phage SH-Ab 15599 (AXF41546), which belongs to *Ackermannviridae*.

The TSPs APK86_gp49 (UAW09972) and APK87_gp48 (QGK90498), with established mechanisms of action [31,34], are almost identical to each other (identity of 98.89%). Thus, these tailspike depolymerases are specific to both K86 and K87 CPSs [34].

The TSP vB_AbaP_APK89_gp46 (QGK90394), which degrades *A. baumannii* K89 CPS [31], does not share a high level of similarity with any of the lytic phage depolymerases deposited in GenBank. The closest homolog for this depolymerase is the TSP of Friunavirus vB_AbaP_IME546 (QFR59034, the coverage obtained to an E-value of 2 × 10^−166^ was 83% with an identity of 44.60%), which, in this work, was predicted to have K7 specificity.

The CPS-recognizing/degrading part of APK127v_gp47, which has an established mechanism of action toward K127 *A. baumannii* CPS [34], shares only an average level of similarity with the corresponding parts of the proteins encoded by Obolenskviruses P1068 (WHB31253, the coverage obtained to an E-value of 1 × 10^−168^ was 76% with an identity of 53.31%), vB_AbaM_IME512 (AYP69084, the coverage obtained to an E-value of 2 × 10^−168^ was 76% with an identity of 54.01%), WCHABP1 (ARQ94726, the coverage obtained to an E-value of 1 × 10^−160^ was 76% with an identity of 52.33%), and Abp9 (QEA11050, the coverage obtained to an E-value of 1 × 10^−160^ was 76% with an identity of 52.33%).

The TSP of Friunavirus vB_AbaP_APK128 (QVD48888), which degrades K128 CPS [34], shares a high level of similarity at the amino acid level with the protein encoded by the phage phiAB1 (ADQ12745, the coverage obtained to an E-value of 0 was 100% with an identity of 94.65%), indicating the same K specificity of these phages.

The CPS-recognizing/degrading parts of the proteins encoded in the genomes of Friunaviruses Abp1 (AFV51022), AB3 (AGC35305), vB_AbaP_PD-6A3 (ALM01853), SWH-Ab-1 (YP_009949058), vB_AbaP_B09_Aci08 (AYD82867), vB_AbaP_46-62_Aci07 (AYD85862), fBenAci002 (QOV07800), fBenAci003 (QOV07848), vB_Ab4_Hep4 (UVD33039), vB_Ab4_Hep4-M (WIS40047), Acba_6 (WCF71633), vB_ApiP_P1 (ASN73504), vB_ApiP_P2 (ASN73558), vB_Ab-P-7 (WKV23613), and vB_AP_P1489 (WEM05711) do not share significant similarity with any of the lytic phage tailspike enzymes with established or predicted K specificity. In addition, there are no deposited genome sequences of their bacterial hosts to identify the K locus and determine the K type number to which a host strain belongs. Among them, the proteins encoded in the genomes of Friunaviruses vB_AbaP_PD-6A3 (ALM01853) and SWH-Ab-1 (YP_009949058) are almost identical to each other (the coverage obtained to an E-value of 0 was 100% with an identity of 98.77%); the TSP of the *A. baumannii* phage Acba_6 (WCF71633) and the protein encoded by *A. pittii* phage vB_ApiP_P1 are also almost identical (ASN73504, the coverage obtained to an E-value of 0 was 100% with an identity of 97.46%). Thus, these proteins most likely interact specifically with CPSs of the same structure.

The tailspike deacetylase Aristophanes_gp41 (QNO11465) and tailspike depolymerase vB_AbaP_Acibel007_gp46 share no significant similarity with any of the lytic phage tailspike protein sequences deposited in Genbank. According to the HHpred analysis, the C-terminal part of Aristophanes_gp41 shares structural similarity with the GDSL-like lipase/acylhydrolase family protein of *Neisseria meningitidis* (PDB ID: 4K7J, HHpred probability 99.35%).

**Figure 5:**
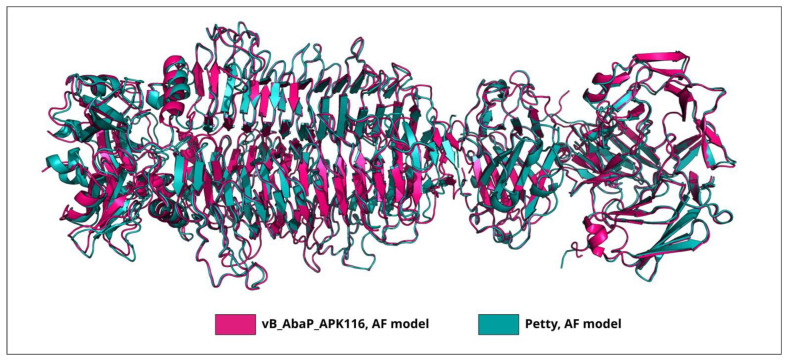
Superimposition of the AF-predicted structures of CPS-recognizing/degrading parts of trimeric TSPs of phages vB_AbaP_APK116 and Petty. The RMSD calculated as an average across subdomains of the CPS-recognizing/degrading parts was 0.65 Å.

The closest homolog for the protein encoded in the genome of Pettyvirus Petty (AGY48011) is the TSP of Friunavirus vB_AbaP_APK116 (QHS01530, the coverage obtained to an E-value of 0 was 87% with an identity of 73.29%). Considering that the CPS-recognizing/degrading parts of these proteins are highly similar (Figure 5), it can be assumed that they specifically interact with *Acinetobacter* K116 CPS.

**Figure 6:**
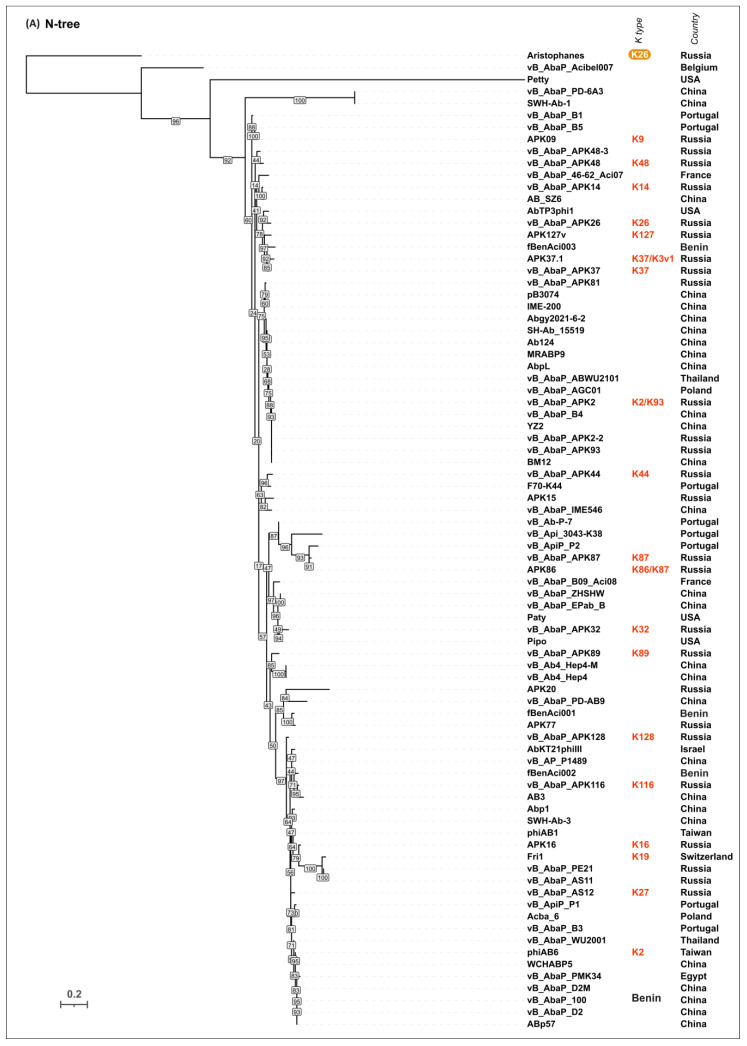

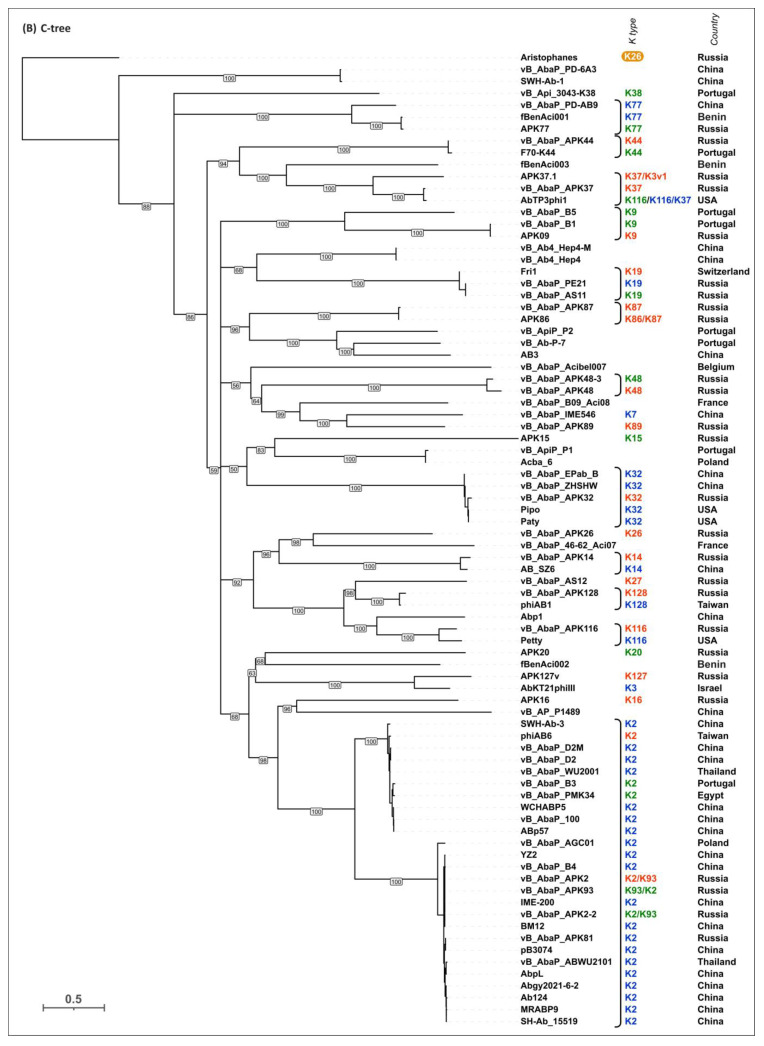

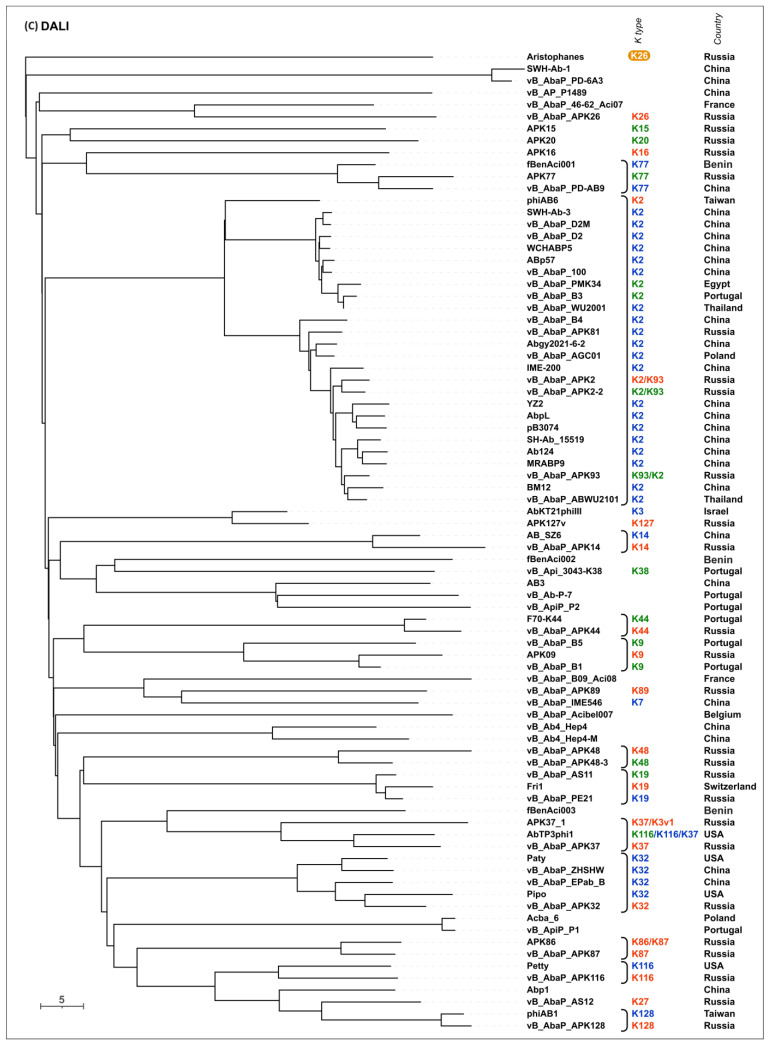
(**A**) Maximum likelihood phylogenetic tree based on the amino acid sequences of the *N-*terminal parts of TSPs of cluster 2 phages. Bootstrap values are shown near their branches. The scale bar shows 0.2 estimated substitutions per site. Monophyletic branches containing phages with the same experimentally derived or predicted K specificity are colored according to the legend. (**B**) Maximum likelihood phylogenetic tree based on the amino acid sequences of the CPS-recognizing/degrading parts of TSPs of cluster 2 phages. Bootstrap values are shown near their branches. The scale bar shows 0.5 estimated substitutions per site. The brackets indicate monophyletic branches containing phages of the same specificity toward a particular *Acinetobacter* K type. The branches with less than 50% support were collapsed. (**C**) Phylogenetic tree based on the DALI structural similarity of the TSPs of cluster 2 phages. The scale bar indicates the DALI Z-score. All trees were rooted to the phage Aristophanes. The experimentally derived K specificity (red), the K specificity predicted by the authors of a phage (green), and the phage specificity predicted in this work (blue) toward a particular Acinetobacter K type are shown in the labels to the right of the phage names (see Table 2). The deacetylating activity of the phage Aristophanes toward the CPS of K26 structure is indicated to the right of the phage name on an orange background. The country of the phage genome depositor is indicated in the right column.

To evaluate the possible evolutionary relationships in the *Beijerinckvirinae* phage TSP formation and to confirm phage K specificity prediction, a phylogenetic analysis was performed. The positions of the conservative *N-*terminal domains and CPS-recognizing/degrading parts of TSPs were determined by comparisons of their amino acid sequences in MAFFT alignments, an analysis of the results of the HHpred searches, and comparisons of the AF-predicted structures. These data were subsequently used to construct the phylogenetic “N-tree” and “C-tree”, respectively. The performed analyses resulted in different topologies of the obtained trees (Figure 6A,B). This can be explained by the characteristic evolutionary history of these phage RBPs, which involves the exchange of genetic modules encoding different TSP domains [13]. The analysis of the topology of phylogenetic trees provides evidence of a similar evolutionary history of the phage MCP and the *N-*terminal part of tailspikes and reveals that the CPS-recognizing/degrading parts of TSPs are more susceptible to horizontal transfer. The phylogenetic analysis based on the structural similarity of *Beijerinckvirinae* TSPs, assessed by DALI [42,46], resulted in a tree the topology of which was closer to that of the tree based on the CPS-recognizing/degrading parts of TSPs than to the topology of the tree based on the *N-*terminal parts of TSPs (Figure 6C). This may be due to the more significant contribution of the CPS-recognizing/degrading parts in the structure comparisons because of their larger size and more diverse structure.

According to the results of the phylogenetic analysis, the capsular specificity of tailspike enzymes is often common for phages that comprise monophyletic groups. This is true to varying degrees for all phylogenetic trees, but for the trees based on the sequences of the CPS-recognizing/degrading part and overall structural similarity, it is more pronounced. For example, the N-tree places the K2-specific phages in two distinct clades, whereas the C-tree clusters all these phages together in a distinct clade composed of two subclades. Additionally, higher branch lengths and statistical support values make the C-tree preferable for K type specificity predictions. The criteria for such predictions would be the monophyletic city of the respective groups and the genetic distances common to the experimentally confirmed cases.

In some cases, the CPS-recognizing/degrading parts of TSPs specific to the CPSs produced by different *Acinetobacter* K types belonged to the same monophyletic groups, e.g., K3- and K127-specific TSPs, K7- and K89-specific TSPs, or K128- and K27-specific TSPs. At the amino acid level, these proteins share an average level of similarity. These enzymes may have a common origin, but in the process of interaction with surface structures, they diverged, which is expressed in a change in their putative active site regions. However, this assumption requires experimental confirmation using structural analysis.

#### 3.4.2. Specificity of the Lytic *Drulisvirus* Phage vB_AbaA_LLY (Cluster 3)

To date, vB_AbaA_LLY is the only *Acinetobacter* phage that has been included in the genus *Drulisvirus*, the subfamily *Slopekvirinae*, and the family *Autographiviridae*. The genus primarily comprises bacterial viruses that infect *Klebsiella* spp. The description of the vB_AbaA_LLY genome sequence contains information that the phage bacterial host is *A. baumannii*. According to the bioinformatic analysis performed in this work, it was predicted that the TSP of this phage corresponds to gp13 (WEV89148), which was annotated by the authors of the sequence as a hypothetical protein. The gene encoding TSP is located immediately after the gene encoding endolysin in the genome of the phage vB_AbaA_LLY. The BLASTp analysis revealed that vB_AbaA_LLY_ gp13 is highly similar to the TSPs encoded by K1-specific *Klebsiella pneumoniae* phages BUCT631 (WAK45693) [145], KpV41 (ALO80745) [146], NTUH-K2044-K1-1 (BAP15746) [147] (identity more than 96%), and other *Klebsiella* phages. Thus, the primary receptor for this phage is the CPS produced by *K. pneumoniae*, which belongs to the K1 type. In addition, the overall genomic architecture and gene homology analyses showed that the phage vB_AbaA_LLY is very similar to a large number of *Klebsiella* phages assigned to the genus *Drulisvirus*.

Therefore, there is a reason to assume that the identification of the phage bacterial host requires further clarification, and vB_AbaA_LLY is, likely, the K1-specific *Klebsiella* phage. However, this assumption requires further experimental confirmation.

#### 3.4.3. Specificity of Lytic *Ackermannviridae* phages (Cluster 6) toward Different *Acinetobacter* K Types

Members of the family *Ackermannviridae* are characterized by a myovirus morphology and a branched receptor-binding protein complex. These phages can encode several TSPs, each of which recognizes and interacts with a specific receptor of their bacterial hosts [78,148].

To date, among all capsule-specific *Acinetobacter* phages with genomes deposited in the NCBI database, there are only two representatives of the family *Ackermannviridae*, phages SH-Ab 15599 and nACB2 (Table 3). Both phages encode three distinct TSPs, which most likely interact with different CPSs [78].

The phages SH-Ab 15599 and nACB2 were isolated on *A. baumannii* 15599 and *A. halotolerans* ANC 5766^T^, respectively [64,78,80]. The capsular types to which these strains belong are unknown. The CPSs produced by the bacterial host strains are substrates for one of the three depolymerases encoded by the phages.

**Figure 7:**
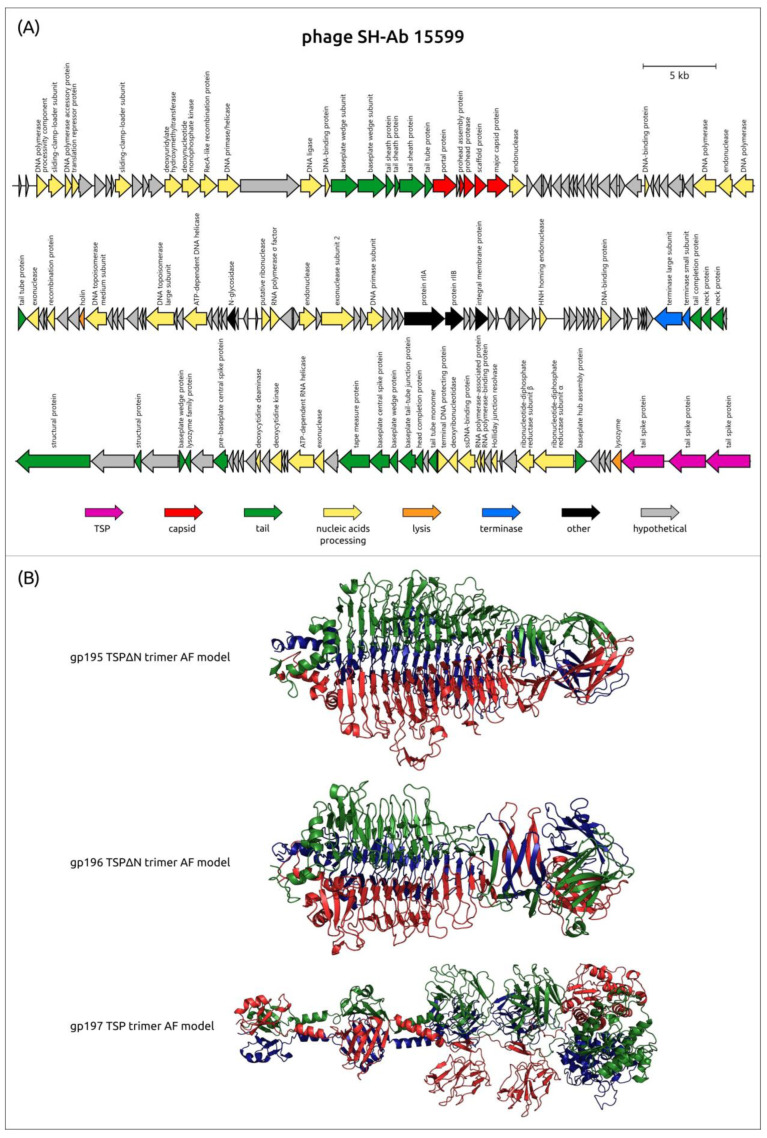
(**A**) Genetic map of *A. baumannii* phage SH-Ab 15599 (Genbank accession number: MH517022). Arrows indicate the direction of transcription. The scale bar indicates the length of the nucleotide sequence. Gene functions are shown in labels and legends. (**B**) Ribbon presentation of the AF-predicted structures of putative tailspikes of *Acinetobacter* phage SH-Ab 15599, where three monomers are colored red, green, and blue. From top to bottom: CPS-recognizing/degrading part of trimeric gp195 (gp195TSP∆N trimer), CPS-recognizing/degrading part of trimeric gp196 (gp196TSP∆N trimer), and trimeric gp197.

Using phage SH-Ab 15599 as an example, AF modeling showed a noticeable difference in the predicted structures of the CPS-recognizing/degrading parts of three different tailspikes encoded in the genome of this phage (Figure 7). The CPS-recognizing/degrading part of the first depolymerase of the phage SH-Ab 15599 (gp195, AXF41546) shares similarity with the TSP of APK77 (UAW09916), which infects *A. baumannii* with the K77 CPS structure. The CPS-recognizing/degrading part of the second depolymerase (gp196, AXF41547) is highly similar to the TSP protein of the phage phiAB6 (ALA12264), which has an established mechanism of enzymatic activity toward K2 CPS [27]. The third protein (gp197, AXF41548) shares no similarity with any of the phage depolymerase sequences deposited in Genbank. The *N-*terminal domain of this protein (the first 314 aa) shares some percentage of identity with the protein encoded in the genome of another *Ackermannviridae* phage, nACB2, which was annotated by the authors as a putative tail with lipase activity (WAW11690). According to the BLASTp analysis, the CPS-recognizing/degrading part of SH-Ab 15599_gp197 shares a high level of similarity with GDSL-type esterase/lipase family proteins encoded in the genomes of different representatives of the genus *Acinetobacter*.

The phage nACB2 encodes three TSPs (gp164–166), two of which were predicted to have capsular depolymerase activities (gp164–165) and one of which (gp166) was presumed to have esterase activity [78]. The CPS-recognizing/degrading parts of the proteins share no similarity with any of the phage depolymerase sequences deposited in Genbank.

#### 3.4.4. Specificity of *Obolenskvirus* Phages (Cluster 8) for Different *Acinetobacter* K Types

Cluster 8 encompasses 39 *Acinetobacter* phages belonging to the genus *Obolenskvirus* and related phages, the genomes of which were deposited in NCBI GenBank by January 2024. The genomes of these phages are characterized by a similar structural architecture and the presence of comparatively large modules of capsid and tail genes. The adsorption apparatus of Obolenskviruses is represented by tail fiber and tailspike proteins encoded by genes located at the end of the genome’s structural module upstream of the genes responsible for proteins associated with bacterial cell lysis [88,91,94].

To date, the mechanisms of the enzymatic activity of only two representatives of the genus *Obolenskvirus* have been determined: the K82-specific phage Scipio [88] and K91(40)-specific phage AP22 [87,126]. The TSP Scipio_gp39 (UQS93268) is a glycosidase, which cleaves the *A. baumannii* K82 CPS by a hydrolytic mechanism [88]. The TSP AP22_gp54 (CCH57762) is a polysaccharide lyase that cleaves the CPS from *A. baumannii* 1053 by *β*-elimination in one of the ManNAcA residues of the K91(40) CPS [126]. The atomic structure of the TSP AP22_gp54, which lacks the full-sized *N-*terminal particle-binding part (PDB ID: 4Y9V), was established.

**Figure 8:**
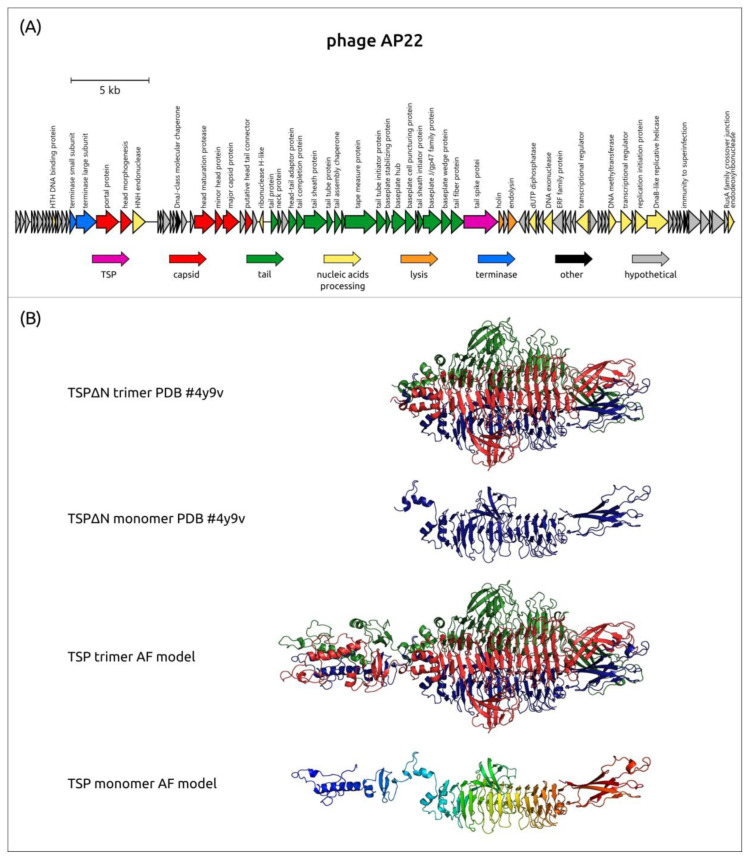
(**A**) Genetic map of *A. baumannii* phage AP22 (official name *Obolenskvirus AP22*, Genbank accession number: HE806280). Arrows indicate the direction of transcription. The scale bar indicates the length of the nucleotide sequence. Gene functions are shown in the labels and legends. (**B**) Experimentally obtained structure of the phage AP22_gp54 TSP trimer and AF-predicted structure model, where the three monomers are colored red, green, and blue. In the figure, from top to bottom: ribbon presentation of the experimentally determined structure of CPS-recognizing/degrading parts of AP22_gp54 TSP trimer (TSP∆N trimer PDB#4Y9V), ribbon presentation of the corresponding blue monomer (TSP∆N monomer PDB#4Y9V), ribbon presentation of the predicted structure of TSP trimer (TSP trimer AF model), and ribbon presentation of TSP monomer (TSP monomer AF model) colored based on a rainbow gradient scheme, where the *N-*terminus of the polypeptide chain is colored blue and the C-terminus is colored red.

The experimentally derived and predicted structures of *Obolenskvirus* TSPs repeat the general feature of the structural architecture of *Friunavirus* tailspikes, where the shorter *N-*terminal part is more conserved than the remaining CPS-recognizing/degrading part. The MAFFT alignment of the 39 amino acid sequences of analyzed phage TSPs gives 85.4% PI between their *N-*terminal parts (corresponding to 1–125 aa residues of AP22 TSP), and the PI between the remaining parts of TSPs is 10.0%. The alignment indicated that the *N-*terminal parts of TSPs of Obolenskviruses are more similar than those of Friunaviruses. The genetic map of the phage AP22 and the experimentally obtained (PDB ID: 4Y9V) and AF-predicted structures of its tailspike lyase are shown in Figure 8.

In Table 4, data on all capsule-specific phages assigned to the genus *Obolenskvirus* and the related phage phiAC-1 with genomes deposited in the NCBI database are summarized in the same manner as in Table 2.

The K2 specificity of *A. baumannii* phages Abp95, vB_AbaM_fThrA, and NJ02 was predicted in this work based on a high percentage of similarity of the CPS-recognizing/degrading parts of the proteins encoded in their genomes (QYC51728, WVH13570, and WJZ47808, respectively) to the sequence of TSPs phiAB6_gp40 and vB_AbaP_APK2_gp43, which have established mechanisms of enzymatic activity toward K2 CPSs [27,31].

The proteins encoded in the genomes of Obolenskviruses WCHABP1 (ARQ94726) and Abp9 (QEA11050) are identical to each other and highly similar to the proteins encoded by phages P1068 (WHB31253, the coverages obtained to an E-value of 0 was 100% with identity of 92.27%) and vB_AbaM_IME512 (AYP69084, the coverages obtained to an E-value of 0 was 100% with identity of 89.12%). As mentioned above, the CPS-recognizing/degrading parts of these proteins share a high percentage of similarity with the sequence, predicted in this work, corresponding to the TSP of the phage AbKT21phiIII, which infects the *A. baumannii* host strain carrying the KL3 locus. Thus, all of these phages are also presumed to be K3-specific.

The bacterial strains susceptible to Obolenskvirus vB_AbaM_IME285 were assigned by the authors to the K9 capsular type [94]. The TSP encoded by this phage (AYP68900) was highly similar to the proteins of other Obolenskviruses, namely WCHABP12 (ARB06757), HZY2308 (WPH63970), and BUCT629 (QZI85319). The parts of these proteins without N-terminal domains were also highly similar to the CPS-recognizing/degrading parts of A. baumannii phages AM24 (APD20249) and BS46 (QEP53229), which have a determined substrate specificity toward K9 CPSs [71,126], and of Friunavirus vB_AbaP_B5 (ASN73455), infecting A. baumannii NIPH 528, which belongs to the K9 type [26].

The closest homologs of the protein encoded in the *Acinetobacter* phage Arbor (URY98759) without the *N-*terminal part were the proteins encoded in *Acinetobacter* phages YMC13/03/R2096 (AIW02768) and P577 (WNT46259) and the TSP of Friunavirus vB_AbaP_APK14 (AYR04394), which have a determined substrate specificity to the CPS of the K14 structure [34].

The proteins encoded in the genomes of *Acinetobacter* phages P115 (WNT46052), YMC-13-01-C62 (AID17959), YMC11/12/R2315 (AJT61314), YMC11/12/R1215 (AJT61417), and A832.1 (WNT46469) are identical. The CPS-recognizing/degrading parts of all these proteins share some percentage of similarity with tailspike proteins of Friunaviruses vB_AbaP_APK48-3 (QGH71569) and vB_AbaP_APK48 (QFG06960), which specifically interact with *A. baumannii* K48 CPS [31]. In this work, the draft assembly of the genome of *A. baumannii* B115, a bacterial host for phage P115, was performed using reads (SRR24880467) deposited by the authors of [82] in the NCBI Sequence Read Archive (https://www.ncbi.nlm.nih.gov/sra, accessed on 22 January 2024). Using Kaptive [22,48], the obtained draft genome sequence was found to include the KL48 locus. Thus, the K48 specificity of the phage P115 and phages YMC-13-01-C62, YMC11/12/R2315, YMC11/12/R1215, and A832.1 encoding identical TSPs are confirmed by the similarity of their TSPs to the proteins with a described substrate specificity and by the prediction of KL48 in the genome of the phage P115 bacterial host.

The proteins of *Acinetobacter* phages P711 (WNT46303) and A2.1 (WNT46385) are identical to each other and almost identical to the proteins encoded in the genomes of Obolenskviruses Bphi-R2919 (QGH74055) and Bphi-R1888 (QGH74134) (identity more than 99%). In this work, the draft genome assembly of *A. baumannii* B711, a bacterial host for phage P711, was obtained using reads (SRR24880838) deposited by the authors of [82]. Using Kaptive, this assembly was found to contain the KL72 locus. Therefore, phages P711, A2.1, Bphi-R2919, and Bphi-R1888 were predicted to infect *A. baumannii* with the K72 CPS structure.

The protein encoded by Obolenskvirus AbP2 (ASJ78888) was highly similar to the proteins of *Acinetobacter* phages Ab31 (WMC00262), Ab59 (WMC00561), and Ab65 (WMC00590 and WMC00591) (identity of 93.43%). The CPS-recognizing/degrading parts of the TSPs of these phages share an average level of similarity with the protein encoded by the phage SH-Ab 15599 (AXF41546), which is assigned to the family *Ackermannviridae*, and the TSP of Friunavirus APK77 (UAW09916), which infects *A. baumannii* with a K77 CPS structure.

The CPS-recognizing/degrading parts of TSPs Scipio_gp39 (UQS93268) and AP22_gp54 (CCH57762), which have a substrate specificity toward *A. baumannii* K82 [88] and K91(40)CPSs [126], respectively, have no significant similarity with any of the lytic phage depolymerase sequences deposited in Genbank.

The TSP Cato_gp43 (UMO77867) was shown to degrade *A. baumannii* K102 CPS (the mechanism of enzymatic activity will be published elsewhere). The protein without the *N-*terminal part only shares similarity with the second of the two depolymerases encoded in the genome of the *A. baumannii* phage TaPaz (tailspike protein II, QVW53860).

The bacterial host for Obolenskvirus Brutus is *A. baumannii* MAR15-3273, which is assigned to the K116 capsular type [88]. The TSP Brutus_gp46 encoded by the phage is highly similar to the protein encoded in the genome of another Obolenskvirus, vB_AbaM_BP10 (UYL86100, the coverage obtained to an E-value of 0 was 100% with an identity 94.43%). The CPS-recognizing/degrading part of the TSP shares amino acid similarity with the protein encoded in the genome of Friunavirus vB_AbaP_APK116 (QHS01530), which has an established mechanism of enzymatic activity toward K116 CPS [31].

The CPS-recognizing/degrading parts of the proteins encoded in the genomes of Obolenskviruses AB1 (ADO14447), vB_AbaM-IME-AB2 (AFV51555), vB_AbaM_IME284 (AYP68982), LZ35 (AMD43190), BUCT628 (QYC51347), XC1 (WFD61290), and the related phage phiAC-1 (AFU62318), do not share significant similarity with lytic phage tailspike depolymerases which have an established or predicted K specificity. In addition, there are no bacterial host genomic data to predict the encoded KL clusters. Among the proteins listed above, the CPS-recognizing/degrading parts of the TSPs of phages phiAC-1, vB_AbaM_IME284, BUCT628, LZ35, and XC1 share no significant identity with any of the lytic phage depolymerase sequences deposited in GenBank. The CPS-recognizing/degrading part of the TSP encoded in the genome of the phage AB1 (ADO14447) is highly similar to the protein of Friunavirus vB_ApiP_P2 (ASN73558), which infects *A. pittii* of an unidentified K type. The CPS-recognizing/degrading part of the TSP encoded in the genome of the phage vB_AbaM-IME-AB2 shares a high level of similarity with the protein encoded in *Friunavirus* Abp1 (AFV51022) with unknown K specificity. The protein encoded by the phage vB_AbM_WUPSU (UJQ43526) without the C-terminal part shares some similarity with the protein of the phage P115 (WNT46052).

**Figure 9:**
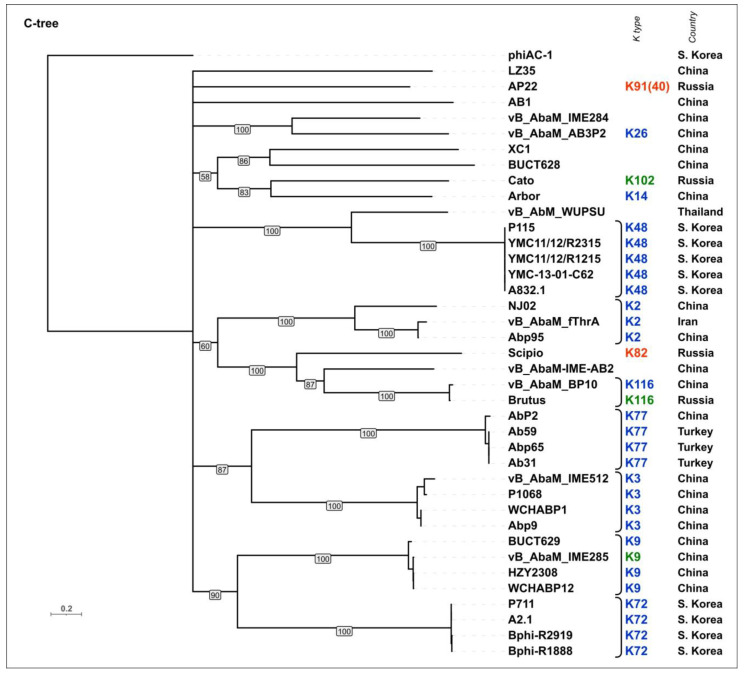
Maximum likelihood phylogenetic tree based on amino acid sequences of the CPS-recognizing/degrading part of tailspike proteins of cluster 8 phages. Bootstrap values are shown near their branches. The scale bar shows 0.2 estimated substitutions per site. The brackets indicate monophyletic branches containing phages of the same specificity toward a particular *Acinetobacter* K type. The branches with less than 50% support collapsed. The trees were rooted to phage phiAC-1. The experimentally derived K specificity (red), the K specificity predicted by the authors of a phage (green), and the phage specificity predicted in this work (blue) toward a particular Acinetobacter K type are shown in the labels to the right of the phage names (see Table 4). The country of the phage genome depositor is indicated in the right column.

A phylogenetic analysis was performed using the amino acid sequences of the conservative *N-*terminal domains (“N-tree”) (Figure S3A), CPS-recognizing/degrading parts of the TSPs (“C-tree”) (Figure 9), and the results of the DALI structural comparison (Figure S3B). Like in *Beijerinckvirinae* phage trees, the N-tree and C-tree do not show identical topologies, but the composition of some clades is similar.

#### 3.4.5. Specificity of the Lytic *Phapecoctavirus* Phage vB_AbaM_ABPW7 (Cluster 10)

Currently, vB_AbaM_ABPW7 is the only *Acinetobacter* phage that has been included in the genus *Phapecoctavirus* of the subfamily *Stephanstirmvirinae*. The genus primarily comprises bacterial viruses that infect *Escherichia* spp. The phage vB_AbaM_ABPW7 was isolated on the multidrug-resistant *A. baumannii* strain ABPW063 and was shown to lyse 45% out of 20 clinical *A. baumannii* strains [100]. The predicted TSP of this phage corresponds to gp174 (UZN23989), which was annotated by the authors of the sequence as a putative colanic acid-degrading protein. According to the BLASTp analysis, the protein is almost identical to colanic acid-degrading proteins encoded by *Escherichia* phages vB_EcoM_ASO2A (UAW58289), iGC_PHA_EC001 (WMI32685), Mt1B1_P17 (QNJ49293) (identity more than 99%), and other phages that were also assigned to the genus *Phapecoctavirus*. The HHpred analysis revealed that the closest structural homolog of vB_AbaM_ABPW7_gp174 is the tailspike colanidase gp150 of *E. coli* phage phi92 (PDB ID: 6E0V). Thus, the primary receptor for this phage is most likely colanic acid. This is a glycopolymer secreted by members of the *Enterobacteriaceae*, including *E. coli* [154,155], which provides protection to bacterial cells against desiccation, oxidative stress, and low pH, and can also be responsible for biofilm formation [156,157,158,159]. As of today, there are no data concerning the production of such polysaccharides by representatives of the genus *Acinetobacter*.

The BLASTn analysis revealed a very high similarity at the DNA level between the phage vB_AbaM_ABPW7, *Escherichia* phage BI-EHEC (OL505078, the coverage obtained to an E-value of 0 was 99% with an identity 98.55%) and other *Escherichia* phages belonging to the genus *Phapecoctavirus*.

On the basis of the above, there is a reason to assume that the phage vB_AbaM_ABPW7 can specifically interact with the members of the *Enterobacteriaceae* which produce colanic acid. Therefore, the aspects of the phage interaction with *A. baumannii* strains and the mechanisms of the recognition of their surface structures require further detailed investigation.

#### 3.4.6. Specificity of the Lytic *Vequintavirinae* Phage ABPH49 (Cluster 12)

**Figure 10:**
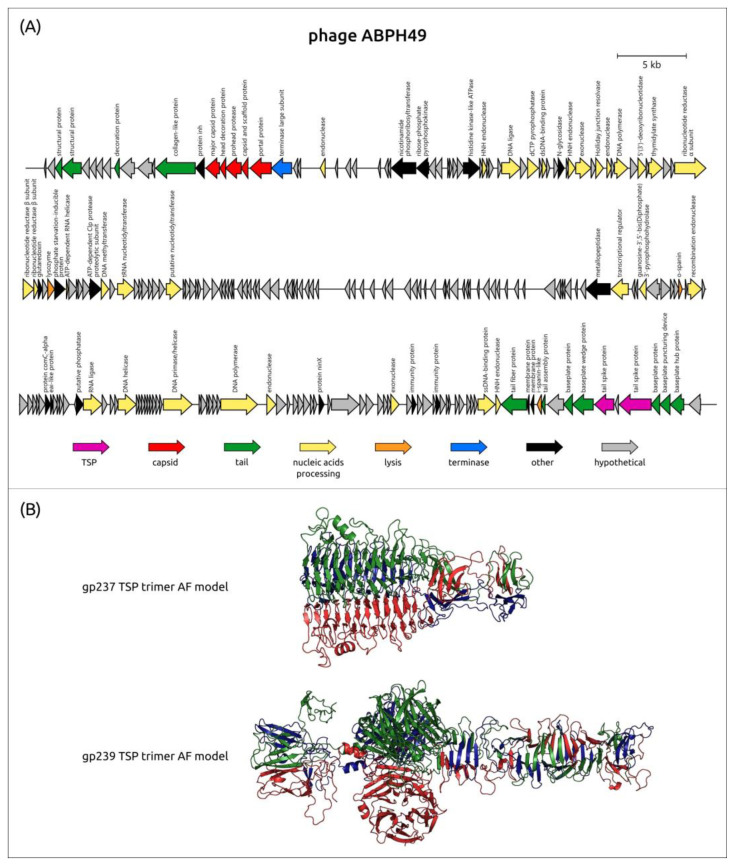
(**A**) Genetic map of *A. baumannii* phage ABPH49 (Genbank accession number: MH533020). Arrows indicate the direction of transcription. The scale bar indicates the length of the nucleotide sequence. Gene functions are shown in the labels and legends. (**B**) Ribbon presentation of the AF-predicted structures of putative trimeric tailspikes ABPH49_gp237 (gp237 TSP trimer AF model) and ABPH49_gp239 (gp239 TSP trimer AF model), where the three monomers are colored red, green, and blue.

A previous genomic analysis of the phage ABPH49 revealed its relatedness to phages of the family *Vequintavirinae* [160]. Interestingly, the MCP phylogenetic tree places the phage ABPH49 in a clade that contains several groups of other myoviruses including phages of clusters 13 and 14 (Figure 1). The bacterial host for this phage was indicated by the authors as *A. baumannii* strain AB49. According to the BLASTn analysis, the closest homolog of the phage ABPH49 is the *Serratia* phage vB_SmaM-Kashira (ON287374, the coverage obtained to an E-value of 0 was 90% with an identity 99.32%), which is assigned to the same family *Vequintavirinae*. The phage ABPH49 genome was predicted to encode two TSPs, located in the tail module, corresponding to gp237 (AXN57968) and gp239 (AXN57970), which were annotated by the authors of the sequence as hypothetical proteins. A BLASTp search indicated the relatedness of ABPH49_gp237 without the *N-*terminal part (without first 146 aa) and ABPH49_gp239 to various tail and tail fiber proteins encoded in the genomes of *Serratia* spp. or in the genomes of phages infecting *Serratia* spp. The HHpred search revealed a similarity between ABPH49_gp237 and the TSP of the *Shigella* phage Sf6 (PDB ID: 2VBK, HHpred probability 99%). The tailspike of the phage Sf6 possesses endorhamnosidase activity and interacts with the *Shigella* cell wall O-antigen [12]. A DALI search using the AF-predicted structure of ABPH49_gp237 (Figure 10) indicated a high level of similarity between this structure and the experimentally determined structures of TSPs and enzymes possessing depolymerizing activity, including the TSP of the *Bacillus* phage phi29 (PDB ID: 3SUC, DALI Z-score 27.6), a bacterial α-1,3-glucanase (PDB ID: 5ZRU, DALI Z-score 24.2), and the putative TSP of the *Bacillus* phage GVE2 (PDB ID: 7CHU, DALI Z-score 23.7). As for ABPH49 gp239, the HHpred search revealed its similarity to sialidases from different cellular organisms (HHpred probability > 99.74%). The DALI search showed a high level of structural similarity between the predicted structure of ABPH49_gp239 (Figure 10) and the experimentally derived structure of the *Escherichia* phage K1F (PDB ID: 3GVJ, DALI Z-score 35.9). The phage K1F specifically recognizes and degrades the polysialic acid (polySia) capsule of *E. coli* using its tailspike endosialidase [161]. The mechanism of the reception and depolymerizing activity of tailspikes of the phage ABPH49 toward *A. baumannii* surface structures needs further investigation and experimental verification.

#### 3.4.7. Specificity of Lytic Unclassified *Caudoviricetes* Phages (Clusters 13 and 14) with Myovirus Morphology for Different *Acinetobacter* K Types

According to the phylogenetic analysis performed using major capsid protein sequences (Figure 1), lytic TSP-carrying *Acinetobacter* phages belonging to unclassified *Caudoviricetes* include two distantly related clusters, conventionally designated as cluster 13 (phages AM24, Bestia, Herod, Mithridates, P577, and YMC13/03/R2096) and cluster 14 (phages BS46, Phab24, TaPaz, and vB_AbaM_B9). Both clusters combine phages with a typical myovirus morphology [28,33,71,111].

**Figure 11:**
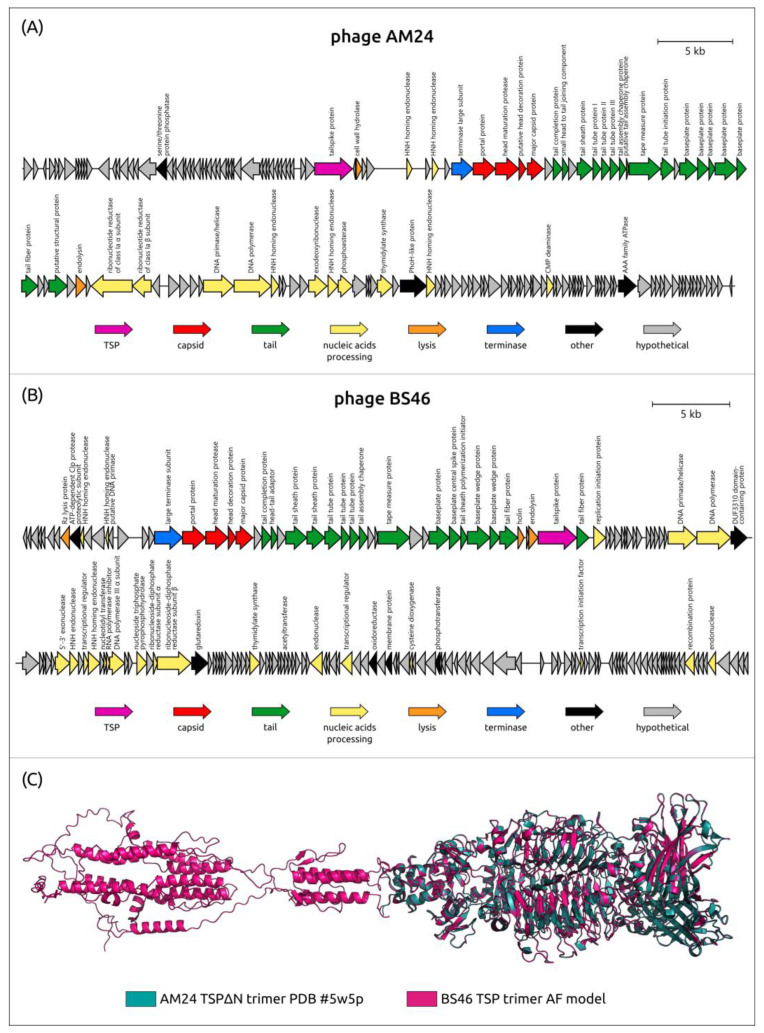
(**A**) Genetic map of *A. baumannii* phage AM24 (Genbank accession number: KY000079). Arrows indicate the direction of transcription. The scale bar indicates the length of the nucleotide sequence. Gene functions are shown in the labels and legends. (**B**) Genetic map of *A. baumannii* phage BS46 (Genbank accession number: MN276049). Arrows indicate the direction of transcription. The scale bar indicates the length of the nucleotide sequence. Gene functions are shown in the labels and legends. (**C**) Superimposition of the AF model of phage BS46 TSP and the experimentally determined structure of the CPS-recognizing/degrading part of the trimeric TSP of phage AM24 (PDB ID: 5W5P) (RMSD 0.24 Å).

Genomes of the phages assigned to clusters 13 and 14 share similarities in general organization and gene content (Figure 11), including the presence of multiple HNH endonuclease genes, which promotes genomic rearrangements. The genomes of all phages assigned to cluster 13 contain only one gene encoding tailspike depolymerase, which is located outside the structural module before the genes responsible for packaging DNA into the capsid [71]. Interestingly, the *N-*terminal conservative region of the cluster 13 phages is longer than that of most *Friunavirus* and *Obolenskvirus* phages, being approximately 250 aa. The phages grouped into cluster 14 also contain only one TSP, except for the phage TaPaz, which encodes two different tailspike depolymerases [33]. Gene encoding TSPs in the genomes of these phages are separated from structural modules by the genes encoding proteins involved in bacterial cell lysis [28,33].

To date, among all the TSPs encoded by the *Acinetobacter* phages of clusters 13 and 14, the atomic structure of the TSP lacking the full-sized *N-*terminal particle-binding part encoded in the genome of the K9-specific phage AM24 (PDB ID: 5W5P) has been established. In addition, within these groups, there are only two proteins with a determined mechanism of cleavage of corresponding CPSs. The first one is BS46_gp47 (QEP53229), which degrades *A. baumannii* K9 CPS [126], the second one is TaPaz_gp78 (QVW53859), which depolymerizes K47 *A. baumannii* CPS [33]. Both depolymerases with established mechanisms of enzymatic activities are specific glycosidases that cleave K9 and K47 CPSs by hydrolytic mechanisms [33,126]. It was shown that the digestion of the K9 CPS from the bacterial host for the phage BS46, *A. baumannii* AC54, by the recombinant protein BS46_gp47, resulted in the formation of a polysaccharide rather than oligosaccharide products. The comparison of the predicted structure of BS46_gp47 and the TSPΔN trimer of phage AM24 indicates a high similarity of these structures (Figure 11). Therefore, the K9 CPS from *A. baumannii* B05, the bacterial host for the phage AM24, which belongs to the same K9 type as *A. baumannii* AC54, was used by the authors as a substrate for the digestion [126]. In Table 5, the data on all capsule-specific *Caudoviricetes* phages with genomes deposited in the NCBI database are summarized in the same manner as in Table 2, which contains data on the *Beijerinckvirinae* phages.

The bacterial host of the phage Mithridates is *A. baumannii* LUH5533, which belongs to the K7 capsular type [144]. The CPS-recognizing/degrading part of the TSP encoded in the genome of the phage Mithridates (QVG63948) only shares a high percentage of identity with the protein encoded by Friuanvirus vB_AbaP_IME546 (QFR59034).

As mentioned above, the CPS-recognizing/degrading part of the TSP AM24_gp50 (APD20249) shares a high level of amino acid similarity with BS46_gp47 (QEP53229), which has an established mechanism of enzymatic activity toward K9 CPS and the TSP encoded in the genome of Friunavirus vB_AbaP_B5 (ASN73455) infecting *A. baumannii* NIPH 528 assigned to the K9 capsular type. The depolymerase was also highly similar to the proteins encoded by Obolenskviruses vB_AbaM_IME285 (AYP68900), which was predicted by the authors of [94] to have K9 specificity, HZY2308 (WPH63970), WCHABP12 (ARB06757), and BUCT629 (QZI85319). The capsule biosynthesis gene cluster identified in the genome of the phage AM24 bacterial host, *A. baumannii* B05 (GenBank accession number MK331712) [71], was found to be identical to the previously described locus of *A. baumannii* RUH134, which was assigned to KL9 (GenBank accession number JN247441.4) [21]. In addition, the purified recombinant protein corresponding to the deletion mutant lacking the *N-*terminal domain of AM24_gp50 forms an opaque halo (zone of CPS depolymerization) on the bacterial lawn of *A. baumannii* B05 [71].

The TSPs encoded in the genomes of phages Herod (QVG64122) and Bestia (QVG64286) share no significant identity with any of the lytic phage tailspike protein sequences deposited in the NCBI Genbank. The bacterial host of the phage Herod is *A. baumannii* KZ-1096, which carries KL10 (unpublished data). The bacterial host for the phage Bestia is *A. baumannii* KZ-1098, the same as that for Friunavirus vB_AbaP_APK26, with an established mechanism of enzymatic activity toward *A. baumannii* K26 CPS [70]. Notably, the CPS-recognizing/degrading parts of the proteins share a fairly low percentage of similarities with each other.

The TSP encoded by the phage YMC13/03/R2096 (AIW02768) is identical to the protein of the phage P577 (WNT46259). The CPS-recognizing/degrading parts of these proteins are highly similar to the TSP encoded in the genome of Obolenskvirus Arbor (URY98759) and homologous to the tailspike depolymerases encoded by Friunaviruses AB_SZ6 (URQ05102) and APK14_gp49 (AYR04394), which have an established enzymatic activity toward K14 CPS [34]. In this work, the draft assembly of the genome of *A. baumannii* B577, a bacterial host for the phage P577, was performed using reads (SRR24880644) deposited by the authors of [82] in the NCBI Sequence Read Archive (https://www.ncbi.nlm.nih.gov/sra, accessed on 22 January 2024). Using Kaptive, the obtained draft genome sequence was found to contain the KL14 locus. Thus, the K14 specificity of the phage P577 is confirmed not only by the similarity of its TSP to the protein with the characterized substrate specificity but also by the identification of KL14 in the genome of the *A. baumannii* bacterial host.

The CPS-recognizing/degrading part of the TSP encoded in the genome of the phage vB_AbaM_B9 (AWD93192) shares no significant identity with any of the lytic phage tailspike protein sequences deposited in Genbank. The authors demonstrated the activity of a recombinant protein corresponding to the C-terminal domain of the TSP vB_AbaM_B9_gp69 on the lawns of *A. baumannii* NIPH 201 and *A. baumannii* NIPH 190, which belong to the K45 and K30 capsular types, respectively, and the activity toward the purified extracted exopolysaccharides obtained from these strains [28].

TaPaz is the only bacterial virus within the group of phages assigned to clusters 13 and 14 that encodes two different complete TSPs. The TSP TaPaz_gp78, with an established mechanism of enzymatic activity toward K47 CPS from *A. baumannii* NIPH 601 [33], shares no significant similarity with the sequences of lytic phage TSPs deposited in Genbank. The CPS-recognizing/degrading part of the TSP TaPaz_gp79 (248–871 aa) only shares similarity with the K102-specific TSP Cato_gp43 (UMO77867) [91], indicating that these proteins are presumed to recognize and degrade CPSs of the same structure. Interestingly, the *N-*terminal part of TaPaz_gp79 was homologous to the hypothetical protein vB_AbaM_B9_gp70 (291 aa, AWD93215) and the putative tail fiber protein BS46_gp48 (256 aa, QEP53230). Thus, these proteins could be incomplete TSPs lacking CPS-recognizing/degrading parts [33].

The TSP encoded in the genome of the phage Phab24 (QXM18609) shares no similarity with any of the phage depolymerase sequences deposited in Genbank. The BLASTp analysis revealed that the protein without the *N-*terminal part was homologous to different sialate *O*-acetylesterases encoded by *Acinetobacter* spp. According to the HHpred analysis, the closest structural homolog to Phab24_gp164 is carbohydrate acetylesterase (PDB ID: 7KMM). Thus, the protein encoded in the genome of the phage Phab24 likely catalyzes the *O*-deacetylation of monosaccharides such as sialic acids by removing the ester decorations and in this way modifies the CPS of the host strain in the process of phage–host interaction.

#### 3.4.8. Specificity of Lytic Unclassified *Caudoviricetes* phages (Clusters 20 and 22) with Siphovirus Morphology

Lytic unclassified *Caudoviricetes* phages with a siphovirus morphology, presumably carrying TSPs, belong to conventionally designated cluster 20 (phages 53, Barton, DMU1, JeffCo, and SH-Ab 15497) and cluster 22 (phage Effie). The genomes of these phages contain one gene, which encodes possible TSP (Table 6).

According to the BLASTp analysis, the TSP Barton_gp20 (QXO06608) shares a high level of similarity with the TSP JeffCo_gp20 (QXO06716, the coverage obtained to an E-value of 0 was 100% with an identity 90.92%) and Effie_gp24 (QXO06658, the coverage obtained to an E-value of 0 was 86% with an identity 88.04%). All the proteins were homologous to SGNH/GDSL-type esterase/lipase family proteins encoded by *Acinetobacter* spp. and other microorganisms. The HHpred search revealed that the closest structural homolog to the proteins is the GDSL-like lipase/acylhydrolase family protein (PDB ID: 4K7J, HHpred probability > 99.9%). The bacterial host of the phages is *A. calcoaceticus* ATCC 23055; thus, the phage RBPs specifically interact with the same surface structures.

The TSP DMU1_gp20 (QOI69765) shares a high level of similarity with the TSP SH-Ab 15497_gp19 (AUG85465, the coverage obtained to an E-value of 0 was 100% with an identity 96.36%) and the predicted TSP of phage 53 (the coverage obtained to an E-value of 0 was 100% with an identity 87.10%). The proteins without the *N-*terminal parts were homologous to different sialate *O*-acetylesterases encoded by *Acinetobacter* spp. According to the HHpred analysis, the closest structural homolog to both proteins is carbohydrate acetylesterase (sialic acid-specific 9-*O*-acetylesterase, PDB ID: 7KMM, HHpred probability >99.9%).

Thus, all phages assigned to cluster 20 and 22 appear to encode tailspike esterases, which catalyze the *O*-deacetylation of monosaccharides in *Acinetobacter* spp. surface carbohydrate structures.

#### 3.4.9. Phylogenetic Analysis of the CPS-Recognizing/Degrading Parts of All TSPs with Established or Predicted K Specificity

**Figure 12:**
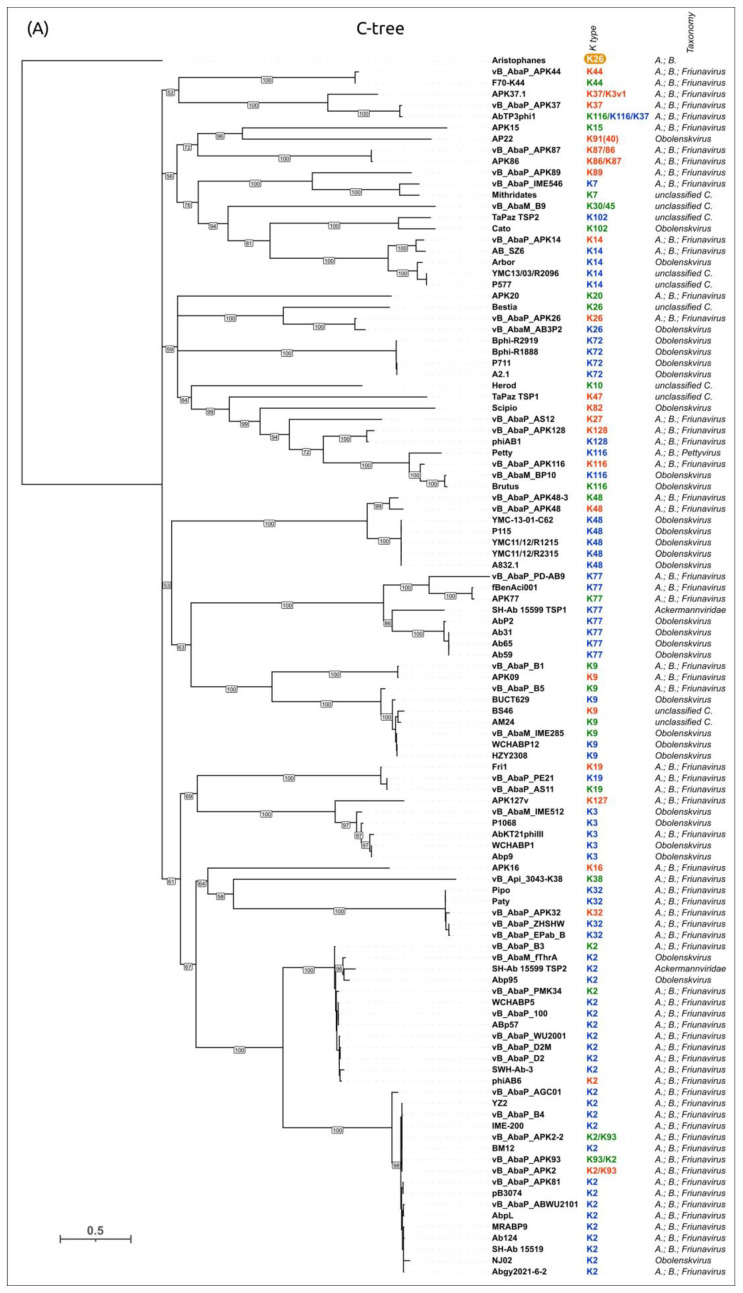

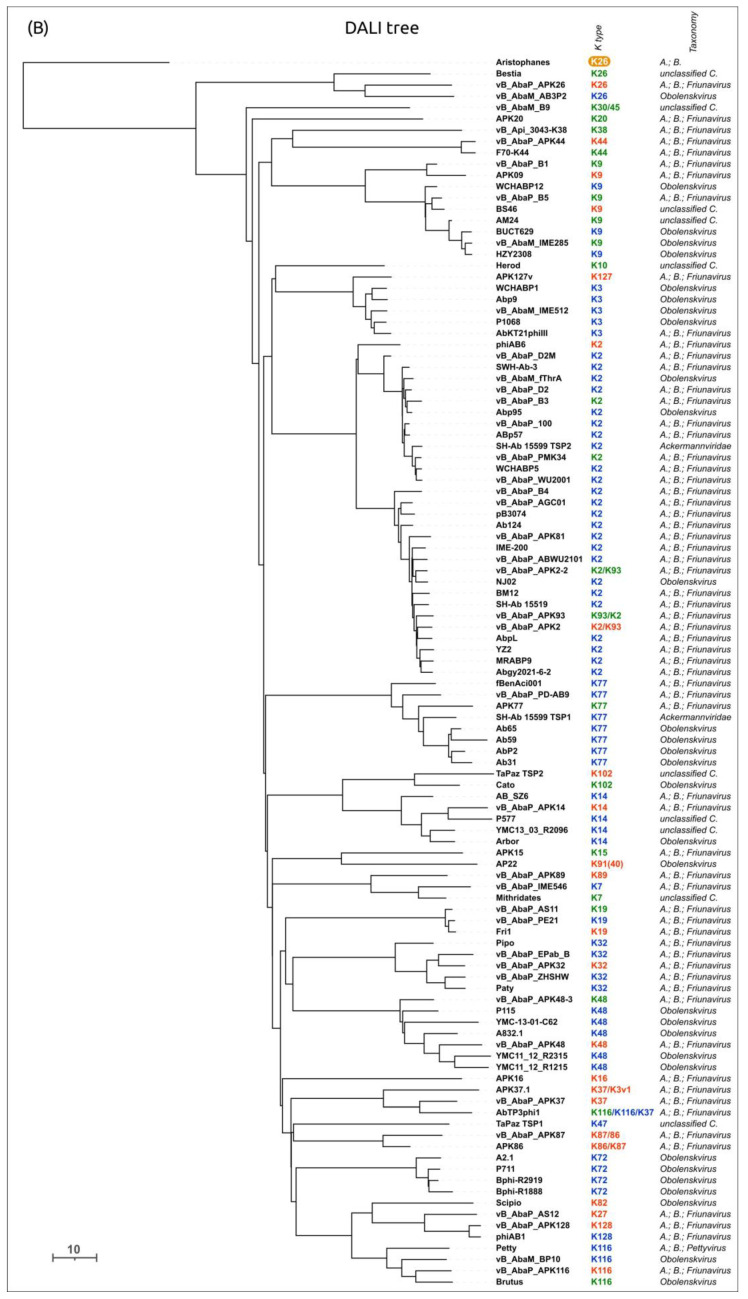
(**A**) Maximum likelihood phylogenetic tree based on the amino acid sequences of the putative CPS-recognizing/degrading parts of the TSP of 109 *Acinetobacter* phages, specifically toward a particular *Acinetobacter* K type. Bootstrap values are shown near their branches. The scale bar shows 0.5 estimated substitutions per site. (**B**) Phylogenetic tree based on the DALI structural similarity of the putative CPS-recognizing/degrading parts of the TSP of 109 *Acinetobacter* phages, specifically toward a particular *Acinetobacter* K type. The scale bar indicates the DALI Z-score. The trees were rooted to the phage Aristophanes. The experimentally derived K specificity (red), the K specificity predicted by the authors of a phage (green), and the phage specificity predicted in this work (blue) toward a particular Acinetobacter K type are shown in the labels to the right of the phage names (see Table 2, Table 3, Table 4 and Table 5), and the experimentally derived deacetylating activity of the phage Aristophanes toward the Acinetobacter K26 type is indicated to the right of the phage name on an orange background. The NCBI taxonomy of the phage is indicated in the right column. The abbreviation “*A.*; *B.*” means “*Autographiviridae*; *Beijerinckvirinae*”, and the abbreviation “*C.*” means “*Caudoviricetes*”.

A phylogenetic analysis based on amino acid sequences (the “C-tree”, Figure 12A) and predicted structures (the “DALI tree”, Figure 12B) was performed using the CPS-recognizing/degrading parts of TSPs with an established or predicted K specificity. These parts of TSPs were extracted from corresponding protein sequences and AF structures using a visual comparison of the predicted TSP monomer models and experimentally determined structures of the CPS-recognizing/degrading parts of *Acinetobacter* phage TSPs. Both the C-tree and the structural tree are similar in clustering together TSPs of the same experimentally derived or predicted specificity toward a particular *Acinetobacter* K type. Importantly, TSPs of the same K specificity were clustered regardless of phage taxonomy, suggesting the possibility of acquiring CPS-recognizing/degrading parts via horizontal transfer.

## 4. Discussion

Among the 233 *Acinetobacter* phages whose genomes were deposited in the NCBI database by January 2024, 143 lytic capsule-specific phages carrying TSPs with established or predicted CPS-depolymerizing/modifying activities were identified. These are phages with a podovirus morphology belonging to the subfamily Beijerinckvirinae (83 phages) and the subfamily Slopekvirinae (one phage) of the family Autographiviridae; phages with a myovirus morphology assigned to the family Ackermannviridae (two phages), the genus Obolenskvirus (39 phages), the subfamily Stephanstirmvirinae, the genus Phapecoctavirus (one phage), the subfamily Vequintavirinae (one phage), and unclassified Caudoviricetes divided into two separate clusters (total, 10 phages); phages with a siphovirus morphology belonging to two different clusters (total, 6 phages). The aspects of the interaction of phages vB_AbaA_LLY (the subfamily *Slopekvirinae*, the genus *Drulisvirus*) and vB_AbaM_ABPW7 (the subfamily *Stephanstirmvirinae*, the genus *Phapecoctavirus*) with *A. baumannii* strains and the mechanisms of the recognition of their surface structures require further detailed investigation, since the RBPs of these phages are almost identical to those of phages that specifically infect *Klebsiella* spp. and *Escherichia* spp., respectively. In the case of the phage ABPH49, two predicted TSPs share an average level of similarity with the tail proteins encoded in the genome of *Serratia* spp. or the phages infecting *Serratia* spp. at the amino acid level. The proteins also share structural similarity with the TSP of the *Shigella* phage Sf6, which possesses endorhamnosidase activity, and the TSP of the *Escherichia* phage K1F, which recognizes and degrades the polySia capsule of *E. coli*. Thus, it is important to study in detail the mechanism of the reception and enzymatic activity of the phage ABPH49 TSPs toward certain *A. baumannii* surface structures.

Most capsule-specific *Acinetobacter* phages encode only one TSP, except for the phage TaPaz (unclassified *Caudoviricetes* with a myovirus morphology included in this work in cluster 14), which carries two complete TSPs with different K specificities, the phage ABPH49 (*Vequintavirinae*), which has two predicted TSPs, and the phages belonging to the family *Ackermannviridae* (SH-Ab 15599 and nACB2), which encode three different TSPs in their genomes. Thus, in total, 149 TSPs were analyzed in this study. Among them, for 24 proteins encoded in the genomes of phages belonging to different taxonomic groups, the mechanisms of enzymatic activity have been established and described [27,30,31,33,34,70,88,126]. The K specificity of 22 TSPs were predicted by the authors in the cited publications [25,26,28,32,71,77,88,91,94,128]. The specificity of 63 TSPs toward different CPSs was predicted in this work using bioinformatic and phylogenetic analyses and AF modeling. For 34 TSPs, it was not possible to determine the specificity toward a CPS of a particular *Acinetobacter* K type using this methodology.

Most TSPs exhibit or are predicted to exhibit CPS-depolymerizing (hydrolase or lyase) activity. The phages carrying these TSPs form plaques with halos that expand with time on the host bacterial lawns. Esterase activity was shown or predicted for TSPs of several phages. These TSPs removed the *O*-acetyl group from specific monosaccharides without the total cleavage of the corresponding CPS. Using the example of the phage Aristophanes encoding the TSP deacetylase, such phages form plaques without visible halos [30].

It is noteworthy that K2-specific phages are the most abundant among all capsule-specific *Acinetobacter* bacterial viruses assigned to different taxonomic groups. In total, 30 phages with an established or predicted K2 specificity were isolated in different countries, suggesting that their bacterial hosts of the K2 type are also widely spread around the world. This is confirmed by the fact that KL2 was found to be the most common K locus, which was identified in 16.5% of 8994 *A. baumannii* genome assemblies [22]. Among the phages belonging to various taxonomic groups isolated in remote geographic locations, K9-, K77-, K3-, K14-, and K48-specific phages are also common. Interestingly, the TSPs of the phages isolated in Taiwan and Russia, which have an established mechanism of enzymatic activity toward K2 CPS, only share a low level of similarity at the amino acid level. The same situation applies to the phages with an established or predicted K9 specificity that were isolated in Russia and Portugal.

The complex phylogenetic analysis carried out in this study revealed the features of the evolution of tailspike proteins of phages, specifically toward a particular *Acinetobacter* K type. Generally, the evolution of enzymes is related to changes in binding specificity to a substrate and alterations in the mechanism of catalysis. In response to the appearance of a new bacterial surface structure, some changes in the phage RBP sequence occur. These changes can include various point mutations, which can result from the RBPs adopting a new surface receptor, or they can be provided by the rearrangements of gene modules or regions and the acquisition of new modules, in particular, because of horizontal gene transfer. In the case of K2- and K9-specific *Acinetobacter* phages, noticeable changes in the amino acid sequence of their TSPs did not deprive them of specificity toward K2 and K9 CPSs, respectively, because of the maintenance of the structural similarity of the proteins. The phylogenetic analysis indicated the possibility of the genetic exchange of gene modules between evolutionarily and morphologically distant groups, such as Friunaviruses, which are characterized by a podovirus morphology, and Obolenskviruses, which are characterized by a myovirus morphology. Importantly, a comprehensive bioinformatics analysis can help reconstruct the evolutionary history of TSPs and identify genetic exchange events, as well as facilitate the informed prediction of phage K specificity, which is important for both fundamental and practical purposes.

## Figures and Tables

**Table 1:** Results of clustering obtained using VIRIDIC intergenomic nucleotide similarity calculations.

Cluster	Phages *	Number of Phages	Taxonomy	Lifestyle **	Morphology
1	Phanie	1	*Astrithrvirus*	lytic	podovirus
2	AB3 (partial genome sequence) [53], Ab124 [54], AB_SZ6 [55], Abgy2021-6-2, AbKT21phiIII [56] ***, Abp1 [57], ABp57, AbpL, AbTP3phi1 [32], Acba_6 [58], AIIMS-AbE5-RC ***, APK09 [34], APK15, APK16 [34], APK20, APK37.1 [34], APK77, APK86 [34], APK127v [34], Aristophanes [30], BM12, F70-K44, fBenAci001 [59], fBenAci002 [59], fBenAci003 [59], Fri1 [25], IME-200 [60], MRABP9, Paty, pB3074 [61], Petty [62,63], phiAB1 [57], phiAB6 [27], Pipo, SH-Ab 15519 [64], SWH-Ab-1, SWH-Ab-3, vB_Ab4_Hep4 [65], vB_Ab4_Hep4-M [65], vB_AbaP_46-62_Aci07 [66], vB_AbaP_100, vB_AbaP_ABWU2101 [67], vB_AbaP_Acibel007 [68], vB_AbaP_AGC01 [69], vB_AbaP_APK2 [26], vB_AbaP_APK2-2, vB_AbaP_APK14 [34], vB_AbaP_APK26 [70], vB_AbaP_APK32 [31], vB_AbaP_APK37 [31], vB_AbaP_APK44 [31], vB_AbaP_APK48 [31], vB_AbaP_APK48-3, vB_AbaP_APK81, vB_AbaP_APK87 [31], vB_AbaP_APK89 [31], vB_AbaP_APK93, vB_AbaP_APK116 [31], vB_AbaP_APK128 [34], vB_AbaP_AS11 [25], vB_AbaP_AS12 [25], vB_AbaP_B1 [26], vB_AbaP_B3 [26], vB_AbaP_B4, vB_AbaP_B5 [71], vB_AbaP_B09_Aci08 [66], vB_AbaP_D2 [72], vB_AbaP_D2M, vB_AbaP_EPab_B, vB_AbaP_IME546, vB_AbaP_PD-6A3 [73], vB_AbaP_PD-AB9 [74], vB_AbaP_PE21, vB_AbaP_PMK34 [75], vB_AbaP_WU2001 [76], vB_AbaP_ZHSHW, vB_Ab-P-7, vB_AP_P1489, vB_Api_3043-K38 [77], vB_ApiP_P1 [26], vB_ApiP_P2 [26], WCHABP5, YZ2	83	*Autographiviridae*; *Beijerinckvirinae*	lytic	podovirus
3	vB_AbaA_LLY	1	*Autographiviridae*; *Slopekvirinae*; *Drulisvirus*	lytic	podovirus
4	nACB1 [78], Presley, VB_ApiP_XC38 [79]	3	*Schitoviridae* ****	lytic	podovirus
5	4316	1	unclassified *Caudoviricetes*	temperate	podovirus
6	nACB2 [78], SH-Ab 15599 [64,80]	2	*Ackermannviridae*	lytic	myovirus
7	vB_AbaM_ME3 [81]	1	*Metrivirus*	lytic	myovirus
8	A2.1, A832.1 [82], AB1 [83], Abp2 [84], Ab31, Ab59, Ab65, Abp9 [85], Abp95 [86], AP22 [87], Arbor, Bphi-R1888, Bphi-R2919, Brutus [88], BUCT628 [89], BUCT629 [90], Cato [91], HZY2308, LZ35, NJ02, P1068, P115 [82], P711 [82], phiAC-1 [92], Scipio [88], vvB_AbaM_AB3P2 [93], vB_AbaM_BP10, vB_AbaM_fThrA, vB_AbaM_IME284, vB_AbaM_IME285 [94], vB_AbaM_IME512, vB_AbaM-IME-AB2 [95], vWUPSU [96], WCHABP1 [97], WCHABP12 [97], XC1, YMC11/12/R1215 [98], YMC11/12/R2315 [98], YMC-13-01-C62	39	*Obolenskvirus* and related phages	lytic	myovirus
9	Ab_121, Abp53 (partial genome sequence) [99], Liucustia, TAC1, vB_AbaM_Acibel004 [68], vB_AbaM_B09_Aci01-1 [66], vB_AbaM_B09_Aci02-2 [66], vB_AbaM_B09_Aci05 [66], vB_AbaM_CP14, vB_AbaM_D22, vB_AbaM_P1, vB_AbaM_phiAbaA1, vB_AbaP_HB01	13	*Saclayvirus*	lytic	myovirus
10	vB_AbaM_ABPW7 [100]	1	*Stephanstirmvirinae*; *Phapecoctavirus*	lytic	myovirus
11	133 [101], AB-Navy1 [32], AB-Navy4 [32], AB-Navy71 [32], AB-Navy97 [32], Abraxas, AbTZA1 [102], AC4 [32], Ac42 [103], Acj9 [103], Acj61 [103], AM101, Henu6, KARL-1, Maestro [32], Melin, Meroveus, Minot, Mokit, Morttis, Octan, PhaR5, Stupor, vB_AbaM_Apostate, vB_AbaM_Berthold, vB_AbaM_DLP1 [104], vB_AbaM_DLP2 [104], vB_AbaM_DP45, vB_AbaM_Kimel, vB_AbaM_Konradin, vB_AbaM_Lazarus, vB_AbaM_PhT2 [105,106], vB_ApiM_fHyAci03 [107], ZZ1 [108]	34	*Straboviridae* *(Tevenvirinae*; *Twarogvirinae* including *Acajnonavirus*, *Hadassahvirus*, *Lasallevirus*, *Lazarusvirus*, and *Zedzedvirus*)	lytic	myovirus
12	ABPH49	1	*Vequintavirinae*	lytic	myovirus
13	AM24 [71], Bestia, Herod, Mithridates, P577 [82], YMC13/03/R2096 [109]	6	unclassified *Caudoviricetes*	lytic	myovirus
14	BS46 [110], Phab24 [111], TaPaz [33], and vB_AbaM_B9 [28]	4	unclassified *Caudoviricetes*	lytic	myovirus
15	Ab105-1phi, AbTJ	2	unclassified *Caudoviricetes*	temperate	myovirus
16	vB_AbaM_ABMM1 [112]	1	unclassified *Caudoviricetes*	temperate	myovirus
17	vB_AbaP_Alexa	1	unclassified *Caudoviricetes*	temperate	myovirus
18	NJ01	1	*Dhillonvirus*	lytic	siphovirus
19	Ab_SZ3 [113], IMEAB3, Loki [114], PBAB25, and vB_AbaS_D0	5	*Lokivirus*	lytic	siphovirus
20	53, Barton, DMU1 [115], JeffCo, SH-Ab 15497 [116]	5	unclassified *Caudoviricetes*	lytic	siphovirus
21	EAb13 [53], vB_AbaS_TCUP2199 [117]	2	unclassified *Caudoviricetes*	lytic	siphovirus
22	Effie	1	unclassified *Caudoviricetes*	lytic	siphovirus
23	Ab1656-2 [118] ***, Ab11510-phi, Acba_1 (partial genome) [58], Acba_3 [58], Acba_4 [58], Acba_11 [58] ***, Acba_13 [58], Acba_14 [58], Acba_15 [58], Acba_16 [58], Acba_18 [58], Aclw_8 [58], Aclw_9 [58], AM106, YC#06, YMC11/11/R3177 [119], YMC/09/02/B1251 [120]	17	*Vieuvirus* and related unclassified phages	temperate	siphovirus
24	5W	1	unclassified *Caudoviricetes*	temperate	siphovirus
25	Ab105-2phi, Ab105-2phideltaCI404ad, and Ab105-3phi	3	unclassified *Caudoviricetes*	temperate	siphovirus
26	fEg-Aba01 [121], fLi-Aba02 [121], and fLi-Aba03 [121]	3	unclassified *Caudoviricetes*	temperate	siphovirus
27	vB_AbaS_TRS1 [122]	1	unclassified *Caudoviricetes*	temperate	siphovirus

* in each cluster, the phage names are listed in alphabetical order, or numerical designations come first, and after them, the alphabetical order of phage names comes; ** the presence of genes encoding integrases in the genomes of *Acinetobacter* phages assigned to clusters 5, 15, 16, 17, 23, 24, 25, 26, and 27 indicates their temperate lifestyle; *** among all *Acinetobacter* phages analyzed, reannotation of the genomic sequences of phages AIIMS-AbE5-RC (Genbank accession number OP291336, cluster 2), AbKT21phiIII (MK278859, cluster 2), Acba_11 (OQ101254, cluster 23), and Ab1656-2 (MZ675741, cluster 23) revealed possible sequencing or assembly errors resulting in the absence or presence of multiple copies of some characteristic *Caudoviricetes* genes; **** despite the low level of intergenomic similarity of *Schitoviridae* phages (5.4–47.9%), they were combined into the same cluster (cluster 4) because of the similar architecture of their tail genomic modules.

**Table 2:** *Beijerinckvirinae* phage specificity toward different *Acinetobacter* K types.

#	Phage Name * (Country of Isolation)	Genbank Accession # (##)	*Acinetobacter* Bacterial Host Strain ** (K Type)	Tailspike Protein			Reference ***	K Specificity of a Phage ****	
				# of Gene Product	Genbank Accession # (##)	Annotation in Genbank			
***Viruses*; *Duplodnaviria*; *Heunggongvirae*; *Uroviricota*; *Caudoviricetes*; *Autographiviridae*; *Beijerinckvirinae*; *Friunavirus***									
1	**phiAB6** **(Taiwan)**	KT339321/ NC_031086	54149 (K2)	gp40	ALA12264/ YP_009288671	tail fiber protein	[27,127]	**K2**	
2	**vB_AbaP_B3** **(Portugal)**	MF033348/ NC_042004	NIPH2061 (K2)	gp42	ASN73401/ YP_009610379	tail fiber protein	[26]	**K2**	
3	**vB_AbaP_PMK34** **(Egypt)**	MN433707	MK34 (N/A); CIP 110467 (K2)	gp45	QGF20174	putative tail fiber	[128]	**K2**	
4	**vB_AbaP_WU2001** **(Thailand)**	MZ099557	ABPW052 (N/A)	gp34	QVQ34730	tail fiber protein	[76]	**K2**	
5	**vB_AbaP_D2** **(China)**	MH042230/ NC_042124	AB9 (N/A)	gp02	AVP40472/ YP_009624618	tail fiber protein	[72]	**K2**	
6	**vB_AbaP_D2M** **(China)**	MN212906	N/A (N/A)	gp06	QFG15400	tail fiber protein	-	**K2**	
7	**WCHABP5** **(China)**	KY888680/ NC_041967	WCHAB1334 (N/A)	gp02	ARQ94869/ YP_009604582	putative tail fiber/tail fiber protein	-	**K2**	
8	**SWH-Ab-3** **(China)**	NC_047883	N/A (N/A)	gp49	YP_009949108	tail fiber protein	-	**K2**	
9	**ABp57** **(China)**	OR578534	N/A (N/A)	gp46	WNV46778	non-contractile tail fiber protein	-	**K2**	
10	vB_AbaP_100 ***** **(China)**	MW926912 (unverified)	N/A (N/A)	the genome sequence was not annotated by the authors; the coordinates of the gene encoding TSP predicted in this work are as follows: 4190–6289			-	**K2**	
11	**vB_AbaP_APK2** **(Russia)**	MK257719	ACICU (K2), 11911 (K93)	gp43	AZU99242	tailspike protein	[31,129,130]	**K2/K93**	
12	**vB_AbaP_APK2-2** **(Russia)**	MK257720	ACICU (K2); 11911(K93)	gp43	AZU99292	tailspike protein	[129,130]	**K2/K93**	
13	**vB_AbaP_APK93** **(Russia)**	MK257721	11911 (K93); ACICU (K2)	gp43	AZU99342	tailspike protein	[129,130]	**K93/K2**	
14	**BM12** **(China)**	OP508218	N/A (N/A)	gp26	UYE92398	tail fiber protein	-	**K2**	
15	**YZ2** **(China)**	OR660046	N/A (N/A)	gp34	WPD49452	tailspike protein	-	**K2**	
16	**vB_AbaP_B4** **(China)**	OR584314	N/A (N/A)	gp16	WNO29457	tail fiber protein	-	**K2**	
17	**IME-200** **(China)**	KT804908/ NC_028987	AB1610 (N/A)	gp48	ALJ97635/ YP_009216489	tail fiber protein	[60]	**K2**	
18	**AbpL** **(China)**	OP171942	AB2 (N/A)	gp45	UVD42134	non-contractile tail fiber protein	-	**K2**	
19	**pB3074** **(China)**	OQ730192	3074 (Bm3074) (N/A)	gp35	WID41884	hypothetical protein	[61]	**K2**	
20	**SH-Ab 15519** **(China)**	KY082667/ NC_041905	15519 (N/A)	gp45	APD19440/ YP_009598268	hypothetical protein/tail fiber protein	[64]	**K2**	
21	**Abgy2021-6-2** **(China)**	OR770644	GY-6 (N/A)	gp47	WPF70339	tail fiber protein	-	**K2**	
22	**Ab124** **(China)**	MT633129	b1928040 (N/A)	gp46	QMP19165	hypothetical protein	[54]	**K2**	
23	**vB_AbaP_ABWU2101** **(Thailand)**	OK546191	ABPW0185 (N/A)	gp8	UFJ83440	tailspike protein	[67]	**K2**	
24	**MRABP9** **(China)**	OP727261	MRAB11 (N/A)	gp41	WAK44760	tailspike protein	-	**K2**	
25	**vB_AbaP_APK81** **(Russia)**	MT741944	36-1512 (N/A)	gp49	QNO11418	tailspike protein	-	**K2**	
26	**vB_AbaP_AGC01** **(Poland)**	MT263719	ATCC^®^16909™ (N/A)	gp48	QIW86364	endolysin	[131]	**K2**	
28	AbKT21phiIII ****** **(Israel)**	MK278859/ NC_048142	*Ab*KT722 (K3) *******	the coordinates of the regions in the genome corresponding to TSP predicted in this work are as follows: 38725–40827 and 262–291			[56]	**K3**	
27	vB_AbaP_IME546 ******** **(China)**	MN061582/ MN395291	N/A (N/A)	gp49/ gp45	QFR59034/ QGJ97530	tail fiber protein	-	**K7**	
29	**APK09** **(Russia)**	MZ868724	B05 (K9)	gp48	UAW09804	tailspike protein	[34]	**K9**	
30	**vB_AbaP_B1** **(Portugal)**	MF033347/ NC_042003	NIPH80 (K9); NIPH528 (K9)	gp45	ASN73353/ YP_00961033	tailspike protein	[26]	**K9**	
31	**vB_AbaP_B5** **(Portugal)**	MF033349/ NC_042005	NIPH80 (K9); NIPH528 (K9)	gp47	ASN73455/ YP_009610433	tailspike protein/tail fiber protein	[26]	**K9**	
32	**vB_AbaP_APK14** **(Russia)**	MK089780	AB5256 (K14)	gp49	AYR04394	tail spike protein, structural depolymerase	[34]	**K14**	
33	**AB_SZ6** **(China)**	ON513429	N/A (N/A)	gp44	URQ05102	tail spike protein	[55]	**K14**	
34	**APK15** **(Russia)**	MZ936315	MAR15-4788 (K15)	gp48	UAW10027	tailspike protein	-	**K15**	
35	**APK16** **(Russia)**	MZ868725	D4 (K16)	gp47	UAW09859	tailspike protein	[34,132]	**K16**	
36	**Fri1** **(Switzerland)**	KR149290/ NC_028848	28 (K19)	gp49	AKQ06854/ YP_009203055	tail spike protein	[25,126,133]	**K19**	
37	**vB_AbaP_AS11** **(Russia)**	KY268296/ NC_041915	28 (K19)	gp45	AQN32697/ YP_009599281	tail spike protein	[25,133]	**K19**	
38	**vB_AbaP_PE21** **(Russia)**	OL964948	28 (K19)	gp45	ULG00671	tail spike protein	[133]	**K19**	
39	**APK20** **(Russia)**	MZ936316	MAR14-595 (K20)	gp52	UAW10085	tailspike protein	-	**K20**	
40	**vB_AbaP_APK26** **(Russia)**	MW345241	KZ-1098 (K26)	gp48	QQO97001	tail spike protein	[70]	**K26**	
41	**vB_AbaP_AS12** **(Russia)**	KY268295/ NC_041914	1432 (K27)	gp42	APW79830/ YP_009599229	tail spike protein/ tail fiber protein	[18,25,126]	**K27**	
42	**vB_AbaP_APK32** **(Russia)**	MK257722	LUH5549 (K32)	gp46	AZU99395	tailspike protein	[31,134]	**K32**	
43	**Pipo** **(USA)**	MW366783	N/A (N/A)	gp52	QQO92973	tailspike protein	-	**K32**	
44	**Paty** **(USA)**	MW366784	N/A (N/A)	gp49	QQM15083	tailspike protein	-	**K32**	
45	**vB_AbaP_ZHSHW** **(China)**	OM925528	8_4 (N/A)	gp45	UPT53561	tailspike protein	-	**K32**	
46	**vB_AbaP_EPab_B** **(China)**	OQ730212	Ab_8_4 (N/A)	gp41	WGV35678	tail spike protein	-	**K32**	
47	**vB_AbaP_APK37** **(Russia)**	MK257723	NIPH146 (K37)	gp44	AZU99445	tailspike protein	[31,135,136]	**K37**	
48	**APK37.1** **(Russia)**	MZ967493	KZ-1101 (K37), AB5001 (K3v1)	gp49	UAW07728	tailspike protein	[34,136]	**K37/K3v1**	
49	**vB_Api_3043-K38** **(Portugal)**	MZ593174	*A. pittii* Ap45 (K38)	gp46	QYC50642	tailspike protein	[77]	**K38**	
50	**vB_AbaP_APK44** **(Russia)**	MN604238	NIPH70 (K44)	gp44	QGK90444	tailspike protein	[18,31]	**K44**	
51	**F70-K44** **(Portugal)**	OQ378314	NIPH 70 (K44)	gp41	WDS49595	tailspike protein	-	**K44**	
52	**vB_AbaP_APK48** **(Russia)**	MN294712	NIPH615 (K48)	gp43	QFG06960	tailspike protein	[17,31]	**K48**	
53	**vB_AbaP_APK48-3** **(Russia)**	MN614471	APEX-294 (K48)	gp48	QGH71569	tailspike protein	-	**K48**	
54	**APK77** **(Russia)**	MZ868726	APEX 104 (K77)	gp50	UAW09916	tailspike protein	-	**K77**	
55	**fBenAci001** **(Benin)**	MW056501	5542 (N/A)	gp44	QOV07748	tailspike protein	[59]	**K77**	
56	**vB_AbaP_PD-AB9** **(China)**	KT388103/ NC_028679	N/A (N/A)	gp07	ALM01895/ YP_009189830	hypothetical protein/tail fiber protein	[74]	**K77**	
57	**APK86** **(Russia)**	MZ936314	MAR55-66 (K86)	gp49	UAW09972	tailspike protein	[34,137]	**K86/K87**	
58	**vB_AbaP_APK87** **(Russia)**	MN604239	LUH5547 (K87)	gp48	QGK90498	tailspike protein	[31,138]	**K87/86**	
59	**vB_AbaP_APK89** **(Russia)**	MN651570	LUH5552 (K89)	gp46	QGK90394	tailspike protein	[31,139]	**K89**	
60	**vB_AbaP_APK116** **(Russia)**	MN807295	MAR303 (K116)	gp43	QHS01530	tailspike protein	[31,140]	**K116**	
61	AbTP3phi1 ********* **(USA)**	OL770263	TP3 (K116)	gp48	UNI74976	tail fiber	[32]	**K116**	**K116/K37**
62	**APK127v** **(Russia)**	ON210142	36-1454 (K127)	gp47	URQ05189	tailspike protein	[19,34]	**K127**	
63	**vB_AbaP_APK128** **(Russia)**	MW459163	KZ-1093 (K128)	gp45	QVD48888	tailspike protein	[34,141]	**K128**	
64	**phiAB1** **(Taiwan)**	HQ186308/ NC_028675	M68316 (N/A)	gp41	ADQ12745/ YP_009189380	tail fiber protein	[142]	**K128**	
65	**Abp1** **(China)**	JX658790/ NC_021316	AB1 (N/A)	gp47	AFV51022/ YP_008058239	hypothetical protein/tail fiber protein	[57]	**-**	
66	**AB3** **(China)**	KC311669/NC_021337 (partial genome sequence)	N/A (N/A)	gp04	AGC35305/ YP_008060136	hypothetical protein/tail fiber protein	[143]	**-**	
67	**vB_AbaP_PD-6A3** **(China)**	KT388102/ NC_028684	Ab32 (N/A)	gp13	ALM01853/ YP_009190472	hypothetical protein/tail fiber protein	[73]	**-**	
68	**SWH-Ab-1** **(China)**	NC_047896	N/A (N/A)	gp47	YP_009949058	tail fiber protein	-	**-**	
69	**vB_AbaP_B09_Aci08** **(France)**	MH763831/ NC_048081	Paris B09 (N/A)	gp46	AYD82867/ YP_009814060	putative capsular polysaccharide depolymerase/tail fiber protein	[66]	**-**	
70	**vB_AbaP_46-62_Aci07** **(France)**	MH800200/ NC_048076	N/A (N/A)	gp45	AYD85862/ YP_009813438	putative capsular polysaccharide depolymerase/ tail fiber protein	[66]	**-**	
71	**fBenAci002** **(Benin)**	MW056502	5707 (N/A)	gp46	QOV07800	tailspike protein	[59]	**-**	
72	**fBenAci003** **(Benin)**	MW056503	5910 (N/A)	gp41	QOV07848	tailspike protein	[59]	**-**	
73	**vB_Ab4_Hep4** **(China)**	OP019135	Ab4 (N/A)	gp43	UVD33039	non-contractile tail fiber protein	[65]	**-**	
74	**vB_Ab4_Hep4-M** **(China)**	OR075895	Ab4 (N/A)	gp34	WIS40047	non-contractile tail fiber protein	[65]	**-**	
75	**Acba_6** **(Poland)**	OQ101251	3940 (N/A)	gp44	WCF71633	tailspike protein	[58]	**-**	
76	**vB_ApiP_P1** **(Portugal)**	MF033350/ NC_042006	*A. pittii* CEB-AP (N/A)	gp43	ASN73504/ YP_009610482	tailspike protein/ tail fiber protein	[26]	**-**	
77	**vB_ApiP_P2** **(Portugal)**	MF033351/ NC_042007	*A. pittii* NIPH 76 (N/A)	gp48	ASN73558/ YP_009610536	tailspike protein/ tail fiber protein	[26]	**-**	
78	**vB_Ab-P-7** **(Portugal)**	OQ982387	N/A (N/A)	gp43	WKV23613	tail spike protein	-	**-**	
79	**vB_AP_P1489** **(China)**	OQ451773	*A. pittii* N/A (N/A)	gp3	WEM05711	non-contractile tail fiber protein	-	**-**	
80	**AIIMS-AbE5-RC** **(India)**	OP291336	N/A (N/A)	there is no region in the genome corresponding to TSP due to possible sequencing or assembly errors				**-**	
***Viruses*; *Duplodnaviria*; *Heunggongvirae*; *Uroviricota*; *Caudoviricetes*; *Autographiviridae*; *Beijerinckvirinae***									
1	**Aristophanes** **(Russia)**	MT783706	KZ-1098 (K26)	gp41	QNO11465	tail spike protein	[30,70]	**K26**	
***Viruses*; *Duplodnaviria*; *Heunggongvirae*; *Uroviricota*; *Caudoviricetes*; *Autographiviridae*; *Beijerinckvirinae*; *Daemvirus*; *Daemvirus acibel007***									
1	**vB_AbaP_Acibel007** **(Belgium)**	KJ473423/NC_025457	070517/0072 (N/A)	gp46	AHY26817/ YP_009103257	putative tail fiber/tail protein	[68]	**-**	
***Viruses*; *Duplodnaviria*; *Heunggongvirae*; *Uroviricota*; *Caudoviricetes*; *Autographiviridae*; *Beijerinckvirinae*; *Pettyvirus*; *Pettyvirus petty***									
1	**Petty** **(USA)**	KF669656/ NC_023570	*A. nosocomialis* AU0783 (N/A)	gp39	AGY48011/ YP_009006536	tail fiber protein	[62,63]	**K116**	

* *Acinetobacter* phages are arranged within the table in order of increasing bacterial host capsular type number; ** this column of the table only shows the strain designation without the species name *A. baumannii*; however, if the phage bacterial host does not belong to the species *A. baumannii*, both the species name and the strain designation are indicated; N/A means not available; *** this table contains reference(s) not only to works devoted to the characterization of bacteriophage(s) and/or phage-encoded depolymerase(s), but also reference(s) to works on the characterization of the capsule type of bacterial host strain(s), if available; **** **red color:** phage specificity toward a particular *Acinetobacter* K type was determined by the authors in the cited articles; the mechanism of the specific cleavage of *Acinetobacter* CPS by a phage-encoded TSP was established and described; **green color:** prediction of phage specificity toward a particular *Acinetobacter* K type was made by the authors in the cited articles; **blue color:** prediction of phage specificity toward a particular *Acinetobacter* K type was made in this study; **without color:** it was not possible to determine phage specificity toward a particular *Acinetobacter* K type because of the lack of significant similarity of sequences of phage-encoded TSPs with any of the phage depolymerase sequences deposited in GenBank or because of the lack of data on phage bacterial hosts; ***** the genome sequence was not annotated by the authors, and the coordinates of the gene encoding TSP predicted in this work are as follows: 4190–6289; ****** the gene encoding TSP was not predicted by the authors of genome annotation; there is an insertion of a sequence in the TSP coding region suggesting genome assembly errors, in addition, the part of the sequence corresponding to the TSP was duplicated in the beginning and the end of the deposited linear genome. The coordinates of the regions in the genome corresponding to TSP predicted in this work are as follows: 38725–40827 and 262–291; ******* KL3 was identified in this study; ******** there are two variants deposited in the Genbank genome sequences of phage vB_AbaP_IME546 with designations “partial” and “complete”. The Genbank accession numbers of both variants are provided in the table; ********* KL116 was identified in the genome of *A. baumannii* TP3 by the authors in the cited article; the possible specificity of phage AbTP3phi1 TSP toward K37 CPS was also predicted.

**Table 3:** Lytic *Ackermannviridae* phage specificity toward the *Acinetobacter* K type.

#	Phage Name (Country)	Genbank Accession # (##)	*Acinetobacter* Bacterial Host(s) (K Type)	Tailspike Protein			Reference	K Specificity of a Phage *
				# of Gene Product	Genbank Accession #	Annotation in Genbank		
1	**SH-Ab 15599** **(China)**	MH517022	*A. baumannii* 15599 (N/A) **	gp195	AXF41546	hypothetical protein	[64,80]	**K77**
				gp196	AXF41547	tail fiber		**K2**
				gp197	AXF41548	hypothetical protein		**-**
2	**nACB2** **(Portugal)**	OQ032512	*Acinetobacter halotolerans* ANC 5766^T^ (N/A)	gp164	WAW11689	tailspike	[78]	**-**
				gp165	WAW11692	tailspike		**-**
				gp166	WAW11690	putative tail with lipase actitity		**-**

* **Blue color:** prediction of phage specificity toward a particular *Acinetobacter* K type was made in this study; **without color:** it was not possible to determine phage specificity toward a particular *Acinetobacter* K type because of the lack of significant similarity of sequences of phage-encoded TSPs with any of the phage depolymerase sequences deposited in the Genbank or because of the lack of data on phage bacterial hosts; ** N/A means not available.

**Table 4:** *Obolenskvirus* phage specificity towards *Acinetobacter* K type.

#	Phage Name * (Country)	Genbank Accession # (##)	*Acinetobacter* Bacterial Host ** (K Type)	Tailspike Protein			Reference ***	K Specificity of a Phage ****
				# of Gene Product	Genbank Accession # (##)	Annotation in Genbank		
1	**Abp95** **(China)**	MZ618622	AB_2013-95_ (N/A)	gp55	QYC51728	pectate lyase superfamily protein	[86]	**K2**
2	**vB_AbaM_fThrA** **(Iran)**	PP171454	Tehran-1 (N/A)	gp50	WVH13570	tail fiber protein	-	**K2**
3	**NJ02** **(China)**	OR126895	N/A (N/A)	gp64	WJZ47808	tail fiber protein	-	**K2**
4	**WCHABP1** **(China)**	KY829116/ NC_041966	Ab1186 (N/A)	gp5	ARQ94726/ YP_009604496	putative tail fiber protein	[97]	**K3**
5	**Abp9** **(China)**	MN166083	AB_ZY_9 (N/A)	gp49	QEA11050	hypothetical protein	[85]	**K3**
6	**vB_AbaM_IME512** **(China)**	MH853788	N/A (N/A)	gp66	AYP69084	hypothetical protein	-	**K3**
7	**P1068** **(China)**	OQ689089	N/A (N/A)	gp30	WHB31253	hypothetical protein	-	**K3**
8	**vB_AbaM_IME285** **(China)**	MH853786	Ab387 (K9)	gp49	AYP68900	tail fiber protein	[94]	**K9**
9	**WCHABP12** **(China)**	KY670595/ NC_041924	Ab1262 (N/A)	gp16	ARB06757/ YP_009600510	tail fiber protein	[97]	**K9**
10	**HZY2308** **(China)**	OR730450	N/A (N/A)	gp55	WPH63970	tail fiber protein	-	**K9**
11	**BUCT629** **(China)**	MZ712044	N/A (N/A)	gp16	QZI85319	tail fiber protein	[90]	**K9**
12	**Arbor** **(China)**	ON237674	XH1383 (N/A)	gp45	URY98759	putative tail fiber protein	-	**K14**
13	**vB_AbaM_AB3P2** **(China)**	OR526523	AB3 (N/A)	gp17	WOZ14994	tail spike protein	[93]	**K26**
14	**P115** **(South Korea)**	OR180306	B115 (K48) *****	gp42	WNT46052	hypothetical protein	[82]	**K48**
15	**YMC-13-01-C62** **(South Korea)**	KJ817802/ NC_024785	YMC/13/01/C62 (N/A)	gp45	AID17959/ YP_009055466	hypothetical protein	-	**K48**
16	**YMC11/12/R2315** **(South Korea)**	KP861229/ NC_028855	YMC11/12/R2315 (N/A)	gp83	AJT61314/ YP_009203602	hypothetical protein	[98]	**K48**
17	**YMC11/12/R1215** **(South Korea)**	KP861231	YMC11/12/R1215 (N/A)	gp21	AJT61417	hypothetical protein	[98]	**K48**
18	**A832.1** **(South Korea)**	OR180310	B115 (K48) *****	gp40	WNT46469	hypothetical protein	[82]	**K48**
19	**P711** **(South Korea)**	OR180308	B711 (K72) ******	gp39	WNT46303	hypothetical protein	[82]	**K72**
20	**Bphi-R2919** **(South Korea)**	MN516421	YMC18/02/R2919 (N/A)	gp20	QGH74055	hypothetical protein	-	**K72**
21	**Bphi-R1888** **(South Korea)**	MN516422	YMC17/03/R1888 (N/A)	gp19	QGH74134	hypothetical protein	-	**K72**
22	**A2.1** **(South Korea)**	OR180309	N/A (N/A)	gp39	WNT46385	hypothetical protein	-	**K72**
23	**AbP2** **(China)**	MF346584/ NC_041998	AB2 (N/A)	gp17	ASJ78888/ YP_009609870	tail fiber protein	[84]	**K77**
24	**Ab31** **(Turkey)**	OR045355	N/A (N/A)	gp55	WMC00262	tail fiber protein	-	**K77**
25	**Ab59** **(Turkey)**	OR045357	N/A (N/A)	gp95	WMC00561	tail fiber protein	-	**K77**
26	**Ab65** **(Turkey)**	OR045358	N/A (N/A)	gp28 and gp29 *******	WMC00590 and WMC00591	tail fiber proteins	-	**K77**
27	**Scipio** **(Russia)**	ON036883	LUH5534 (K82)	gp39	UQS93268	tailspike protein	[88,149]	**K82**
28	**AP22** **(Russia)**	HE806280/ NC_017984	1053 (K91)	gp54	CCH57762/ YP_006383804	hypothetical protein/tail protein	[87,126,150]	**K91 (40)**
29	**Cato** **(Russia)**	OM471864	KZ-1102 (K102)	gp43	UMO77867	tailspike protein	[91]	**K102**
30	**Brutus** **(Russia)**	ON036882	MAR15-3273 (K116)	gp46	UQS93189	tailspike protein	[88]	**K116**
31	**vB_AbaM_BP10** **(China)**	OP585104	AB10 (N/A)	gp74	UYL86100	tail spike protein, capsular polysaccharide depolymerase	-	**K116**
32	**AB1** **(China)**	HM368260/ NC_042028	KD311 (N/A)	gp76	ADO14447/ YP_009613841	tail fiber protein	[83,151]	**-**
33	**phiAC-1** **(South Korea)**	JX560521/ NC_028995	*Acinetobacter soli* KZ-1 (N/A)	gp69	AFU62318/ YP_009216837	putative tail fiber protein	[92]	**-**
34	**vB_AbaM-IME-AB2** **(China)**	JX976549/ NC_041857	MDR-AB2 (N/A)	gp71	AFV51555/ YP_009592222	putative tail fiber	[152]	**-**
35	**vB_AbaM_IME284** **(China)**	MH853787	N/A (N/A)	gp48	AYP68982	hypothetical protein	-	**-**
36	**LZ35** **(China)**	KU510289/ NC_031117	N/A (N/A)	gp30	AMD43190/ YP_009291902	putative tail-fiber protein	[153]	**-**
37	**BUCT628** **(China)**	MZ593728	N/A (N/A)	gp25	QYC51347	hypothetical protein	[89]	**-**
38	**vB_AbM_WUPSU** **or vWUPSU** **(Thailand)**	OL743187	NPRCOE 160519 (N/A)	gp83	UJQ43526	putative tail-fiber protein	[96]	**-**
39	**XC1** **(China)**	OQ547903	*A. nosocomialis* N/A (N/A)	gp83	WFD61290	tail fiber protein	-	**-**

* *Acinetobacter* phages are arranged within the table in order of increasing bacterial host capsular type number; ** this column of the table only shows the strain designation without the species name *A. baumannii*; however, if the phage bacterial host does not belong to the species *A. baumannii*, both the species name and the strain designation are indicated; N/A means not available; *** this table contains reference(s) not only to works devoted to the characterization of bacteriophage(s) and/or phage-encoded depolymerase(s), but also reference(s) to works on the characterization of the capsular type of bacterial host strain(s), if available; **** **red color:** phage specificity toward a particular *Acinetobacter* K type was determined by the authors in the cited articles; the mechanism of the specific cleavage of *Acinetobacter* CPS by a phage-encoded TSP was established and described; **green color:** prediction of phage specificity towards a particular *Acinetobacter* K type was made by the authors in the cited articles; **blue color:** prediction of phage specificity toward a particular *Acinetobacter* K type was made in this study; **without color:** it was not possible to determine phage specificity toward a particular *Acinetobacter* K type because of the lack of significant similarity of sequences of phage-encoded TSPs with any of the phage depolymerase sequences deposited in GenBank or because of the lack of data on phage bacterial hosts; ***** KL48 was identified in this study; ****** KL72 was identified in this study; ******* the sequence encoding TSP of phage Ab65, most likely, contains an error in the assembly and should include a nucleotide consisting of an adenine (“a”) in the position of 11,693 bp (on the top strand); due to this error, TSP is divided into two proteins (gp28 and gp29) annotated by the authors as tail fiber proteins.

**Table 5:** Specificity of lytic unclassified *Caudoviricetes* phages with myovirus morphology toward different *Acinetobacter* K types.

#	Phage Name * (Country)	Genbank Accession # (##)	*A. baumannii* Bacterial Host(s) ** (K Type)	Tailspike Protein			Reference ***	K Specificity of a Phage ****
				# of Gene Product	Genbank Accession # (##)	Annotation in Genbank		
***Viruses*; *Duplodnaviria*; *Heunggongvirae*; *Uroviricota*; *Caudoviricetes* (Cluster 13)**								
1	**Mithridates** **(Russia)**	MW316731	LUH 5533 (K7)	gp61	QVG63948	tail spike protein	[144]	**K7**
2	**AM24** **(Russia)**	KY000079	B05 (K9)	gp50	APD20249	tailspike protein	[71]	**K9**
3	**Herod** **(Russia)**	MW316732	KZ-1096 (K10)	gp58	QVG64122	tail spike protein	-	**K10**
4	**YMC13/03/R2096** **(South Korea)**	KM672662/NC_027332	YMC13/03/R209 (N/A)	gp34	AIW02768/ YP_009146765	tail fiber protein	[109]	**K14**
5	**P577** **(South Korea)**	OR180307	B577 (K14) *****	gp162	WNT46259	putative tail fiber	[82]	**K14**
6	**Bestia** **(Russia)**	MW316733	KZ-1098 (K26)	gp53	QVG64286	tail spike protein	[70]	**K26**
***Viruses*; *Duplodnaviria*; *Heunggongvirae*; *Uroviricota*; *Caudoviricetes* (Cluster 14)**								
1	**BS46** **(United Kingdom)**	MN276049	AC54 (K9) B05 (K9)	gp47	QEP53229	tailspike protein	[110,126,162,163]	**K9**
2	**vB_AbaM_B9** **(Portugal)**	MH133207	NIPH 201 (K45)	gp69	AWD93192	tail spike protein	[28]	**K30/45**
3	**TaPaz** **(Russia)**	MZ043613	NIPH 601 (K47)	gp78	QVW53859	tailspike protein I	[33]	**K47**
				gp79	QVW53860	tailspike protein II		**K102**
4	**Phab24** **(China)**	MZ477002	XH198	gp164	QXM18609	hypothetical protein	[111]	**-**

* *Acinetobacter* phages are arranged within the table in order of increasing bacterial host capsular type number; ** this column of the table only shows the strain designation without the species name *A. baumannii*; N/A means not available; *** this table contains reference(s) not only to works devoted to the characterization of bacteriophage(s) and/or phage-encoded depolymerase(s), but also reference(s) to works on the characterization of the capsule type of bacterial host strain(s), if available; **** **red color:** phage specificity toward a particular *Acinetobacter* K type was determined by the authors; the mechanism of the specific cleavage of *Acinetobacter* CPS by a phage-encoded TSP was established and described; **green color:** prediction of phage specificity toward a particular *Acinetobacter* K type was made by the authors in the cited articles; **blue color:** prediction of phage specificity toward a particular *Acinetobacter* K type was made in this study; **without color:** it was not possible to determine phage specificity toward a particular *Acinetobacter* K type because of the lack of significant similarity of sequences of phage-encoded TSPs with any of the phage depolymerase sequences deposited in Genbank or because of the lack of data on phage bacterial hosts; ***** KL14 was identified in this study.

**Table 6:** TSPs encoded in the genomes of lytic unclassified *Caudoviricetes* phages with siphovirus morphology.

#	Phage Name (Country)	Genbank Accession #	*Acinetobacter* Bacterial Host *	Tailspike Protein			Reference
				# of Gene Product	Genbank Accession #	Annotation in Genbank	
***Viruses*; *Duplodnaviria*; *Heunggongvirae*; *Uroviricota*; *Caudoviricetes* (Cluster 20)**							
1	**53** **(China)**	MW590698 (unverified)	WHG40137	the genome sequence was not annotated by the authors; the coordinates of the gene encoding TSP predicted in this work are as follows: 40833–42812			-
2	**Barton** **(USA)**	MW176032	*A. calcoaceticus* ATCC 23055	gp20	QXO06608	hypothetical protein	-
3	**DMU1** **(USA)**	MT992243	19606	gp20	QOI69765	hypothetical protein	[115]
4	**JeffCo** **(USA)**	MW176034	*A. calcoaceticus* ATCC 23055	gp20	QXO06716	hypothetical protein	-
5	**SH-Ab 15497** **(China)**	MG674163	N/A	gp19	AUG85465	hypothetical protein	[70,116]
***Viruses*; *Duplodnaviria*; *Heunggongvirae*; *Uroviricota*; *Caudoviricetes* (Cluster 22)**							
1	**Effie** **(USA)**	MW176033	*A. calcoaceticus* ATCC 23055	gp24	QXO06658	SGNH/GDSL hydrolase family protein	-

* This column of the table only shows the strain designation without the species name *A. baumannii*; however, if the phage bacterial host does not belong to the species *A. baumannii*, both the species name and the strain designation are indicated; N/A means not available.

## Data Availability

Data are contained within the article and supplementary materials.

## Supplementary Materials

The following supporting information can be downloaded at: https://www.mdpi.com/article/10.3390/v16050771/s1, Figure S1: VIRIDIC-generated heatmap of 233 genomes of *Acinetobacter* phages. Figure S2: Maximum likelihood phylogenetic tree based on 232 sequences of major capsid proteins found in the genomes of *Acinetobacter* phages. Figure S3. (A) Maximum likelihood phylogenetic tree based on the amino acid sequences of the *N-*terminal part of tailspike proteins of cluster 8 phages; (B) Phylogenetic tree based on the DALI structural similarity of tailspike proteins of cluster 8 phages. File S1: Best-ranked structures of tailspike proteins of *Acinetobacter* phages predicted by AlphaFold 2.

## References

[1] Peleg A.Y., Seifert H., Paterson D.L. (2008). *Acinetobacter baumannii*: Emergence of a successful pathogen. Clin. Microbiol. Rev..

[2] Jung J., Park W. (2015). *Acinetobacter* species as model microorganisms in environmental microbiology: Current state and perspectives. Appl. Microbiol. Biotechnol..

[3] Novović K., Jovčić B. (2023). Colistin resistance in *Acinetobacter baumannii*: Molecular mechanisms and epidemiology. Antibiotics.

[4] Denissen J., Reyneke B., Waso-Reyneke M., Havenga B., Barnard T., Khan S., Khan W. (2022). Prevalence of ESKAPE pathogens in the environment: Antibiotic resistance status, community-acquired infection and risk to human health. Int. J. Hyg. Environ. Health.

[5] Santajit S., Indrawattana N. (2016). Mechanisms of antimicrobial resistance in ESKAPE pathogens. BioMed Res. Int..

[6] Chusri S., Chongsuvivatwong V., Rivera J.I., Silpapojakul K., Singkhamanan K., McNeil E., Doi Y. (2014). Clinical outcomes of hospital-acquired infection with *Acinetobacter nosocomialis* and *Acinetobacter pittii*. Antimicrob. Agents Chemother..

[7] Tacconelli E., Carrara E., Savoldi A., Harbarth S., Mendelson M., Monnet D.L., Pulcini C., Kahlmeter G., Kluytmans J., Carmeli Y. (2018). Discovery, research, and development of new antibiotics: The WHO priority list of antibiotic-resistant bacteria and tuberculosis. Lancet Infect. Dis..

[8] Bertozzi Silva J., Storms Z., Sauvageau D. (2016). Host receptors for bacteriophage adsorption. FEMS Microbiol. Lett..

[9] Dunne M., Prokhorov N.S., Loessner M.J., Leiman P.G. (2021). Reprogramming bacteriophage host range: Design principles and strategies for engineering receptor binding proteins. Curr. Opin. Biotechnol..

[10] Taslem Mourosi J., Awe A., Guo W., Batra H., Ganesh H., Wu X., Zhu J. (2022). Understanding bacteriophage tail fiber interaction with host surface receptor: The key “blueprint” for reprogramming phage host range. Int. J. Mol. Sci..

[11] Plattner M., Shneider M.M., Arbatsky N.P., Shashkov A.S., Chizhov A.O., Nazarov S., Prokhorov N.S., Taylor N.M.I., Buth S.A., Gambino M. (2019). Structure and function of the branched receptor-binding complex of bacteriophage CBA120. J. Mol. Biol..

[12] Müller J.J., Barbirz S., Heinle K., Freiberg A., Seckler R., Heinemann U. (2008). An Intersubunit active site between supercoiled parallel beta helices in the trimeric tailspike endorhamnosidase of *Shigella flexneri* phage Sf6. Structure.

[13] Latka A., Leiman P.G., Drulis-Kawa Z., Briers Y. (2019). Modeling the architecture of depolymerase-containing receptor binding proteins in *Klebsiella* phages. Front. Microbiol..

[14] Russo T.A., Luke N.R., Beanan J.M., Olson R., Sauberan S.L., MacDonald U., Schultz L.W., Umland T.C., Campagnari A.A. (2010). The K1 capsular polysaccharide of *Acinetobacter baumannii* strain 307-0294 is a major virulence factor. Infect. Immun..

[15] Harding C.M., Hennon S.W., Feldman M.F. (2018). Uncovering the mechanisms of *Acinetobacter baumannii* virulence. Nat. Rev. Microbiol..

[16] Singh J.K., Adams F.G., Brown M.H. (2019). Diversity and function of capsular polysaccharide in *Acinetobacter baumannii*. Front. Microbiol..

[17] Shashkov A.S., Kenyon J.J., Arbatsky N.P., Shneider M.M., Popova A.V., Miroshnikov K.A., Volozhantsev N.V., Knirel Y.A. (2015). Structures of three different neutral polysaccharides of *Acinetobacter baumannii*, NIPH190, NIPH201, and NIPH615, assigned to K30, K45, and K48 capsule types, respectively, based on capsule biosynthesis gene clusters. Carbohydr. Res..

[18] Shashkov A.S., Kenyon J.J., Senchenkova S.N., Shneider M.M., Popova A.V., Arbatsky N.P., Miroshnikov K.A., Volozhantsev N.V., Hall R.M., Knirel Y.A. (2016). *Acinetobacter baumannii* K27 and K44 capsular polysaccharides have the same K unit but different structures due to the presence of distinct *wzy* genes in otherwise closely related K gene clusters. Glycobiology.

[19] Arbatsky N.P., Kasimova A.A., Shashkov A.S., Shneider M.M., Popova A.V., Shagin D.A., Shelenkov A.A., Mikhailova Y.V., Yanushevich Y.G., Hall R.M. (2022). Involvement of a phage-encoded *wzy* protein in the polymerization of K127 units to form the capsular polysaccharide of *Acinetobacter baumannii* isolate 36-1454. Microbiol. Spectr..

[20] Kasimova A.A., Shashkov A.S., Shneider M.M., Sheck E.A., Mikhailova Y.V., Shelenkov A.A., Popova A.V., Knirel Y.A., Kenyon J.J. (2024). The *Acinetobacter baumannii* K239 capsular polysaccharide includes heptasaccharide units that are structurally related to K86 but joined by different linkages formed by different *wzy* polymerases. Int. J. Biol. Macromol..

[21] Kenyon J.J., Hall R.M. (2013). Variation in the complex carbohydrate biosynthesis loci of *Acinetobacter baumannii* genomes. PLoS ONE.

[22] Cahill S.M., Hall R.M., Kenyon J.J. (2022). An update to the database for *Acinetobacter baumannii* capsular polysaccharide locus typing extends the extensive and diverse repertoire of genes found at and outside the K locus. Microb. Genom..

[23] Kasimova A.A., Kolganova A.S., Shashkov A.S., Shneider M.M., Mikhailova Y.V., Shelenkov A.A., Popova A.V., Knirel Y.A., Perepelov A.V., Kenyon J.J. (2024). Structure of the K141 capsular polysaccharide produced by *Acinetobacter baumannii* isolate KZ1106 that carries KL141 at the chromosomal K locus. Carbohydr. Res..

[24] Roshini J., Patro L.P.P., Sundaresan S., Rathinavelan T. (2023). Structural diversity among *Acinetobacter baumannii* K-antigens and its implication in the in silico serotyping. Front. Microbiol..

[25] Popova A.V., Lavysh D.G., Klimuk E.I., Edelstein M.V., Bogun A.G., Shneider M.M., Goncharov A.E., Leonov S.V., Severinov K.V. (2017). Novel Fri1-like viruses infecting *Acinetobacter baumannii*-vB_AbaP_AS11 and vB_AbaP_AS12-characterization, comparative genomic analysis, and host-recognition strategy. Viruses.

[26] Oliveira H., Costa A.R., Konstantinidis N., Ferreira A., Akturk E., Sillankorva S., Nemec A., Shneider M., Dötsch A., Azeredo J. (2017). Ability of phages to infect *Acinetobacter calcoaceticus*-*Acinetobacter baumannii* complex species through acquisition of different pectate lyase depolymerase domains: Specific genomic pattern variation of phages. Environ. Microbiol..

[27] Lee I.-M., Tu I.-F., Yang F.-L., Ko T.-P., Liao J.-H., Lin N.-T., Wu C.-Y., Ren C.-T., Wang A.H.-J., Chang C.-M. (2017). Structural basis for fragmenting the exopolysaccharide of *Acinetobacter baumannii* by bacteriophage ΦAB6 tailspike protein. Sci. Rep..

[28] Oliveira H., Costa A.R., Ferreira A., Konstantinides N., Santos S.B., Boon M., Noben J.-P., Lavigne R., Azeredo J. (2019). Functional analysis and antivirulence properties of a new depolymerase from a myovirus that infects *Acinetobacter baumannii* capsule K45. J. Virol..

[29] Gordillo Altamirano F., Forsyth J.H., Patwa R., Kostoulias X., Trim M., Subedi D., Archer S.K., Morris F.C., Oliveira C., Kielty L. (2021). Bacteriophage-resistant *Acinetobacter baumannii* are resensitized to antimicrobials. Nat. Microbiol..

[30] Timoshina O.Y., Shneider M.M., Evseev P.V., Shchurova A.S., Shelenkov A.A., Mikhaylova Y.V., Sokolova O.S., Kasimova A.A., Arbatsky N.P., Dmitrenok A.S. (2021). Novel *Acinetobacter baumannii* bacteriophage Aristophanes encoding structural polysaccharide deacetylase. Viruses.

[31] Popova A.V., Shneider M.M., Arbatsky N.P., Kasimova A.A., Senchenkova S.N., Shashkov A.S., Dmitrenok A.S., Chizhov A.O., Mikhailova Y.V., Shagin D.A. (2021). Specific interaction of novel friunavirus phages encoding tailspike depolymerases with corresponding *Acinetobacter baumannii* capsular types. J. Virol..

[32] Liu M., Hernandez-Morales A., Clark J., Le T., Biswas B., Bishop-Lilly K.A., Henry M., Quinones J., Voegtly L.J., Cer R.Z. (2022). Comparative genomics of *Acinetobacter baumannii* and therapeutic bacteriophages from a patient undergoing phage therapy. Nat. Commun..

[33] Shchurova A.S., Shneider M.M., Arbatsky N.P., Shashkov A.S., Chizhov A.O., Skryabin Y.P., Mikhaylova Y.V., Sokolova O.S., Shelenkov A.A., Miroshnikov K.A. (2021). Novel *Acinetobacter baumannii* myovirus TaPaz encoding two tailspike depolymerases: Characterization and host-recognition strategy. Viruses.

[34] Timoshina O.Y., Kasimova A.A., Shneider M.M., Matyuta I.O., Nikolaeva A.Y., Evseev P.V., Arbatsky N.P., Shashkov A.S., Chizhov A.O., Shelenkov A.A. (2023). Friunavirus phage-encoded depolymerases specific to different capsular types of *Acinetobacter baumannii*. Int. J. Mol. Sci..

[35] Pires D.P., Oliveira H., Melo L.D.R., Sillankorva S., Azeredo J. (2016). Bacteriophage-encoded depolymerases: Their diversity and biotechnological applications. Appl. Microbiol. Biotechnol..

[36] Latka A., Maciejewska B., Majkowska-Skrobek G., Briers Y., Drulis-Kawa Z. (2017). Bacteriophage-encoded virion-associated enzymes to overcome the carbohydrate barriers during the infection process. Appl. Microbiol. Biotechnol..

[37] Altschul S.F., Gish W., Miller W., Myers E.W., Lipman D.J. (1990). Basic local alignment search tool. J. Mol. Biol..

[38] AlQuraishi M. (2019). AlphaFold at CASP13. Bioinformatics.

[39] Evans R., O’Neill M., Pritzel A., Antropova N., Senior A., Green T., Žídek A., Bates R., Blackwell S., Yim J. (2021). Protein Complex Prediction with AlphaFold-Multimer. bioRxiv.

[40] Delcher A.L., Bratke K.A., Powers E.C., Salzberg S.L. (2007). Identifying bacterial genes and endosymbiont DNA with Glimmer. Bioinformatics.

[41] Zimmermann L., Stephens A., Nam S.-Z., Rau D., Kübler J., Lozajic M., Gabler F., Söding J., Lupas A.N., Alva V. (2018). A completely reimplemented MPI Bioinformatics Toolkit with a new HHpred server at its core. J. Mol. Biol..

[42] Holm L. (2022). Dali server: Structural unification of protein families. Nucleic Acids Res..

[43] Katoh K., Standley D.M. (2013). MAFFT multiple sequence alignment software version 7: Improvements in performance and usability. Mol. Biol. Evol..

[44] Nguyen L.-T., Schmidt H.A., von Haeseler A., Minh B.Q. (2015). IQ-TREE: A fast and effective stochastic algorithm for estimating maximum-likelihood phylogenies. Mol. Biol. Evol..

[45] Letunic I., Bork P. (2021). Interactive Tree of Life (iTOL) v5: An online tool for phylogenetic tree display and annotation. Nucleic Acids Res..

[46] Holm L., Kääriäinen S., Rosenström P., Schenkel A. (2008). Searching protein structure databases with DaliLite v.3. Bioinformatics.

[47] Moraru C., Varsani A., Kropinski A.M. (2020). VIRIDIC—A novel tool to calculate the intergenomic similarities of prokaryote-infecting viruses. Viruses.

[48] Wyres K.L., Cahill S.M., Holt K.E., Hall R.M., Kenyon J.J. (2020). Identification of *Acinetobacter baumannii* loci for capsular polsaccharide (KL) and lipooligosaccharide outer core (OCL) synthesis in genome assemblies using curated reference databases compatible with Kaptive. Microb. Genom..

[49] Leinonen R., Sugawara H., Shumway M. (2011). International Nucleotide Sequence Database Collaboration. The sequence read archive. Nucleic Acids Res..

[50] Prjibelski A., Antipov D., Meleshko D., Lapidus A., Korobeynikov A. (2020). Using SPAdes de novo assembler. Curr. Protoc. Bioinforma..

[51] Wick R.R., Judd L.M., Gorrie C.L., Holt K.E. (2017). Unicycler: Resolving bacterial genome assemblies from short and long sequencing reads. PLoS Comput. Biol..

[52] Turner D., Kropinski A.M., Adriaenssens E.M. (2021). A roadmap for genome-based phage taxonomy. Viruses.

[53] Margulieux K.R., Bird J.T., Kevorkian R.T., Ellison D.W., Nikolich M.P., Mzhavia N., Filippov A.A. (2023). Complete genome sequence of the broad host range *Acinetobacter baumannii* phage EAb13. Microbiol. Resour. Announc..

[54] Wu N., Dai J., Guo M., Li J., Zhou X., Li F., Gao Y., Qu H., Lu H., Jin J. (2021). Pre-optimized phage therapy on secondary *Acinetobacter baumannii* infection in four critical COVID-19 patients. Emerg. Microbes Infect..

[55] Tan X., Chen K., Jiang Z., Liu Z., Wang S., Ying Y., Zhang J., Yuan S., Huang Z., Gao R. (2024). Evaluation of the impact of repeated intravenous phage doses on mammalian host-phage interactions. J. Virol..

[56] Nir-Paz R., Gelman D., Khouri A., Sisson B.M., Fackler J., Alkalay-Oren S., Khalifa L., Rimon A., Yerushalmy O., Bader R. (2019). Successful treatment of antibiotic-resistant, poly-microbial bone infection with bacteriophages and antibiotics combination. Clin. Infect. Dis..

[57] Huang G., Le S., Peng Y., Zhao Y., Yin S., Zhang L., Yao X., Tan Y., Li M., Hu F. (2013). Characterization and genome sequencing of phage Abp1, a new phiKMV-like virus infecting multidrug-resistant *Acinetobacter baumannii*. Curr. Microbiol..

[58] Bagińska N., Harhala M.A., Cieślik M., Orwat F., Weber-Dąbrowska B., Dąbrowska K., Górski A., Jończyk-Matysiak E. (2023). Biological properties of 12 newly isolated *Acinetobacter baumannii*-specific bacteriophages. Viruses.

[59] Kolsi A., Haukka K., Dougnon V., Agbankpè A.J., Fabiyi K., Virta M., Skurnik M., Kantele A., Kiljunen S. (2023). Isolation and characterization of three novel *Acinetobacter baumannii* phages from beninese hospital wastewater. Arch. Virol..

[60] Liu Y., Mi Z., Mi L., Huang Y., Li P., Liu H., Yuan X., Niu W., Jiang N., Bai C. (2019). Identification and characterization of capsule depolymerase Dpo48 from *Acinetobacter baumannii* phage IME200. PeerJ.

[61] Luo J., Xie L., Yang M., Liu M., Li Q., Wang P., Fan J., Jin J., Luo C. (2023). Synergistic antibacterial effect of phage pB3074 in combination with antibiotics targeting cell wall against multidrug-resistant *Acinetobacter baumannii* in vitro and ex vivo. Microbiol. Spectr..

[62] Mumm I.P., Wood T.L., Chamakura K.R., Kuty Everett G.F. (2013). Complete genome of *Acinetobacter baumannii* podophage Petty. Genome Announc..

[63] Hernandez-Morales A.C., Lessor L.L., Wood T.L., Migl D., Mijalis E.M., Cahill J., Russell W.K., Young R.F., Gill J.J. (2018). Genomic and biochemical characterization of *Acinetobacter* podophage Petty reveals a novel lysis mechanism and tail-associated depolymerase activity. J. Virol..

[64] Hua Y., Luo T., Yang Y., Dong D., Wang R., Wang Y., Xu M., Guo X., Hu F., He P. (2018). Phage therapy as a promising new treatment for lung infection caused by carbapenem-resistant *Acinetobacter baumannii* in mice. Front. Microbiol..

[65] He P., Cao F., Qu Q., Geng H., Yang X., Xu T., Wang R., Jia X., Lu M., Zeng P. (2024). Host range expansion of *Acinetobacter* phage vB_Ab4_Hep4 driven by a spontaneous tail tubular mutation. Front. Cell. Infect. Microbiol..

[66] Essoh C., Vernadet J.-P., Vergnaud G., Coulibaly A., Kakou-N’Douba A., N’Guetta A.S.-P., Resch G., Pourcel C. (2019). Complete genome sequences of five *Acinetobacter baumannii* phages from Abidjan, Côte d’Ivoire. Microbiol. Resour. Announc..

[67] Wintachai P., Surachat K., Singkhamanan K. (2022). Isolation and characterization of a novel *Autographiviridae* phage and its combined effect with tigecycline in controlling multidrug-resistant *Acinetobacter baumannii*-associated skin and soft tissue infections. Viruses.

[68] Merabishvili M., Vandenheuvel D., Kropinski A.M., Mast J., De Vos D., Verbeken G., Noben J.-P., Lavigne R., Vaneechoutte M., Pirnay J.-P. (2014). Characterization of newly isolated lytic bacteriophages active against *Acinetobacter baumannii*. PloS ONE.

[69] Grygorcewicz B., Gliźniewicz M., Rakoczy R., Augustyniak A., Konopacki M., Jabłońska J., Serwin N., Cecerska-Heryć E., Kordas M., Mańkowska K. (2022). PhageScore-based analysis of *Acinetobacter baumannii* infecting phages antibiotic interaction in liquid medium. Arch. Microbiol..

[70] Kasimova A., Arbatsky N., Timoshina O., Shneider M., Shashkov A., Chizhov A., Popova A., Hall R., Kenyon J., Knirel Y. (2021). The K26 capsular polysaccharide from *Acinetobacter baumannii* KZ-1098: Structure and cleavage by a specific phage depolymerase. Int. J. Biol. Macromol..

[71] Popova A.V., Shneider M.M., Myakinina V.P., Bannov V.A., Edelstein M.V., Rubalskii E.O., Aleshkin A.V., Fursova N.K., Volozhantsev N.V. (2019). Characterization of myophage AM24 infecting *Acinetobacter baumannii* of the K9 capsular type. Arch. Virol..

[72] Yuan Y., Li X., Wang L., Li G., Cong C., Li R., Cui H., Murtaza B., Xu Y. (2021). The endolysin of the *Acinetobacter baumannii* phage vB_AbaP_D2 shows broad antibacterial activity. Microb. Biotechnol..

[73] Wu M., Hu K., Xie Y., Liu Y., Mu D., Guo H., Zhang Z., Zhang Y., Chang D., Shi Y. (2019). A novel phage PD-6A3, and its endolysin Ply6A3, with extended lytic activity against *Acinetobacter baumannii*. Front. Microbiol..

[74] Liu Y., Guo X., Shi Y., Tang J., Chen B., Liu F., Fan H., Yan Y., Xu Y. (2016). Characterization of bacteriophage vB_AbaP_PD-AB9 infecting *Acinetobacter baumannii* with broad host range. Chin. J. Lab. Med..

[75] Abdelkader K., Gutiérrez D., Grimon D., Ruas-Madiedo P., Lood C., Lavigne R., Safaan A., Khairalla A.S., Gaber Y., Dishisha T. (2020). Lysin LysMK34 of *Acinetobacter baumannii* bacteriophage PMK34 has a turgor pressure-dependent intrinsic antibacterial activity and reverts colistin resistance. Appl. Environ. Microbiol..

[76] Wintachai P., Phaonakrop N., Roytrakul S., Naknaen A., Pomwised R., Voravuthikunchai S.P., Surachat K., Smith D.R. (2022). Enhanced antibacterial effect of a novel *Friunavirus* phage vWU2001 in combination with colistin against carbapenem-resistant *Acinetobacter baumannii*. Sci. Rep..

[77] Domingues R., Barbosa A., Santos S.B., Pires D.P., Save J., Resch G., Azeredo J., Oliveira H. (2021). Unpuzzling *Friunavirus*-host interactions one piece at a time: Phage recognizes *Acinetobacter pittii* via a new K38 capsule depolymerase. Antibiotics.

[78] Gomes M., Domingues R., Turner D., Oliveira H. (2023). Genomic analysis of two novel bacteriophages infecting *Acinetobacter beijerinckii* and *halotolerans* species. Viruses.

[79] Cheng M., Luo M., Xi H., Zhao Y., Le S., Chen L.-K., Tan D., Guan Y., Wang T., Han W. (2020). The characteristics and genome analysis of vB_ApiP_XC38, a novel phage infecting *Acinetobacter pittii*. Virus Genes.

[80] Ji J.-W., Wang R., Luo T.-T., Xu M.-S., Guo X.-K., Hu F.-P., Li M., He P. (2018). Characterization and genome sequencing of SH-Ab 15599, a novel bacteriophage targeting carbapenem-resistant *Acinetobacter baumannii*. J. Shanghai Jiaotong Univ. Med. Sci..

[81] Buttimer C., O’Sullivan L., Elbreki M., Neve H., McAuliffe O., Ross R.P., Hill C., O’Mahony J., Coffey A. (2016). Genome sequence of jumbo phage vB_AbaM_ME3 of *Acinetobacter baumanni*. Genome Announc..

[82] Vu T.N., Clark J.R., Jang E., D’Souza R., Nguyen L.P., Pinto N.A., Yoo S., Abadie R., Maresso A.W., Yong D. (2024). Appelmans protocol—A directed in vitro evolution enables induction and recombination of prophages with expanded host range. Virus Res..

[83] Yang H., Liang L., Lin S., Jia S. (2010). Isolation and characterization of a virulent bacteriophage AB1 of *Acinetobacter baumannii*. BMC Microbiol..

[84] Yang Z., Liu X., Shi Y., Yin S., Shen W., Chen J., Chen Y., Chen Y., You B., Gong Y. (2019). Characterization and genome annotation of a newly detected bacteriophage infecting multidrug-resistant *Acinetobacter baumannii*. Arch. Virol..

[85] Jiang L., Tan J., Hao Y., Wang Q., Yan X., Wang D., Tuo L., Wei Z., Huang G. (2020). Isolation and characterization of a novel myophage Abp9 against pandrug resistant *Acinetobacater baumannii*. Front. Microbiol..

[86] Huang L., Huang S., Jiang L., Tan J., Yan X., Gou C., Chen X., Xiang L., Wang D., Huang G. (2023). Characterisation and sequencing of the novel phage Abp95, which is effective against multi-genotypes of carbapenem-resistant *Acinetobacter baumannii*. Sci. Rep..

[87] Popova A.V., Zhilenkov E.L., Myakinina V.P., Krasilnikova V.M., Volozhantsev N.V. (2012). Isolation and characterization of wide host range lytic bacteriophage AP22 infecting *Acinetobacter baumannii*. FEMS Microbiol. Lett..

[88] Evseev P.V., Shneider M.M., Kolupaeva L.V., Kasimova A.A., Timoshina O.Y., Perepelov A.V., Shpirt A.M., Shelenkov A.A., Mikhailova Y.V., Suzina N.E. (2024). New *Obolenskvirus* phages Brutus and Scipio: Biology, evolution, and phage-host interaction. Int. J. Mol. Sci..

[89] Zhu Y., Han K., Chen L., Luo S., Fan H., Tong Y. (2022). Biological characterization and genomic analysis of *Acinetobacter baumannii* phage BUCT628. Arch. Virol..

[90] Han K., Zhu Y., Li F., Li M., An X., Song L., Fan H., Tong Y. (2022). Genomic analysis of *Acinetobacter* phage BUCT629, a newly isolated member of the genus *Obolenskvirus*. Arch. Virol..

[91] Evseev P., Gornostal E., Shneider M., Mikhaylova Y., Shelenkov A., Popova A., Miroshnikov K. A novel *Acinetobacter* phage Cato: Lytic myovirus containing tailspike depolymerase. Proceedings of the 2022 Ural-Siberian Conference on Computational Technologies in Cognitive Science, Genomics and Biomedicine (CSGB).

[92] Kim J.H., Oh C., Choresca C.H., Shin S.P., Han J.E., Jun J.W., Heo S.-J., Kang D.-H., Park S.C. (2012). Complete genome sequence of bacteriophage phiAC-1 infecting *Acinetobacter soli* strain KZ-1. J. Virol..

[93] Tan Y., Su J., Luo D., Liang B., Liu S., Zeng H. (2024). Isolation and genome-wide analysis of the novel *Acinetobacter baumannii* bacteriophage vB_AbaM_AB3P2. Arch. Virol..

[94] Wang C., Li P., Zhu Y., Huang Y., Gao M., Yuan X., Niu W., Liu H., Fan H., Qin Y. (2020). Identification of a novel *Acinetobacter baumannii* phage-derived depolymerase and its therapeutic application in mice. Front. Microbiol..

[95] Turner D., Ackermann H.-W., Kropinski A.M., Lavigne R., Sutton J.M., Reynolds D.M. (2018). Comparative analysis of 37 *Acinetobacter* bacteriophages. Viruses.

[96] Wintachai P., Voravuthikunchai S.P. (2022). Characterization of novel lytic *Myoviridae* phage infecting multidrug-resistant *Acinetobacter baumannii* and synergistic antimicrobial efficacy between phage and sacha inchi oil. Pharmaceuticals.

[97] Zhou W., Feng Y., Zong Z. (2018). Two new lytic bacteriophages of the *Myoviridae* family against carbapenem-resistant *Acinetobacter baumannii*. Front. Microbiol..

[98] Jeon J., D’Souza R., Pinto N., Ryu C.-M., Park J., Yong D., Lee K. (2016). Characterization and complete genome sequence analysis of two myoviral bacteriophages infecting clinical carbapenem-resistant *Acinetobacter baumannii* isolates. J. Appl. Microbiol..

[99] Lee C.-N., Tseng T.-T., Lin J.-W., Fu Y.-C., Weng S.-F., Tseng Y.-H. (2011). Lytic myophage Abp53 encodes several proteins similar to those encoded by host *Acinetobacter baumannii* and phage phiKO2. Appl. Environ. Microbiol..

[100] Wintachai P., Surachat K., Chaimaha G., Septama A.W., Smith D.R. (2022). Isolation and characterization of a *Phapecoctavirus* infecting multidrug-resistant *Acinetobacter baumannii* in A549 alveolar epithelial cells. Viruses.

[101] Petrov V.M., Nolan J.M., Bertrand C., Levy D., Desplats C., Krisch H.M., Karam J.D. (2006). Plasticity of the gene functions for DNA replication in the T4-like phages. J. Mol. Biol..

[102] Premetis G.E., Stathi A., Papageorgiou A.C., Labrou N.E. (2023). Characterization of a glycoside hydrolase endolysin from *Acinetobacter baumannii* phage AbTZA1 with high antibacterial potency and novel structural features. FEBS J..

[103] Petrov V.M., Ratnayaka S., Nolan J.M., Miller E.S., Karam J.D. (2010). Genomes of the T4-related bacteriophages as windows on microbial genome evolution. Virol. J..

[104] Peters D.L., Davis C.M., Harris G., Zhou H., Rather P.N., Hrapovic S., Lam E., Dennis J.J., Chen W. (2023). Characterization of virulent T4-like *Acinetobacter baumannii* bacteriophages DLP1 and DLP2. Viruses.

[105] Styles K.M., Thummeepak R., Leungtongkam U., Smith S.E., Christie G.S., Millard A., Moat J., Dowson C.G., Wellington E.M.H., Sitthisak S. (2020). Investigating bacteriophages targeting the opportunistic pathogen *Acinetobacter baumannii*. Antibiotics.

[106] Sitthisak S., Manrueang S., Khongfak S., Leungtongkam U., Thummeepak R., Thanwisai A., Burton N., Dhanoa G.K., Tsapras P., Sagona A.P. (2023). Antibacterial activity of vB_AbaM_PhT2 phage hydrophobic amino acid fusion endolysin, combined with colistin against *Acinetobacter baumannii*. Sci. Rep..

[107] Pulkkinen E., Wicklund A., Oduor J.M.O., Skurnik M., Kiljunen S. (2019). Characterization of vB_ApiM_fHyAci03, a novel lytic bacteriophage that infects clinical *Acinetobacter* strains. Arch Virol..

[108] Jin J., Li Z.-J., Wang S.-W., Wang S.-M., Chen S.-J., Huang D.-H., Zhang G., Li Y.-H., Wang X.-T., Wang J. (2014). Genome organisation of the *Acinetobacter* lytic phage ZZ1 and comparison with other T4-like *Acinetobacter* phages. BMC Genom..

[109] Jeon J., Park J.-H., Yong D. (2019). Efficacy of bacteriophage treatment against carbapenem-resistant *Acinetobacter baumannii* in *Galleria mellonella* larvae and a mouse model of acute pneumonia. BMC Microbiol..

[110] Popova A.V., Shneider M.M., Mikhailova Y.V., Shelenkov A.A., Shagin D.A., Edelstein M.V., Kozlov R.S. (2020). Complete genome sequence of *Acinetobacter baumannii* phage BS46. Microbiol. Resour. Announc..

[111] Loh B., Wang X., Hua X., Chook H.W., Ma L., Zhang L., Manohar P., Jin Y., Leptihn S. (2021). Complete genome sequence of the lytic bacteriophage Phab24, which infects clinical strains of the nosocomial pathogen *Acinetobacter baumannii*. Microbiol. Resour. Announc..

[112] Mardiana M., Teh S.-H., Tsai Y.-C., Yang H.-H., Lin L.C., Lin N.-T. (2023). Characterization of a novel and active temperate phage vB_AbaM_ABMM1 with antibacterial activity against *Acinetobacter baumannii* infection. Sci. Rep..

[113] Tan X., Chen H., Zhang M., Zhao Y., Jiang Y., Liu X., Huang W., Ma Y. (2021). Clinical experience of personalized phage therapy against carbapenem-resistant *Acinetobacter baumannii* lung infection in a patient with chronic obstructive pulmonary disease. Front. Cell. Infect. Microbiol..

[114] Turner D., Wand M.E., Briers Y., Lavigne R., Sutton J.M., Reynolds D.M. (2017). Characterisation and genome sequence of the lytic *Acinetobacter baumannii* bacteriophage vB_AbaS_Loki. PLoS ONE.

[115] Pehde B.M., Niewohner D., Keomanivong F.E., Carruthers M.D. (2021). Genome sequence and characterization of *Acinetobacter* phage DMU1. Phage.

[116] Hua Y., Xu M., Wang R., Zhang Y., Zhu Z., Guo M., He P. (2019). Characterization and whole genome analysis of a novel bacteriophage SH-Ab 15497 against multidrug resistant *Acinetobacater baummanii*. Acta Biochim. Biophys. Sin..

[117] Mardiana M., Teh S.-H., Lin L.-C., Lin N.-T. (2022). Isolation and characterization of a novel *Siphoviridae* phage, vB_AbaS_TCUP2199, infecting multidrug-resistant *Acinetobacter baumannii*. Viruses.

[118] Kim K., Islam M.M., Kim D., Yun S.H., Kim J., Lee J.C., Shin M. (2021). Characterization of a novel phage ΦAb1656-2 and its endolysin with higher antimicrobial activity against multidrug-resistant *Acinetobacter baumannii*. Viruses.

[119] Jeon J., D’Souza R., Pinto N., Ryu C.-M., Park J., Yong D., Lee K. (2015). Complete genome sequence of the siphoviral bacteriophage Βϕ-R3177, which lyses an OXA-66-producing carbapenem-resistant *Acinetobacter baumannii* isolate. Arch. Virol..

[120] Jeon J., Kim J., Yong D., Lee K., Chong Y. (2012). Complete genome sequence of the podoviral bacteriophage YMC/09/02/B1251 ABA BP, which causes the lysis of an OXA-23-producing carbapenem-resistant *Acinetobacter baumannii* isolate from a septic patient. J. Virol..

[121] Badawy S., Pajunen M., Haiko J., Baka Z., Abou-Dobara M., El-Sayed A., Skurnik M. (2020). Identification and functional analysis of temperate *Siphoviridae* bacteriophages of *Acinetobacter baumannii*. Viruses.

[122] Turner D., Wand M.E., Sutton J.M., Centron D., Kropinski A.M., Reynolds D.M. (2016). Genome sequence of vB_AbaS_TRS1, a viable prophage isolated from *Acinetobacter baumannii* strain A118. Genome Announc..

[123] Pas C., Latka A., Fieseler L., Briers Y. (2023). Phage tailspike modularity and horizontal gene transfer reveals specificity towards *E. coli* O-antigen serogroups. Virol. J..

[124] Steinbacher S., Miller S., Baxa U., Budisa N., Weintraub A., Seckler R., Huber R. (1997). Phage P22 tailspike protein: Crystal structure of the head-binding domain at 2.3 Å, fully refined structure of the endorhamnosidase at 1.56 Å resolution, and the molecular basis of O-antigen recognition and cleavage. J. Mol. Biol..

[125] Greenfield J., Shang X., Luo H., Zhou Y., Linden S.B., Heselpoth R.D., Leiman P.G., Nelson D.C., Herzberg O. (2020). Structure and function of bacteriophage CBA120 ORF211 (TSP2), the determinant of phage specificity towards *E. coli* O157:H7. Sci. Rep..

[126] Knirel Y.A., Shneider M.M., Popova A.V., Kasimova A.A., Senchenkova S.N., Shashkov A.S., Chizhov A.O. (2020). Mechanisms of *Acinetobacter baumannii* capsular polysaccharide cleavage by phage depolymerases. Biochem. Biokhimiia.

[127] Lai M.-J., Chang K.-C., Huang S.-W., Luo C.-H., Chiou P.-Y., Wu C.-C., Lin N.-T. (2016). The tail associated protein of *Acinetobacter baumannii* phage ΦAB6 is the host specificity determinant possessing exopolysaccharide depolymerase activity. PLoS ONE.

[128] Abdelkader K., Gutiérrez D., Latka A., Boeckaerts D., Drulis-Kawa Z., Criel B., Gerstmans H., Safaan A., Khairalla A.S., Gaber Y. (2022). The specific capsule depolymerase of phage PMK34 sensitizes *Acinetobacter baumannii* to serum killing. Antibiotics.

[129] Senchenkova S.N., Shashkov A.S., Shneider M.M., Arbatsky N.P., Popova A.V., Miroshnikov K.A., Volozhantsev N.V., Knirel Y.A. (2014). Structure of the capsular polysaccharide of *Acinetobacter baumannii* ACICU containing di-N-acetylpseudaminic acid. Carbohydr. Res..

[130] Kasimova A.A., Shneider M.M., Arbatsky N.P., Popova A.V., Shashkov A.S., Miroshnikov K.A., Balaji V., Biswas I., Knirel Y.A. (2017). Structure and gene cluster of the K93 capsular polysaccharide of *Acinetobacter baumannii* B11911 containing 5-N-Acetyl-7-N-[(R)-3-hydroxybutanoyl]pseudaminic acid. Biochem. Biokhimiia.

[131] Grygorcewicz B., Roszak M., Golec P., Śleboda-Taront D., Łubowska N., Górska M., Jursa-Kulesza J., Rakoczy R., Wojciuk B., Dołęgowska B. (2020). Antibiotics act with vB_AbaP_AGC01 phage against *Acinetobacter baumannii* in human heat-inactivated plasma blood and *Galleria mellonella* models. Int. J. Mol. Sci..

[132] Kenyon J.J., Arbatsky N.P., Sweeney E.L., Shashkov A.S., Shneider M.M., Popova A.V., Hall R.M., Knirel Y.A. (2019). Production of the K16 capsular polysaccharide by *Acinetobacter baumannii* ST25 isolate D4 involves a novel glycosyltransferase encoded in the KL16 gene cluster. Int. J. Biol. Macromol..

[133] Kenyon J.J., Shneider M.M., Senchenkova S.N., Shashkov A.S., Siniagina M.N., Malanin S.Y., Popova A.V., Miroshnikov K.A., Hall R.M., Knirel Y.A. (2016). K19 capsular polysaccharide of *Acinetobacter baumannii* is produced via a *wzy* polymerase encoded in a small genomic island rather than the KL19 capsule gene cluster. Microbiology.

[134] Cahill S.M., Arbatsky N.P., Shashkov A.S., Shneider M.M., Popova A.V., Hall R.M., Kenyon J.J., Knirel Y.A. (2020). Elucidation of the K32 capsular polysaccharide structure and characterization of the KL32 gene cluster of *Acinetobacter baumannii* LUH5549. Biochem. Biokhimiia.

[135] Arbatsky N.P., Shneider M.M., Kenyon J.J., Shashkov A.S., Popova A.V., Miroshnikov K.A., Volozhantsev N.V., Knirel Y.A. (2015). Structure of the neutral capsular polysaccharide of *Acinetobacter baumannii* NIPH146 that carries the KL37 capsule gene cluster. Carbohydr. Res..

[136] Timoshina O.Y., Kasimova A.A., Shneider M.M., Arbatsky N.P., Shashkov A.S., Shelenkov A.A., Mikhailova Y.V., Popova A.V., Hall R.M., Knirel Y.A. (2023). Loss of a branch sugar in the *Acinetobacter baumannii* K3-type capsular polysaccharide due to frameshifts in the *gtr6* glycosyltransferase gene leads to susceptibility to phage APK37.1. Microbiol. Spectr..

[137] Kenyon J.J., Kasimova A.A., Sviridova A.N., Shpirt A.M., Shneider M.M., Mikhaylova Y.V., Shelenkov A.A., Popova A.V., Perepelov A.V., Shashkov A.S. (2021). Correlation of *Acinetobacter baumannii* K144 and K86 capsular polysaccharide structures with genes at the K locus reveals the involvement of a novel multifunctional rhamnosyltransferase for structural synthesis. Int. J. Biol. Macromol..

[138] Arbatsky N.P., Popova A.V., Shneider M.M., Shashkov A.S., Hall R.M., Kenyon J.J., Knirel Y.A. (2021). Structure of the K87 capsular polysaccharide and KL87 gene cluster of *Acinetobacter baumannii* LUH5547 reveals a heptasaccharide repeating unit. Carbohydr. Res..

[139] Arbatsky N.P., Shashkov A.S., Shneider M.M., Popova A.V., Kasimova A.A., Miroshnikov K.A., Knirel Y.A., Hall R.M., Kenyon J.J. (2022). The K89 capsular polysaccharide produced by *Acinetobacter baumannii* LUH5552 consists of a pentameric repeat-unit that includes a 3-acetamido-3,6-dideoxy-d-galactose residue. Int. J. Biol. Macromol..

[140] Shashkov A.S., Cahill S.M., Arbatsky N.P., Westacott A.C., Kasimova A.A., Shneider M.M., Popova A.V., Shagin D.A., Shelenkov A.A., Mikhailova Y.V. (2019). *Acinetobacter baumannii* K116 capsular polysaccharide structure is a hybrid of the K14 and revised K37 structures. Carbohydr. Res..

[141] Arbatsky N.P., Kasimova A.A., Shashkov A.S., Shneider M.M., Popova A.V., Shagin D.A., Shelenkov A.A., Mikhailova Y.V., Yanushevich Y.G., Azizov I.S. (2019). Structure of the K128 capsular polysaccharide produced by *Acinetobacter baumannii* KZ-1093 from Kazakhstan. Carbohydr. Res..

[142] Chang K.-C., Lin N.-T., Hu A., Lin Y.-S., Chen L.-K., Lai M.-J. (2011). Genomic analysis of bacteriophage ϕAB1, a ϕKMV-like virus infecting multidrug-resistant *Acinetobacter baumannii*. Genomics.

[143] Zhang J., Liu X., Li X.-J. (2015). Bioinformatic analysis of phage AB3, a phiKMV-like virus infecting *Acinetobacter baumannii*. Genet. Mol. Res. GMR.

[144] Senchenkova S.N., Kenyon J.J., Jia T., Popova A.V., Shneider M.M., Kasimova A.A., Shashkov A.S., Liu B., Hall R.M., Knirel Y.A. (2019). The K90 capsular polysaccharide produced by *Acinetobacter baumannii* LUH5553 contains di-N-acetylpseudaminic acid and is structurally related to the K7 polysaccharide from *A. baumannii* LUH5533. Carbohydr. Res..

[145] Han P., Pu M., Li Y., Fan H., Tong Y. (2023). Characterization of bacteriophage BUCT631 lytic for K1 *Klebsiella pneumoniae* and its therapeutic efficacy in *Galleria mellonella* larvae. Virol. Sin..

[146] Solovieva E.V., Myakinina V.P., Kislichkina A.A., Krasilnikova V.M., Verevkin V.V., Mochalov V.V., Lev A.I., Fursova N.K., Volozhantsev N.V. (2018). Comparative genome analysis of novel Podoviruses lytic for hypermucoviscous *Klebsiella pneumoniae* of K1, K2, and K57 capsular types. Virus Res..

[147] Tu I.-F., Lin T.-L., Yang F.-L., Lee I.-M., Tu W.-L., Liao J.-H., Ko T.-P., Wu W.-J., Jan J.-T., Ho M.-R. (2022). Structural and biological insights into *Klebsiella pneumoniae* surface polysaccharide degradation by a bacteriophage K1 lyase: Implications for clinical use. J. Biomed. Sci..

[148] Sørensen A.N., Woudstra C., Sørensen M.C.H., Brøndsted L. (2021). Subtypes of tail spike proteins predicts the host range of *Ackermannviridae* phages. Comput. Struct. Biotechnol. J..

[149] Kasimova A.A., Kenyon J.J., Arbatsky N.P., Shashkov A.S., Popova A.V., Knirel Y.A., Hall R.M. (2018). Structure of the K82 capsular polysaccharide from *Acinetobacter baumannii* LUH5534 containing a d-galactose 4,6-pyruvic acid acetal. Biochem. Biokhimiia.

[150] Shashkov A.S., Shneider M.M., Senchenkova S.N., Popova A.V., Nikitina A.S., Babenko V.V., Kostryukova E.S., Miroshnikov K.A., Volozhantsev N.V., Knirel Y.A. (2015). Structure of the capsular polysaccharide of *Acinetobacter baumannii* 1053 having the KL91 capsule biosynthesis gene locus. Carbohydr. Res..

[151] Li P., Chen B., Song Z., Song Y., Yang Y., Ma P., Wang H., Ying J., Ren P., Yang L. (2012). Bioinformatic analysis of the *Acinetobacter baumannii* phage AB1 genome. Gene.

[152] Peng F., Mi Z., Huang Y., Yuan X., Niu W., Wang Y., Hua Y., Fan H., Bai C., Tong Y. (2014). Characterization, sequencing and comparative genomic analysis of vB_AbaM-IME-AB2, a novel lytic bacteriophage that infects multidrug-resistant *Acinetobacter baumannii* clinical isolates. BMC Microbiol..

[153] Guo Z., Huang H., Wu X., Hao Y., Sun Y. (2016). Complete genome sequence of lytic bacteriophage LZ35 infecting *Acinetobacter baumannii* isolates. Genome Announc..

[154] Reid A.J., Eade C., Jones K.J., Jorgenson M.A., Troutman J.M. (2021). Tracking colanic acid repeat unit formation from stepwise biosynthesis inactivation in *Escherichia coli*. Biochemistry.

[155] Li S., Xu X., Lv X., Liu Y., Li J., Du G., Liu L. (2022). Combinatorial metabolic engineering and enzymatic catalysis enable efficient production of colanic acid. Microorganisms.

[156] Chen J., Lee S.M., Mao Y. (2004). Protective effect of exopolysaccharide colanic acid of *Escherichia coli* O157:H7 to osmotic and oxidative stress. Int. J. Food Microbiol..

[157] Mao Y., Doyle M.P., Chen J. (2001). Insertion mutagenesis of *wca* reduces acid and heat tolerance of enterohemorrhagic *Escherichia coli* O157:H7. J. Bacteriol..

[158] Ophir T., Gutnick D.L. (1994). A role for exopolysaccharides in the protection of microorganisms from desiccation. Appl. Environ. Microbiol..

[159] Prigent-Combaret C., Prensier G., Le Thi T.T., Vidal O., Lejeune P., Dorel C. (2000). Developmental pathway for biofilm formation in curli-producing *Escherichia coli* strains: Role of flagella, curli and colanic acid. Environ. Microbiol..

[160] Oliveira H., Domingues R., Evans B., Sutton J.M., Adriaenssens E.M., Turner D. (2022). Genomic diversity of bacteriophages infecting the genus *Acinetobacter*. Viruses.

[161] Schulz E.C., Schwarzer D., Frank M., Stummeyer K., Mühlenhoff M., Dickmanns A., Gerardy-Schahn R., Ficner R. (2010). Structural basis for the recognition and cleavage of polysialic acid by the bacteriophage K1F tailspike protein EndoNF. J. Mol. Biol..

[162] Soothill J.S. (1992). Treatment of experimental infections of mice with bacteriophages. J. Med. Microbiol..

[163] Obana Y., Nishino T., Tanino T. (1985). In-vitro and in-vivo activities of antimicrobial agents against *Acinetobacter calcoaceticus*. J. Antimicrob. Chemother..

