# Supplementary figures and images for: Lytic Capsule-Specific Acinetobacter Bacteriophages Encoding Polysaccharide-Degrading Enzymes
Source: Viruses. 2024 May 13;16(5):771. doi: 10.3390/v16050771 (PMC11126041; doi:10.3390/v16050771)

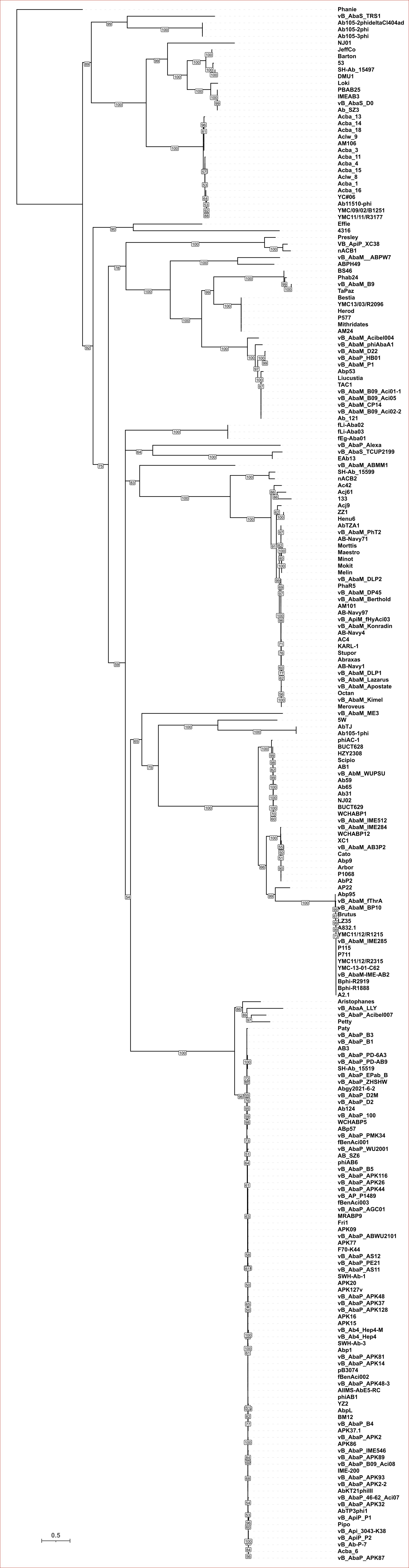

Supplement: Supplementary file 1 [file viruses-16-00771-s001.zip › Suppl_Fig_S2.jpg]

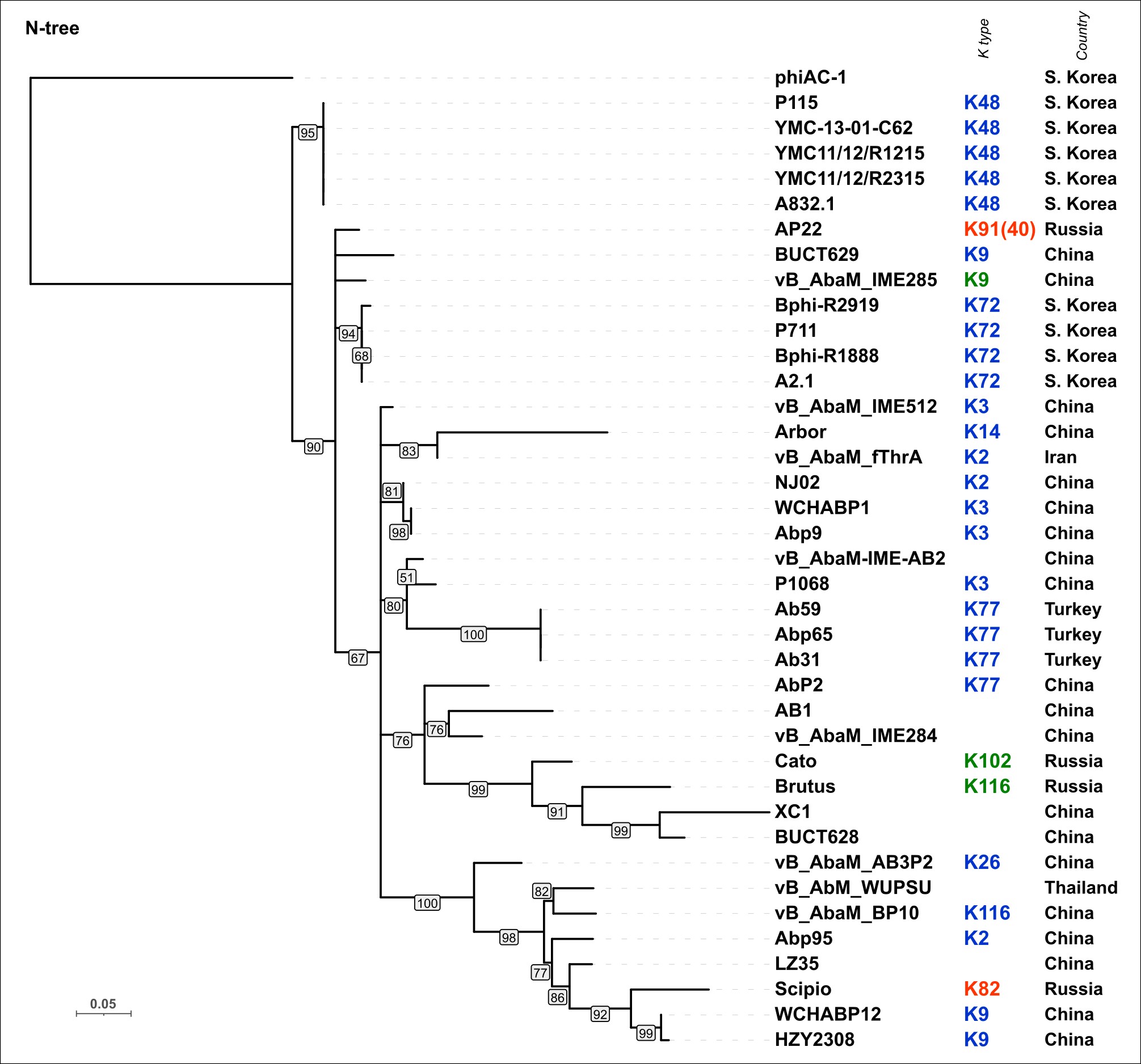

Supplement: Supplementary file 1 [file viruses-16-00771-s001.zip › Suppl_Fig_S3_a.jpg]

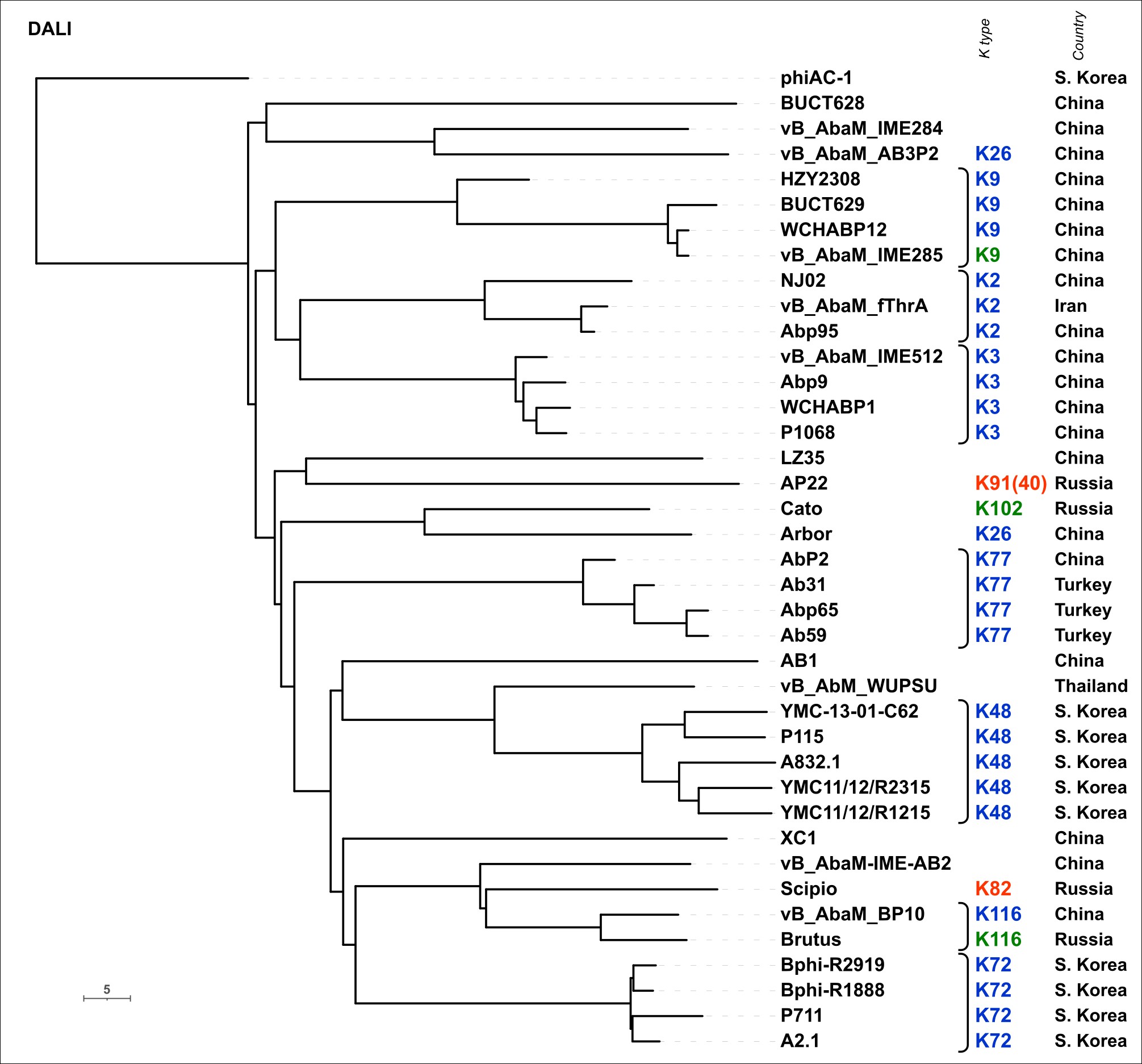

Supplement: Supplementary file 1 [file viruses-16-00771-s001.zip › Suppl_Fig_S3_b.jpg]
